# Identification of hub immune-related genes and construction of predictive models for systemic lupus erythematosus by bioinformatics combined with machine learning

**DOI:** 10.3389/fmed.2025.1557307

**Published:** 2025-05-14

**Authors:** Su Zhang, Weitao Hu, Yuchao Tang, Hongjie Lin, Xiaoqing Chen

**Affiliations:** ^1^Department of Rheumatology, The Second Affiliated Hospital of Fujian Medical University, Quanzhou, China; ^2^Department of Gastroenterology, The Second Affiliated Hospital of Fujian Medical University, Quanzhou, China

**Keywords:** bioinformatics, hub genes, immune cell, machine learning, systemic lupus erythematosus

## Abstract

Systemic lupus erythematosus (SLE) is a chronic autoimmune disease that involves multiple systems. SLE is characterized by the production of autoantibodies and inflammatory tissue damage. This study further explored the role of immune-related genes in SLE. We downloaded the expression profiles of GSE50772 using the Gene Expression Omnibus (GEO) database for differentially expressed genes (DEGs) in SLE. The DEGs were also analyzed for Gene Ontology (GO) and Kyoto Encyclopedia of Genes and Genomes (KEGG) enrichment. The gene modules most closely associated with SLE were then derived by Weighted Gene Co-expression Network Analysis (WGCNA). Differentially expressed immune-related genes (DE-IRGs) in SLE were obtained by DEGs, key gene modules and IRGs. The protein–protein interaction (PPI) network was constructed through the STRING database. Three machine learning algorithms were applied to DE-IRGs to screen for hub DE-IRGs. Then, we constructed a diagnostic model. The model was validated by external cohort GSE61635 and peripheral blood mononuclear cells (PBMC) from SLE patients. Immune cell abundance assessment was achieved by CIBERSORT. The hub DE-IRGs and miRNA networks were made accessible through the NetworkAnalyst database. We screened 945 DEGs, which are closely related to the type I interferon pathway and NOD-like receptor signaling pathway. Machine learning identified a total of five hub DE-IRGs (*CXCL2*, *CXCL8*, *FOS*, *NFKBIA*, *CXCR2*), and validated in GSE61635 and PBMC from SLE patients. Immune cell abundance analysis showed that the hub genes may be involved in the development of SLE by regulating immune cells (especially neutrophils). In this study, we identified five hub DE-IRGs in SLE and constructed an effective predictive model. These hub genes are closely associated with immune cell in SLE. These may provide new insights into the immune-related pathogenesis of SLE.

## 1 Introduction

Systemic lupus erythematosus (SLE) is an immune-mediated, complex, chronic systemic disease (1). Approximately 400,000 new cases of SLE are diagnosed globally each year, and it predominantly affects young women (2). Due to its complex pathogenesis and multi-organ involvement, SLE affects patients’ quality of life and can endanger their lives, and may lead to psychological problems such as anxiety and depression (3). Genetics, hormones, and viral infections are all thought to contribute to the pathogenesis of SLE, but these factors ultimately result in immune dysregulation, which produces autoantibodies that lead to tissue damage (4). However, the pathogenesis of SLE is intricate and has not been thoroughly investigated. At this stage, SLE relies on drugs such as hydroxychloroquine and steroids to regulate immune function. Nevertheless, the toxic side effects of these drugs, such as infections, osteoporosis, and cardiovascular risks, should not be ignored (5, 6). Although the use of biologics offers hope for patients with refractory lupus, they are expensive for long-term use and new treatments are urgently needed.

The emergence of bioinformatics provides an effective way for people to process and analyze large datasets. It is capable of parsing data such as genomes and transcriptomes to identify specific biomarkers associated with certain diseases, thus aiding in early diagnosis and risk assessment (7). In recent years machine learning has become an increasingly promising tool for solving complex problems in the biomedical field. When combined with bioinformatics facilitates improved accuracy and reliability in exploring diseases (8).

In this study, a comprehensive bioinformatics analysis incorporating machine learning algorithms was performed to identify hub IRGs and pathways in SLE using the GEO and Immport databases. We then constructed a predictive model for SLE and validated the expression of the hub IRGs and the accuracy of the model using external datasets and RT-qPCR. Subsequently, we investigated a Pearson correlation analysis between hub genes and immune cells. Finally, we identified key miRNA molecules that interact with the hub genes. In summary, the study revealed hub IRGs in SLE, which will help to further elucidate the contribution of immune factors in SLE development and thus provide clues for exploring the complex etiology of SLE.

## 2 Materials and methods

### 2.1 Data collecting

The GEO database^1^ (9) was searched for the keyword “systemic lupus erythematosus” to obtain the SLE-related dataset GSE50772 and GSE61635. GSE50772 was used as the training cohort, while GSE61635 served as the validation group. Both datasets are based on the GPL570 platform. The GSE50772 contains peripheral blood samples from 61 SLE patients and 20 normal controls (NC), while GSE61635 contains 109 blood samples from SLE and NC. In addition, we acquired datasets of primary Sjögren’s Syndrome (pSS, GSE84844) and rheumatoid arthritis (RA, GSE17755). GSE84844 and GSE17755 were used for subsequent assessment of the diagnostic value of the hub genes for pSS and RA. Table 1 provides details of all the datasets.

**Table 1:** Details of the datasets included in this study.

Dataset	Platform	Species	Tissue	Number of cases and controls	Type of cohorts
GSE50772	GPL570	*Homo sapiens*	Peripheral blood	61 SLE/20NC	Training
GSE61635	GPL570			79 SLE/30NC	Validating
GSE84844	GPL570			30 pSS/30NC	Validating
GSE17755	GPL1291			112RA/53 NC	Validating

### 2.2 Identification of DEGs and enrichment analysis

GSE50772 was normalized and filtered for DEGs using the “limma” package. The selection criteria for DEGs were set to|log2 FoldChange| > 0.5, corrected *p* < 0.05. DEGs were displayed using volcano and heatmaps. Gene Ontology (GO) and Kyoto Encyclopedia of Genes and Genomes (KEGG) function analysis of DEGs was conducted with “clusterprofile” package to understand the biological processes and signaling pathways in which they are involved. A corrected *p* < 0.05 was considered to be statistical significance.

### 2.3 Construction of weighted gene co-expression network

To screen key gene modules from different modules that affect the SLE phenotype, we constructed a co-expression network using the “WGCNA” package of R (10). The best soft threshold was first established by pickSoftThreshold function. Then, the module merging threshold was set to 0.25 to obtain co-expression modules. Every module contains a minimum of 20 genes and non-significant genes were grouped into gray module. Finally, the correlation between every gene module and phenotype was computed. The correlation between gene modules and SLE patients was also assessed by the values of gene significance (GS) and module membership (MM).

### 2.4 Acquisition of common genes (CGs) and construction of PPI networks

The common genes (CGs) of DEGs and key gene modules were obtained by Venn diagram. The STRING database^2^ (11) is commonly utilized to construct PPI networks. The minimum required reciprocal score was 0.4. Subsequent visualization was performed with Cytoscape software (version 3.9.1) (12). In addition, the PPI network nodes were scored utilizing Cytoscape’s molecular complex detection (MCODE) plugin to filter out the most important modules and genes. The setup parameters for the MCODE plugin in this study were MCODE score > 5, degree criticality = 2, node score criticality = 0.2, maximum depth = 100, *k*-score = 2.

### 2.5 Identification of DE-IRGs in SLE

There were 1793 IRGs were acquired from the ImmPort database^3^ (13). The Venn diagram showed that overlapping genes of IRGs and CGs are the DE-IRGs in SLE.

### 2.6 Screening of hub DE-IRGs in SLE

The least absolute shrinkage and selection operator (LASSO) regression is usually applied to select features for high-dimensional data, especially in gene expression data analysis (14). The basic principle is to perform variable selection by introducing L1 regularization terms, so as to efficiently screen out the important genes related to the target variables (15). Random forest (RF) is a machine learning method based on integrated learning, widely used in classification and regression problems, and can also be used to screen feature genes. In genomics and bioinformatics, random forests can help select the most relevant gene features to the target variable by assessing the importance of each gene to the prediction model (16). Support vector machine-recursive feature elimination (SVM-RFE) is a machine learning method commonly applied to screen signature genes. It is based on the principle of maximum interval of Support vector machine (SVM), through the model training samples, each feature score sorting, and then use the recursive feature elimination (RFE) algorithm step-by-step iterative way: remove the features with the smallest feature scores, and then use the remaining features to train the model again for the next iteration. The remaining characteristics are then utilized to train the model again for the next iteration, and finally the best combination of features is selected (17). In this study, we screened signature IRGs from 22 DE-IRGs using the three machine learning methods described above. The upset R diagram was subsequently utilized to obtain the intersecting genes of the three methods as the hub DE-IRGs of SLE.

### 2.7 Construction and validation of model

The accuracy of hub DE-IRGs selected by the machine learning methods was validated in another external SLE dataset. Subsequently, a model based on hub genes was constructed with an area under the curve (AUC) was greater than 0.8, indicating that the model has strong diagnostic value. Furthermore, we also assessed the diagnostic worth of the hub genes for pSS and RA by ROC curves.

### 2.8 Acquisition of peripheral blood mononuclear cells (PBMCs)

Peripheral blood was collected from 30 patients diagnosed with SLE from June 2024 to December 2024 in the Department of Rheumatology, the Second Affiliated Hospital of Fujian Medical University. Peripheral blood was also collected from 22 normal controls who excluded hepatitis B, diabetes mellitus, pathogenic infection, malignant tumor and other types of autoimmune diseases, such as RA and pSS. The diagnosis of SLE was based on the European League Against Rheumatism (EULAR)/American College of Rheumatology (ACR) 2019 criteria. Furthermore, SLE disease activity was evaluated based on the SLE disease activity index 2000 (SLEDAI-2K) (18). We also collected gender, age, and relevant clinical and laboratory indicators for all participants (Table 2). Erythrocyte lysate (C3702) and lymphocyte isolate (C0025) were purchased from Beyotime (Shanghai, China). Mononuclear cells from peripheral blood were isolated according to the appropriate instructions (19).

**Table 2:** Clinical traits of SLE patients and normal controls.

Clinical traits*	SLE (*n* = 30)	Normal controls (*n* = 22)
Sex, male/female	2/28	2/20
Age (year)	35.77 ± 15.59	33.25 ± 9.99
Duration (year)	8.63 ± 7.69	
LN	12 (30)	
SLEDAI scores	11.5 ± 3.82	
ANA (positive)	29 (30)	
Anti-dsDNA antibody (positive)	26 (30)	
Lupus anticoagulant (positive)	12 (30)	
Leukocyte (10^9^/L)	5.63 ± 1.79	
Platelets (10^9^/L)	180.65 ± 94.51	
CRP (mg/L)	10.05 ± 13.24	
ESR (mm/h)	20.2 ± 15.69	
C3 (g/L)	0.73 ± 0.45	
C4 (g/L)	0.1 ± 0.05	
IgG (g/L)	15.14 ± 8.05	
IgA (g/L)	2.67 ± 1.8	
IgM (g/L)	0.8 ± 0.63	
Serum creatinine (μmol/L)	72.81 ± 58.24	
24 h urine protein (positive)	12 (30)	

*LN, lupus nephritis; SLEDAI, systemic lupus erythematosus disease activity index; ANA, antinuclear antibody; Anti-dsDNA antibody, anti-double stranded deoxyribonucleic acid antibody; CRP, C-reactive protein; ESR, erythrocyte sedimentation rate; C3/C4, complement 3/complement 4; Igs, immunoglobulins.

### 2.9 RT-qPCR to validate hub genes expression

The RNA extraction kit was purchased from BioTeke (Beijing, China). See reference (19) for specific methodology. Reverse transcription reagents were purchased from Takara (Japan). The cDNA synthetic reaction was run at 37°C for 15 min and then heated at 85°C for 3 min to terminate. The cDNA was subsequently kept in liquid nitrogen. Finally, ABI PRISM 7500 PCR instrument (Applied Biosystems, United States) was used to amplify the target genes. The PCR cycle was performed as follows: 95°C for 15 min, 40 cycles of 95°C for 5 s, and 60°C for 30 s. *B-actin* was used as housekeeping gene to normalize target gene data. The primer sequences are shown in Table 3.

**Table 3:** The primers used in this study.

Gene names	Primers sequences (5′ → 3′)
B-actin-F	CATGTACGTTGCTATCCAGGC
B-actin-R	CTCCTTAATGTCACGCACGAT
CXCL2-F	GGCAGAAAGCTTGTCTCAACCC
CXCL2-R	CTCCTTCAGGAACAGCCACCAA
CXCR2-F	TCCGTCACTGATGTCTACCTGC
CXCR2-R	TCCTTCAGGAGTGAGACCACCT
CXCL8-F	GAGAGTGATTGAGAGTGGACCAC
CXCL8-R	CACAACCCTCTGCACCCAGTTT
FOS-F	GCCTCTCTTACTACCACTCACC
FOS-R	AGATGGCAGTGACCGTGGGAAT
NFKBIA-F	TCCACTCCATCCTGAAGGCTAC
NFKBIA-R	CAAGGACACCAAAAGCTCCACG

### 2.10 Evaluation of immune cell abundance

Since SLE is a classical autoimmune disease, immune cells play an important part in its development. CIBERSORT^4^ utilizes a gene expression matrix from a sample compared to a known set of genes that characterize the cell type using an inverse convolution algorithm to infer the relative level of each type of cell in the sample (20). We obtained the composition of 22 immune cells in SLE by Cibersort. We then compared the difference in the distribution of immune cells between the SLE and NC groups. Subsequent Pearson correlation analysis between hub genes and immune cells was calculated. For the above analysis, *p* < 0.05 represents statistical significance.

### 2.11 Construction of gene-miRNA networks

We uploaded hub genes to NetworkAnalyst website^5^ (21) to get miRNAs closely related to hub genes and constructed their interaction networks.

### 2.12 Statistical analysis

**Figure 1:**
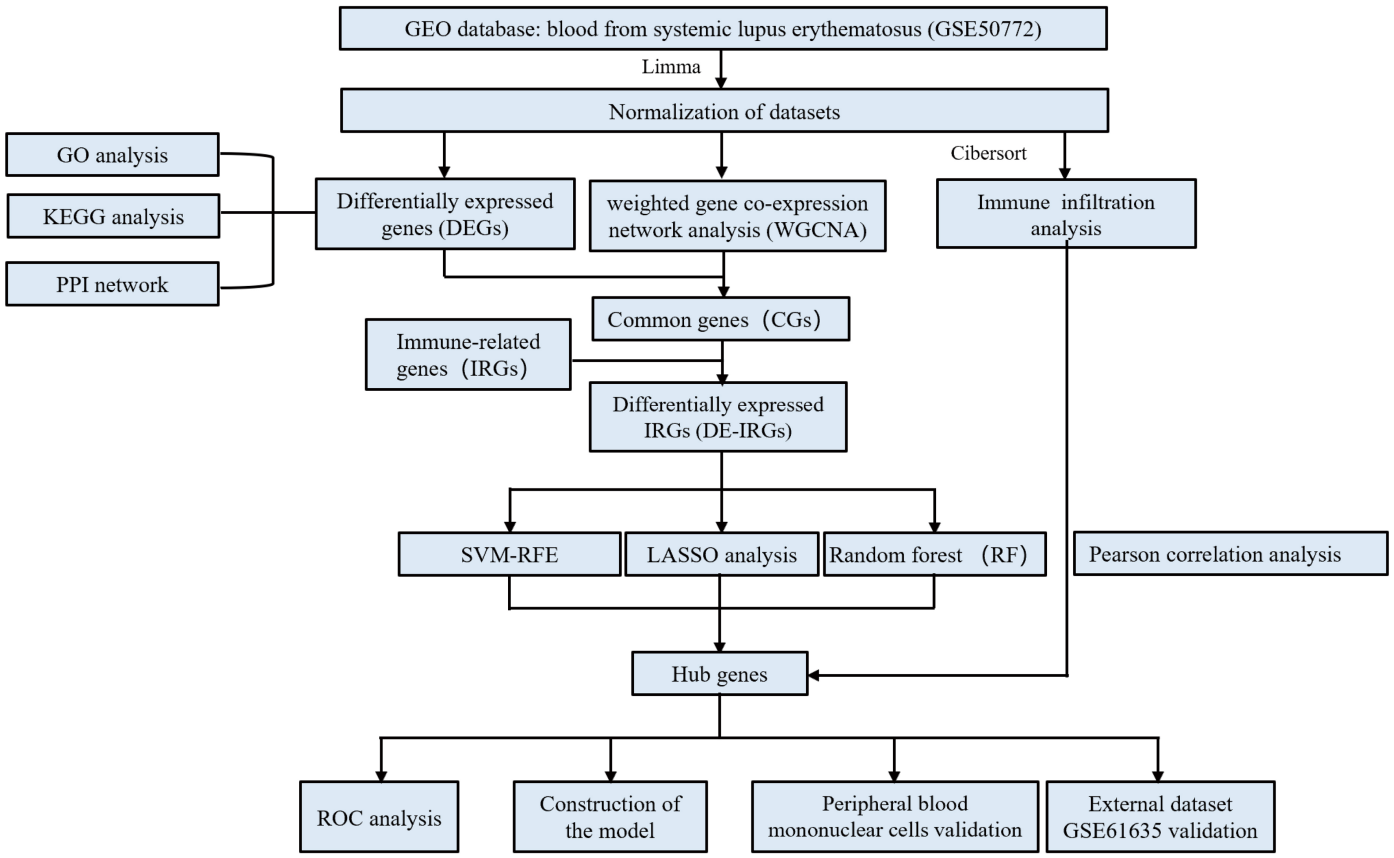
The specific flowchart of this study.

The R software (version 4.4.2) was employed for all analyses. Pearson analysis was applied to investigate the correlation between hub genes and immune cells, and *p* < 0.05 was considered to be statistical significance. The specific flow chart of the study is summarized in Figure 1.

## 3 Results

### 3.1 Acquisition and enrichment analysis of DEGs

**Figure 2:**
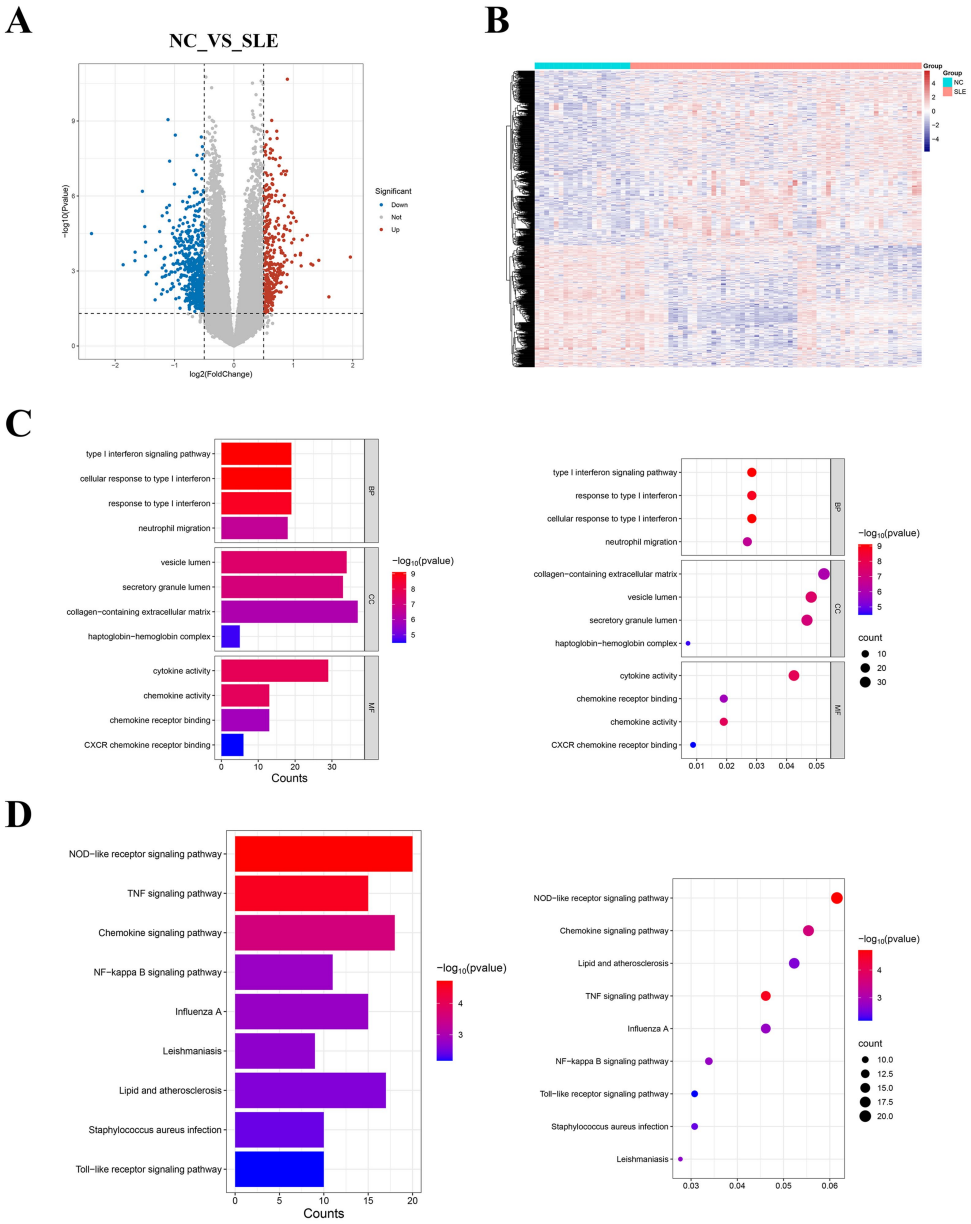
Identification of DEGs in systemic lupus erythematosus (SLE) and enrichment analysis. **(A)** The volcano plot displayed the DEGs. Red represents upregulated genes, while blue represents downregulated genes. **(B)** The heatmap showed the expression of DEGs in normal controls (NC) and SLE. **(C)** Bar and bubble plots of GO enrichment analysis. **(D)** Bar and bubble plots of KEGG enrichment analysis.

The median gene expression of single samples remained consistent after normalization to the training cohort, indicating that potential batch effects were rectified (Supplementary Figure 1). Based on the above selection criteria, we obtained 945 DEGs from GSE50772 (Figure 2A). The heatmap showed that they were expressed differently in NC and SLE groups (Figure 2B). The specific names of the DEGs were given in the Supplementary file. GO analysis showed that DEGs were mainly closely related to the type I interferon (Figure 2C). While KEGG enrichment showed that DEGs were primarily engaged in NOD-like receptor signaling pathway and TNF signaling pathway (Figure 2D). We chose the NOD-like receptor signaling pathway to demonstrate the distribution of DEGs in it (Supplementary Figure 2).

### 3.2 Identification of key module genes

**Figure 3:**
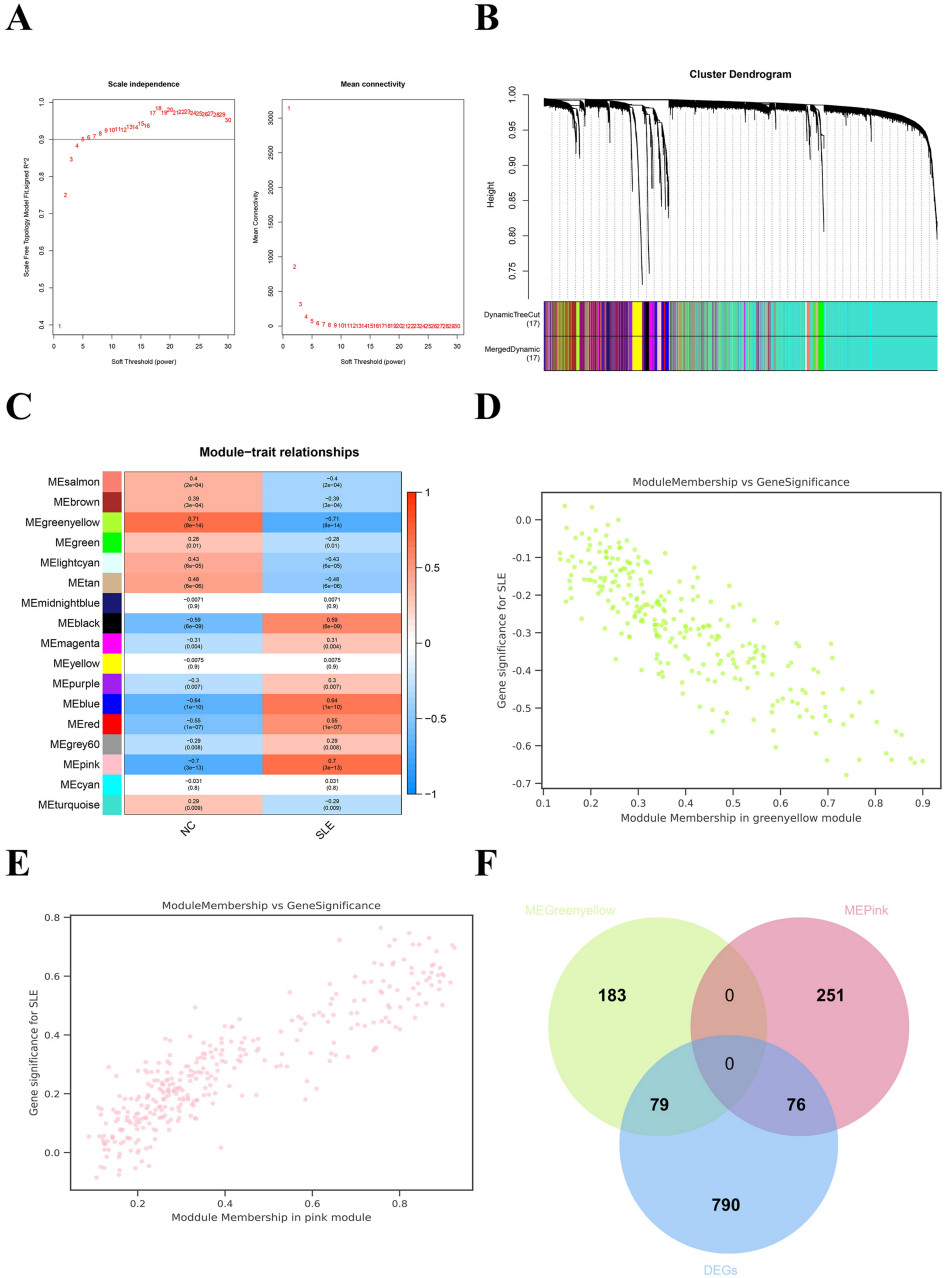
Weighted gene co-expression network analysis (WGCNA) for SLE. **(A)** The soft threshold and mean connectivity of the WGCNA network. **(B)** The clustering dendrogram of the WGCNA network. **(C)** The heatmap depicts the correlation of the different modules with clinical features, especially SLE and NC. **(D)** The scatter plot between the gene significance (GS) and module members (MM) in the green yellow module. **(E)** The scatter plot between the GS and MM in the pink module. **(F)** The Venn plot displayed the common genes (CGs) of yellow-green modular genes, pink modular genes, and DEGs.

Weighted Gene Co-expression Network Analysis showed that the mean connectivity is 0.9 when the soft threshold *β* is 5 (Figure 3A). A total of 17 gene modules were recognized (Figure 3B). We chose modules with a disease correlation coefficient greater than 0.7 as key modules. Green yellow and pink modules were found to fulfill our requirements and they satisfied *p* < 0.05 (Figures 3C–E). We take the intersection of the two modules’ genes and DEGs to get their common genes (CGs). A total of 175 CGs were obtained (Figure 3F). We then uploaded the CGs to the STRING database and visualized the PPI network using Cytoscape. We got a PPI network consisting of 54 points and 247 edges (Supplementary Figure 3A). The most critical module is composed of 20 points and 147 edges (Supplementary Figure 3B). This suggested that CGs work together in the same biological process.

### 3.3 Identification of the hub DE-IRGs in SLE

**Figure 4:**
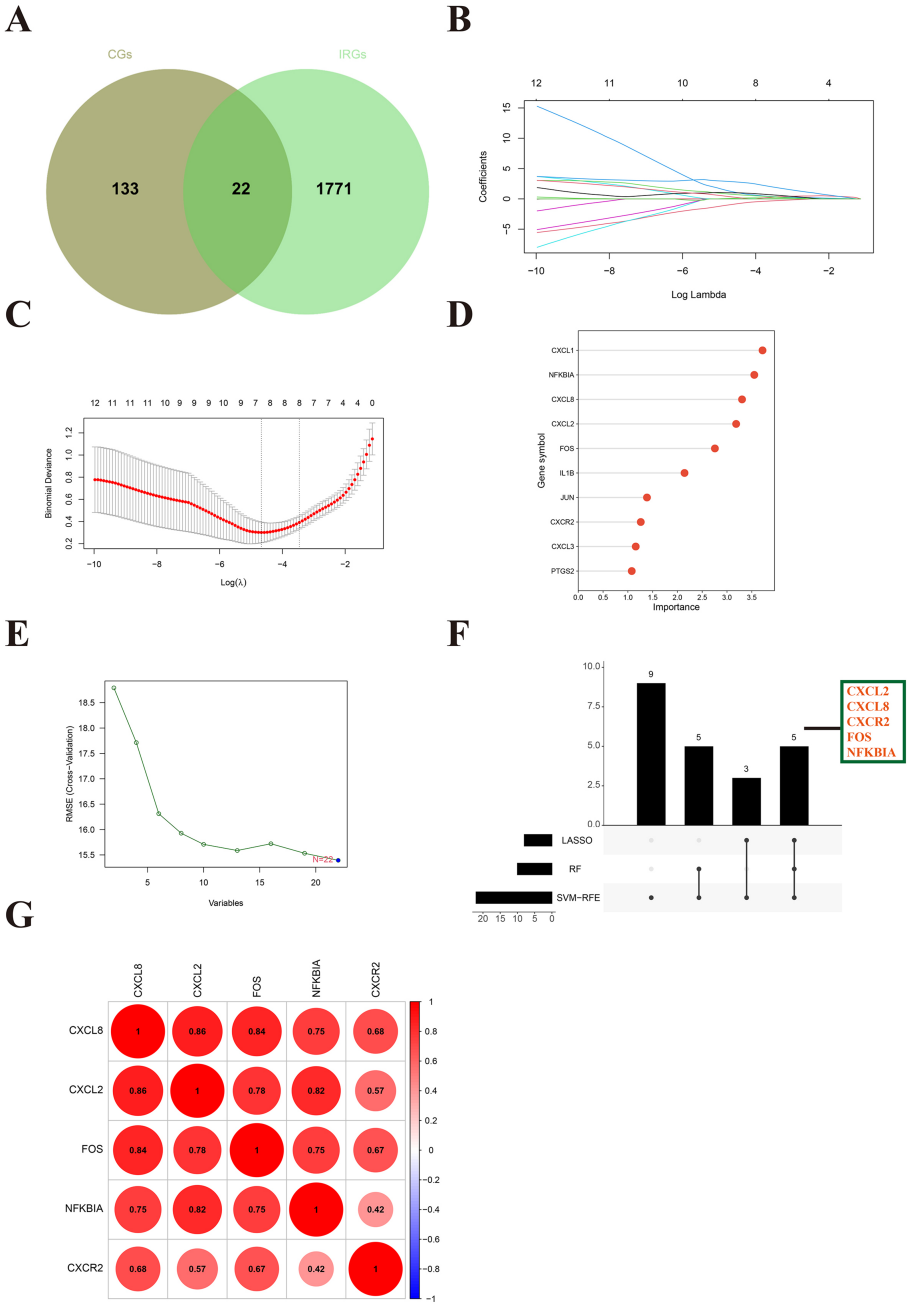
Identification of hub immune-related genes in SLE. **(A)** The Venn diagram showed 22 differentially expressed immune-related genes (DE-IRGs) for SLE. **(B)** Path diagram of LASSO regression coefficients for DE-FRGs in the training set. **(C)** LASSO regression cross-validation curves. A 10-fold cross-validation was used in the training set to determine the optimal *λ* value. **(D)** The lollipop plot illustrates the relative importance of genes in the random forest model in the training set. **(E)** SVM-RFEs algorithm to screen feature genes. **(F)** The upset depicted the hub genes obtained by three machine learning algorithms. **(G)** The heatmap revealed the correlation between hub genes.

The Venn plot identified 22 DE-IRGs in SLE (Figure 4A). Three machine learning methods was applied to screen signature genes. LASSO regression screened eight signature genes (Figures 4B,C). Random Forest ranked the 22 DE-IRGs for importance to get the top 10 genes with the highest scores (Figure 4D). A total of 22 signature genes were obtained from SVM-RFE (Figure 4E). The intersection of the signature genes obtained from the three machine learning algorithms is taken to acquire the final five hub genes (*CXCL2*, *CXCL8*, *FOS*, *NFKBIA*, *CXCR2*) (Figure 4F). The hub genes are positively correlated with each other (Figure 4G). This implied that hub genes are synergistic in some functions.

### 3.4 Construction and validation of models

**Figure 5:**
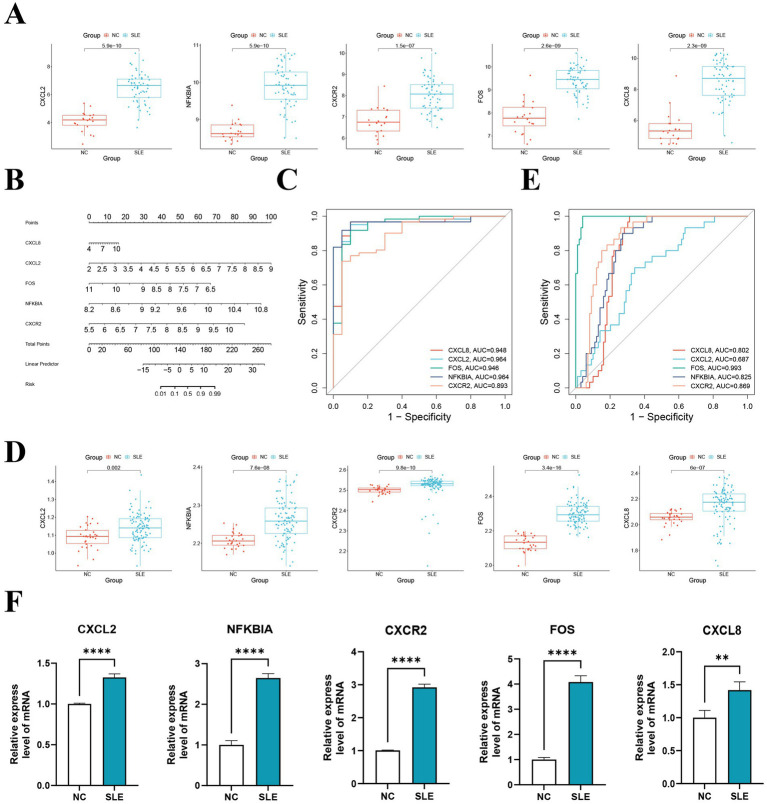
The construction and validation of the model and hub genes. **(A)** Expression levels of hub genes in the training set GSE50772. **(B)** The nomogram illustrated the diagnostic model for diagnosing SLE. **(C)** ROC analysis of five hub genes of the training cohort. **(D)** Expression levels of hub genes in the validation set GSE61635. **(E)** ROC analysis of five hub genes of the validating cohort. **(F)** The hub genes were verified by RT-qPCR of peripheral blood mononuclear cells (PBMC) from SLE patients. **p < 0.01, ****p < 0.0001.

Interestingly, all of the hub genes are upregulated genes in the SLE training cohort (*p* < 0.05) (Figure 5A). We subsequently constructed a nomogram of SLE (Figure 5B). The AUC for ROC was found to be greater than 0.8, indicating that performed well in diagnosing SLE (Figure 5C). We then verified in the external cohort that the expression of hub genes was consistent with the training cohort (Figure 5D). And the accuracy of the model was verified again, and it was found that the AUCs were all greater than 0.8, which more strongly supported our results (Figure 5E). Further RT-qPCR results indicated that the expression of hub genes in the SLE group was obviously higher than that in the NC group (Figure 5F). Then, we detected that the expression of *CXCL8* in the pSS was significantly higher than that in the NC group, while *NFKBIA* was significantly lower than that in the NC group (Supplementary Figure 4A). Whereas in RA, the expression of *CXCL8*, *CXCR2* and *NFKBIA* was markedly higher than that in the NC group (Supplementary Figure 4B). Surprisingly, although the hub genes have some diagnostic value for pSS and RA, their diagnostic efficacy is not as good as that of SLE (AUCs < 0.8) (Supplementary Figures 4C,D). This reinforces the specificity of the hub genes in the diagnosis of SLE.

### 3.5 Immune cell abundance analysis

**Figure 6:**
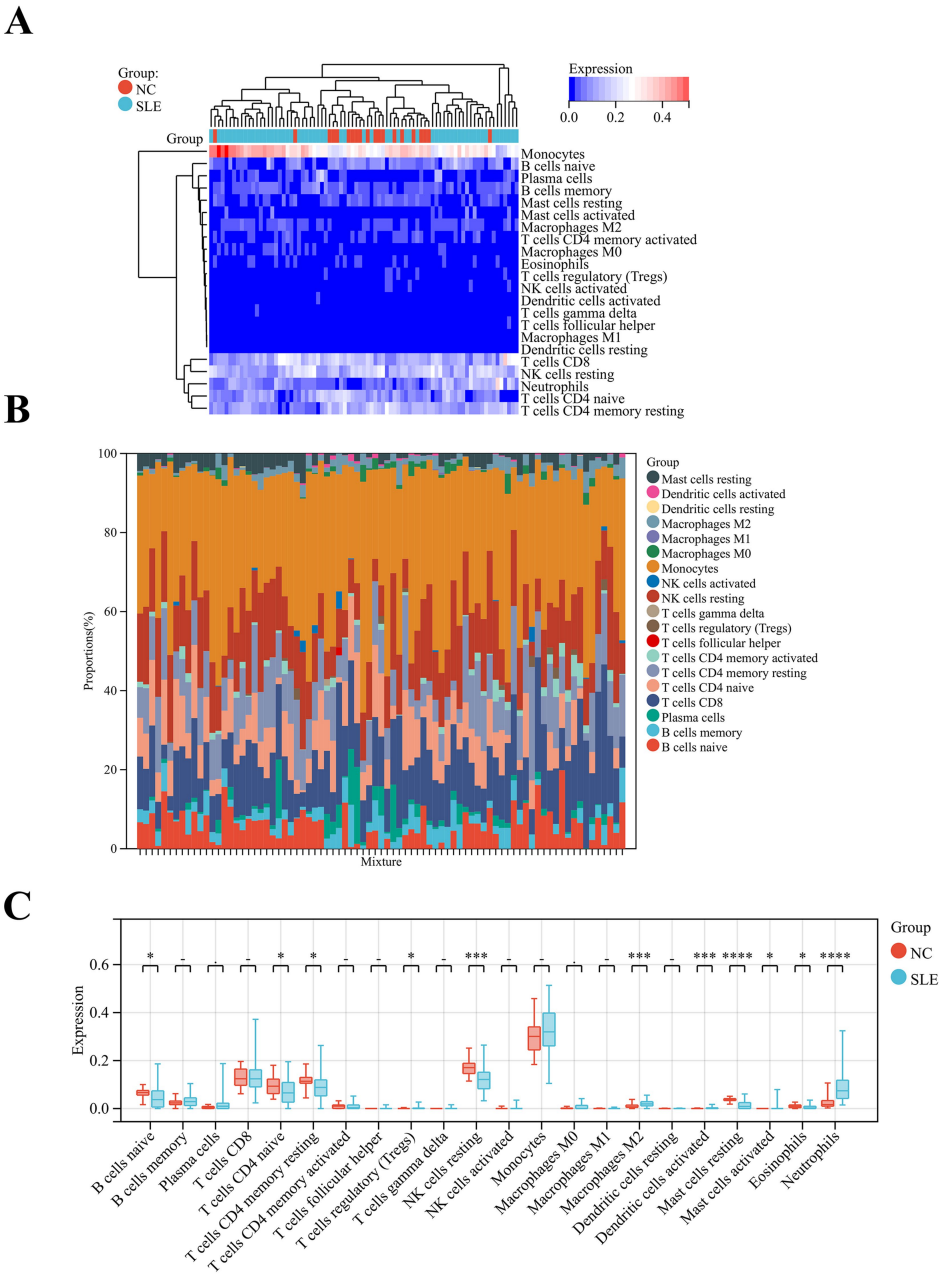
Analysis of immune cell abundance in SLE. **(A)** The heatmap showed the distribution of immune cells in SLE and NC. **(B)** Relative percentage of immune cell subpopulations in SLE and NC. **(C)** The box plot displayed the differences in the levels of immune cells in SLE and NC. *p < 0.05, ***p < 0.001, ****p < 0.0001.

Since SLE is a classical autoimmune disease, immune cells play an essential function in its pathogenesis. Our results showed that monocytes are the major immune component cells in SLE and NC groups (Figure 6A). The second is NK cells resting (Figure 6B). Meanwhile, our results revealed that T cells regulatory (*p* < 0.05), M2 macrophages (*p* < 0.001) and dendritic cells activated (*p* < 0.001), mast cells activated (*p* < 0.01) and neutrophils (*p* < 0.0001) were significantly higher in SLE (Figure 6C). In contrast, B cells naive (*p* < 0.05), T cells CD4 naive (*p* < 0.05), T cells CD4 memory resting (*p* < 0.05), NK cells resting (*p* < 0.001), mast cells resting (*p* < 0.0001) and eosinophils (*p* < 0.05) were significantly lower in SLE than in NC (Figure 6C). These results suggest that M2 macrophages and T cells are the major immune component cells in SLE patients. These cells may play an important role in the pathogenesis of SLE (22).

### 3.6 Correlation between the hub genes and immune cells

**Figure 7:**
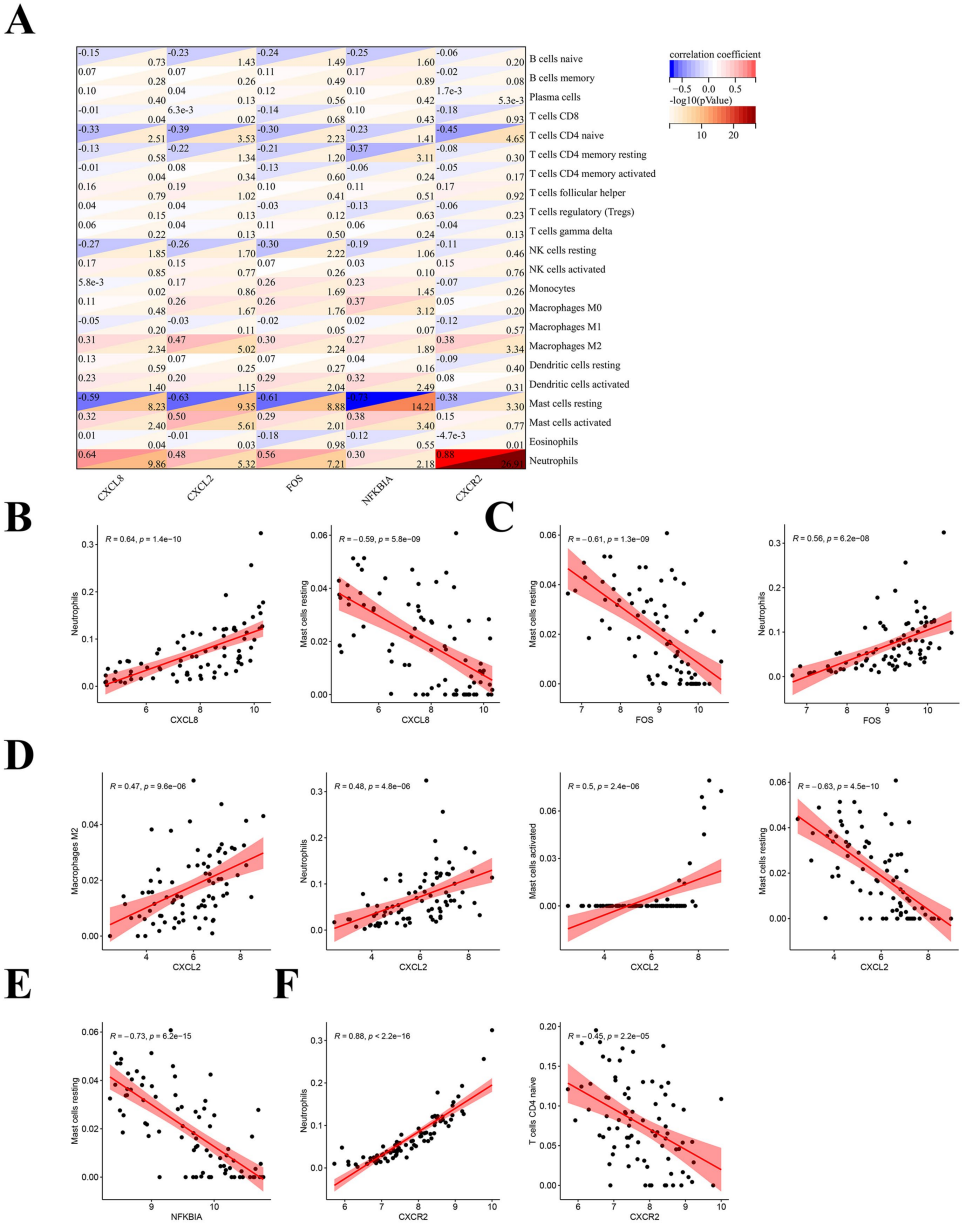
Correlation analysis of hub genes with immune cells. **(A)** Pearson correlation analysis of hub immune-related genes in SLE with immune cells. **(B)** Correlation analysis of CXCL8 with immune cells. **(C)** Correlation analysis of FOS with immune cells. **(D)** Correlation analysis of CXCL2 with immune cells. **(E)** Correlation analysis of NFKBIA with immune cells. **(F)** Correlation analysis of CXCR2 with immune cells.

In order to further understand the relationship between hub genes and immune cells, we performed Pearson correlation analysis on them. The results of the analysis showed that all hub genes showed strong correlation with a variety of immune cells (Figure 7A). Specifically, *CXCL8* was positively correlated with neutrophils (*R* = 0.64) and negatively correlated with mast cells resting (*R* = −0.59) (Figure 7B). *FOS* correlated with immune cells in the same way as CXCL8, also positively with neutrophils (*R* = 0.56) and negatively with mast cells resting (*R* = −0.61) (Figure 7C). *CXCL2* was positively related to multiple immune cells, which included M2 macrophages (*R* = 0.47), neutrophils (*R* = 0.48), and activated mast cells (*R* = 0.5), while it was negatively associated with mast cells resting (*R* = −0.63) (Figure 7D). *NFKBIA* showed a strong negative correlation with mast cells resting (*R* = −0.73) (Figure 7E). *CXCR2* possessed the strongest positive correlation with neutrophils (*R* = 0.88) and was negatively correlated with CD4^+^T cells naive (*R* = −0.45) (Figure 7F). These suggest that hub genes are strongly associated with immune cells in SLE.

### 3.7 Construction of hub genes-miRNA network

**Figure 8:**
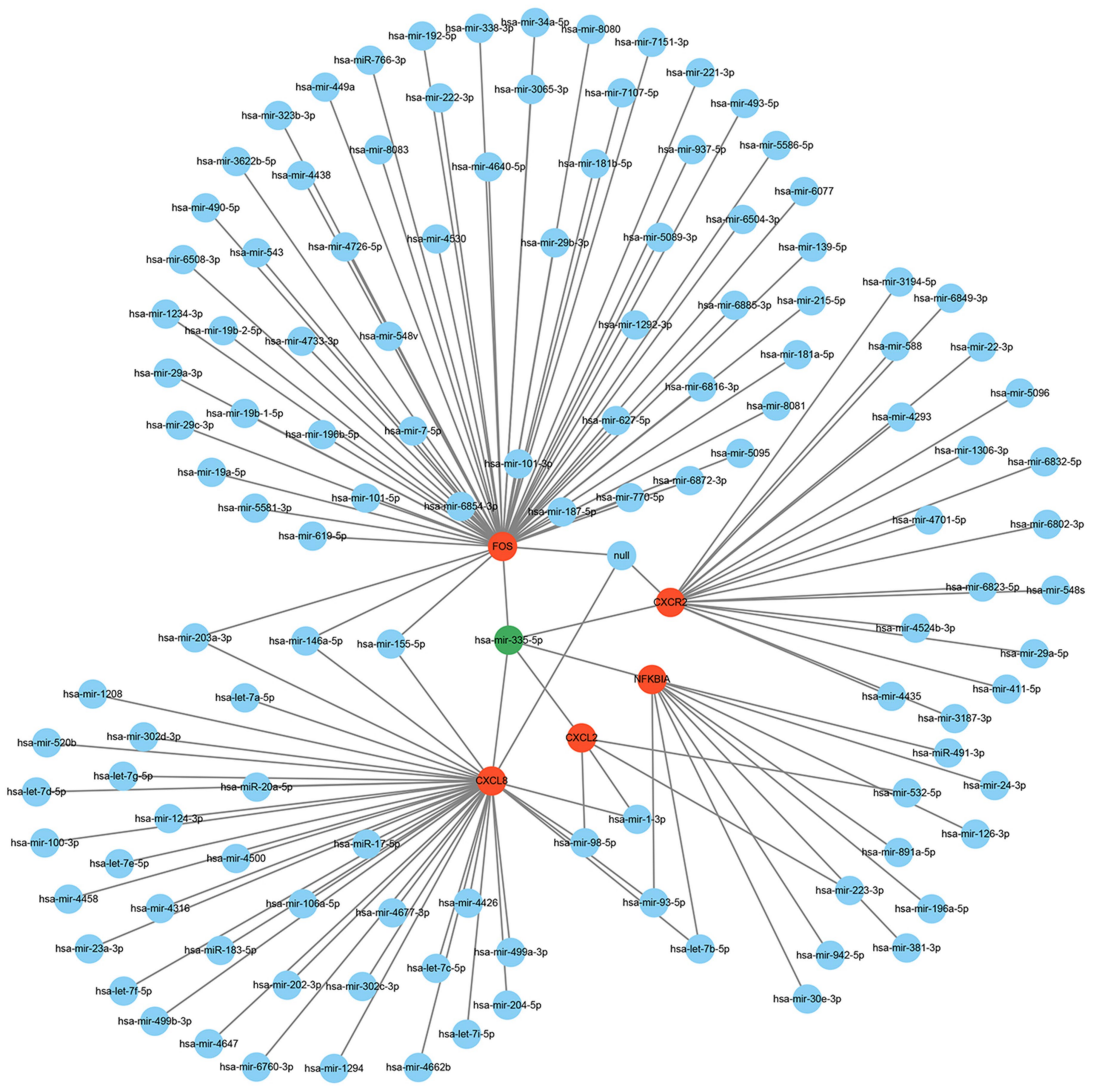
The regulatory network of hub genes and miRNAs.

Many studies have demonstrated that miRNAs perform their biological functions by participating in the regulation of their downstream gene translation. Therefore, we hope to find the key miRNAs that interact with these hub genes by constructing a hub genes-miRNAs network. The results showed that hsa-mir-335-5p is the molecule to which these hub genes are co-connected (Figure 8).

## 4 Discussion

Genome-wide studies have identified a number of susceptibility genes for SLE, but the IRGs for SLE remain largely unknown (23). In this study, we screened 945 DEGs for SLE. Further GO functional analysis of DEGs revealed that they were strongly associated with type I interferon and immune function. These results are consistent with previous studies (24–26). KEGG enrichment showed that DEGs were principally participated in NOD-like receptor signaling pathway and TNF signaling pathway. Inhibition of certain important molecules in the NOD-like receptor signaling pathway and the TNF signaling pathway are effective strategies for the treatment of SLE (27–31). There are many studies on this type of research, which we will not elaborate here. In addition, WGCNA constructed modules that were closely related to SLE and selected the two modules with the strongest correlation to take the intersection with DEGs to get CGs. Then we obtained DE-IRGs in SLE by CGs and IRGs. Three machine learning algorithms were selected for these DE-IRGs to obtain hub genes (*CXCL2*, *CXCL8*, *FOS*, *NFKBIA*, *CXCR2*). These hub genes showed high sensitivity and high specificity for the diagnosis of SLE (AUC > 0.8). We also validated the model by validation cohort and PBMC. Subsequently, we analyzed the large differences in immune cells expression in SLE and NC as well as the possible influence of hub genes on the involvement of multiple immune cells in the pathogenesis of SLE. Therefore, we hypothesized that these five hub genes are important immune-related biomarkers for SLE. Finally, hsa-mir-335-5p was found to be tightly associated with the hub genes.

CXCL2 (C-X-C Motif Chemokine Ligand 2), CXCL8 (C-X-C Motif Chemokine Ligand 8) and CXCR2 (C-X-C Motif Chemokine Receptor 2) are all members of the chemokine family. CXCL2 and CXCR2 are a bunch of important chemokine ligands and receptors. CXCL2 – CXCR2 has been shown to play an important role in the development of a variety of tumors and is closely associated with neutrophil activation and migration (32–35). Neutrophils are the most abundant immune cells in the body and are inextricably linked to the development of SLE. Abnormal activation of neutrophils can exacerbate inflammatory responses and tissue damage. Neutrophils can also promote immune complex formation through the release of cytokines, the generation of neutrophil extracellular traps (NETs), and the production of oxidative stress, which can lead to exacerbation of the autoimmune response, especially in complications such as lupus nephritis (LN) (36–38). In addition, stimulation by autoantibodies promotes ferroptosis of neutrophils thereby exacerbating inflammation (39). It is in accordance with the outcome of our immune cell analysis. CXCL8 is also called interleukin 8 (IL-8), has a similar effect on neutrophils as CXCL2-CXCR2 and also promotes the formation of NETs to exacerbate inflammation and tissue damage (40). CXCL8 levels were significantly elevated in the serum of SLE patients and positively correlated with proteinuria, sedimentation, antinuclear antibodies and SLEDAI. And there is a strong correlation between *IL-8* gene polymorphisms and SLE risk (41). We hypothesized that CXCL2-CXCR2 and CXCL8 may exacerbate SLE organ damage by promoting aberrant activation and migration of immune cells (especially neutrophils).

FOS (FBJ Murine Osteosarcoma Viral Oncogene Homolog) is a class of genes associated with cell proliferation, differentiation and survival and is a member of the transcription factor family. The Activator Protein 1 (AP-1) dimeric structure composed of FOS and JUN is involved in the regulation of many immune responses and inflammatory processes (42). Follicular helper T cell (TFH) numbers expanded and correlated with disease activity in SLE (42, 43). The AP-1 complex promotes antibody production by regulating the proliferation and differentiation of B cells into plasma cells (44). Meanwhile, AP-1 is an important transcription factor in the T-cell activation process, regulates TFH proliferation, and inhibits IL-2 production, promoting SLE progression (45, 46). This suggests that FOS may also be an important immune marker for SLE.

NFKBIA (Nuclear Factor Kappa-B Inhibitor Alpha, also named IKBA) is a potent inhibitor of Nuclear Factor Kappa-B (NF-KB). Over-activation of the NF-KB signaling pathway promotes the expression of TNF-*α*, IL-1β, and IL-6, which exacerbates inflammation and tissue damage (47, 48). These cytokines happen to be the substances involved in the key SLE (49–51). Yang et al. (52) found that inhibition of the NFKB signaling pathway significantly reduced urinary protein and autoantibody levels in lupus mice, as well as reduced renal immune complex deposition. Therefore, inhibiting the over-activation of NFKB by enhancing the expression of NFKBIA may be an effective way to attenuate the inflammatory and immune responses in SLE.

Multiple studies have shown that miRNAs play an essential function in the development of SLE. For example, miR-590-3p ameliorated inflammation in lupus mice by inhibiting Th17 cell differentiation (53). miR-21 and miR-155 genetic variants were associated with susceptibility to SLE (54). Xu et al. (55) found that *IL-10* targeting E2F2-miR-17-5p inhibited autoantibody secretion in active SLE patients. The hsa-mir-335-5p is widely expressed in human and has been found to positively correlate with anti-CCP antibodies and C-reactive protein in rheumatoid arthritis (RA), and is a good biomarker for RA (56). It is also a valid marker for osteoarthritis (57). Inhibition of *FOS* expression by has-mir-335-3p regulates bone metabolic homeostasis in a stress mouse model. However, there is a lack of reports on the direct link between hub genes, microRNA and SLE.

Notably, the samples we chose for our dataset were all from peripheral blood, and we also validated this by peripheral blood from SLE patients, which may greatly support our results. Nevertheless, there are some shortcomings in our study. First, the dataset we analyze is an online public dataset, which is a secondary mining of the data. Second, the small sample size and the sample originating from one center in this study may be biased. Third, our immune cell analysis could not directly assess the limitations of tissue-resident immune cells, and it is hoped that future studies may combine tissue sampling with circulating cell analysis to gain a more comprehensive understanding. Fourth, due to the long duration of the disease in SLE patients and the wide range of medications used during treatment, the effect of medications on pivotal gene expression cannot be excluded. Finally, we lack more experiments to verify our results. Therefore, in the future, we will do further research in *in vivo* or *in vitro* experiments.

## 5 Conclusion

In summary, this is a study to screen for IRGs and metabolic pathways that are hubs in the peripheral blood of SLE. We identified five hub genes (*CXCL2*, *CXCL8*, *FOS*, *NFKBIA* and *CXCR2*), and constructed and validated a diagnostic model. We hope to provide new directions and evidence for the pathogenesis and treatment of SLE.

## Data Availability

The datasets presented in this study can be found in online repositories. The names of the repository/repositories and accession number(s) can be found in the article/Supplementary material.

## Footnotes

^1^ https://www.ncbi.nlm.nih.gov/geo/

^2^ https://cn.string-db.org/

^3^ https://www.immport.org/shared

^4^ https://cibersortx.stanford.edu/

^5^ https://www.networkanalyst.ca/NetworkAnalyst/
