# Supplementary figures and images for: Identification of hub immune-related genes and construction of predictive models for systemic lupus erythematosus by bioinformatics combined with machine learning
Source: Front Med (Lausanne). 2025 May 14;12:1557307. doi: 10.3389/fmed.2025.1557307 (PMC12116674; doi:10.3389/fmed.2025.1557307)

# Before Normalization

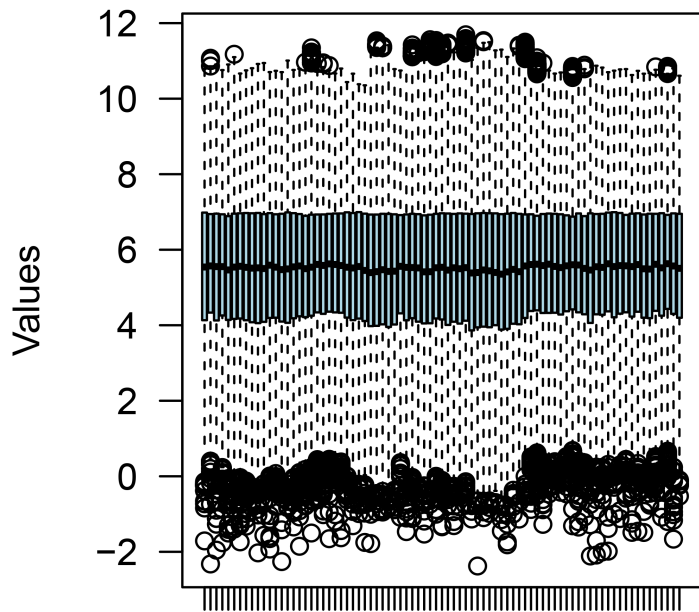

# After Normalization

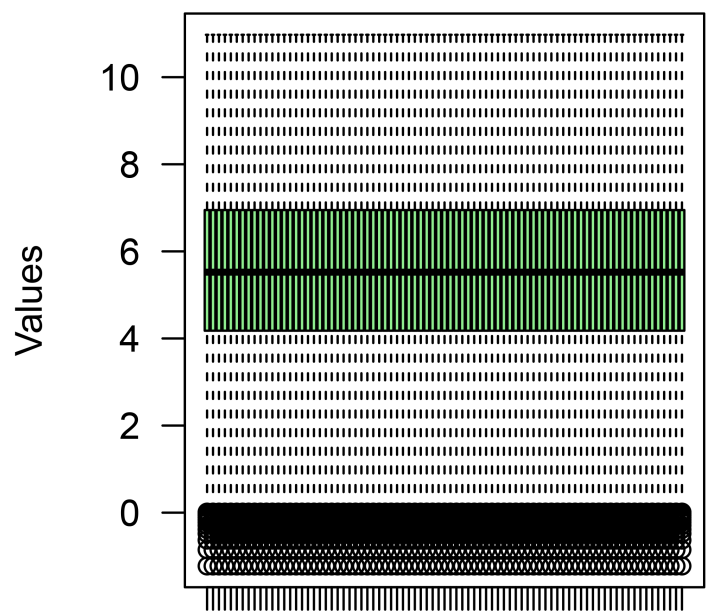

Supplement: Supplementary file 1 [file Data_Sheet_1.PDF]

# NOD-LIKE RECEPTOR SIGNALING PATHWAY

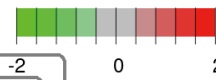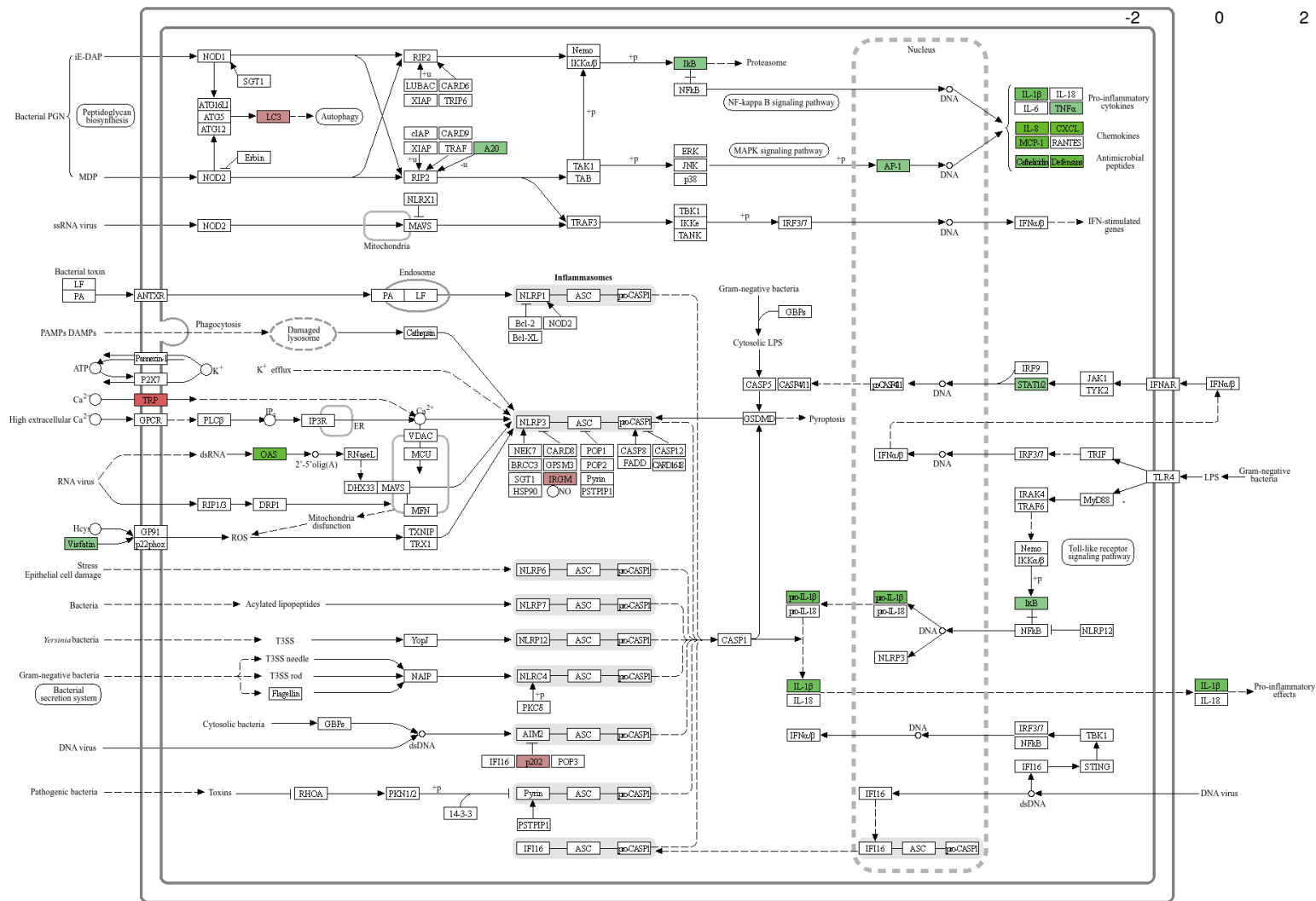

Data on KEGG graph  
Rendered by Pathway

Supplement: Supplementary file 2 [file Data_Sheet_2.PDF]

**A**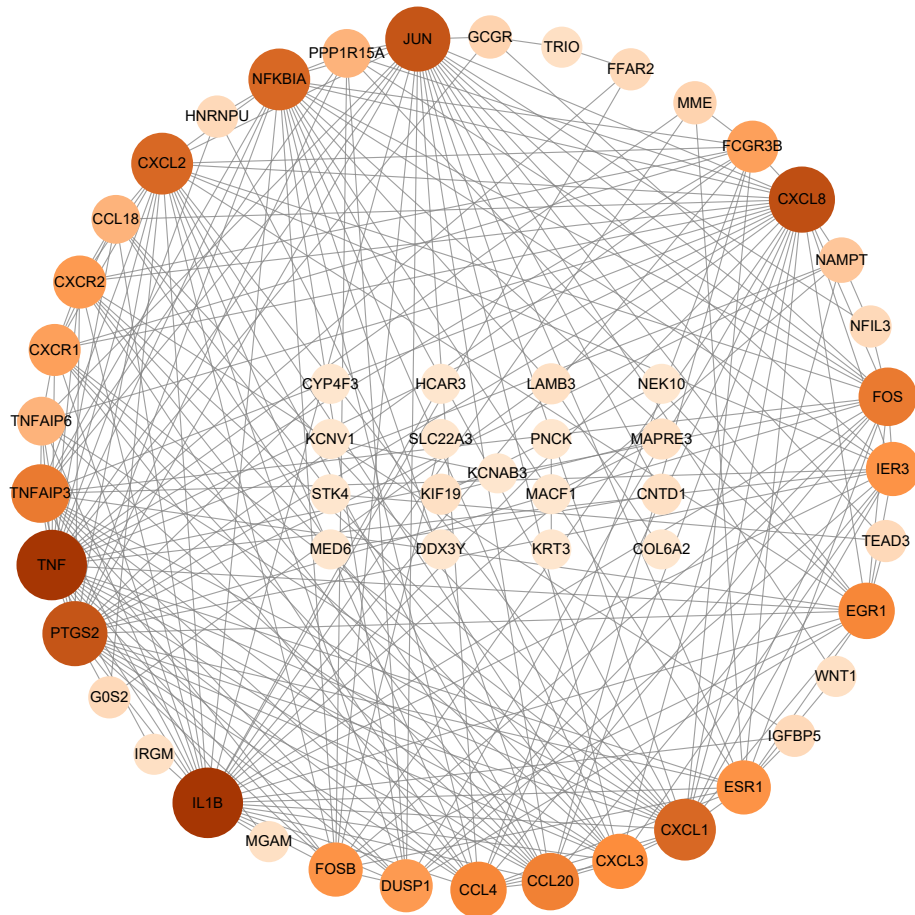**B**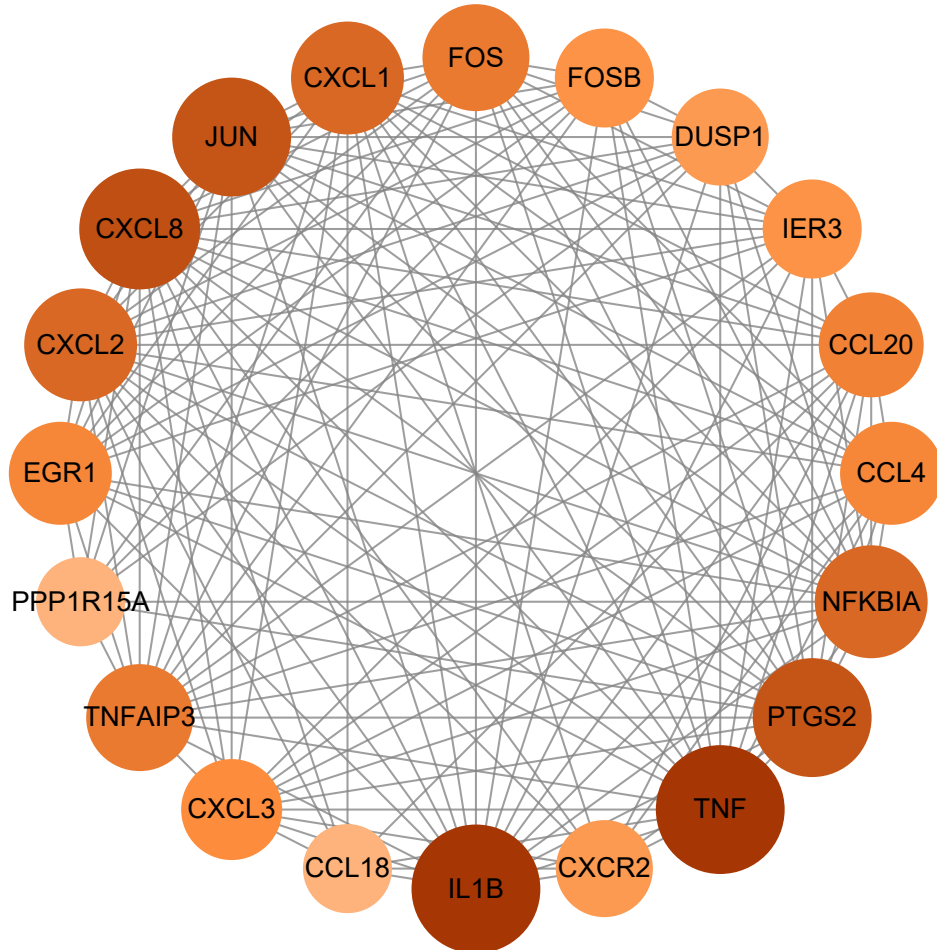

Supplement: Supplementary file 3 [file Data_Sheet_3.PDF]

**A**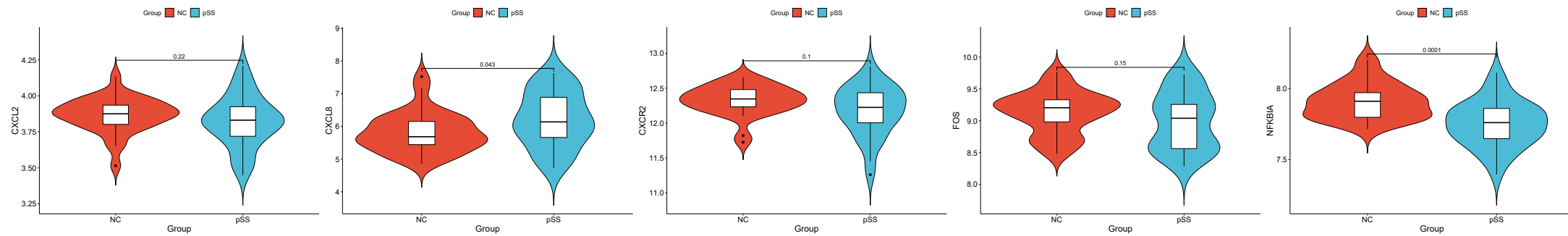**B**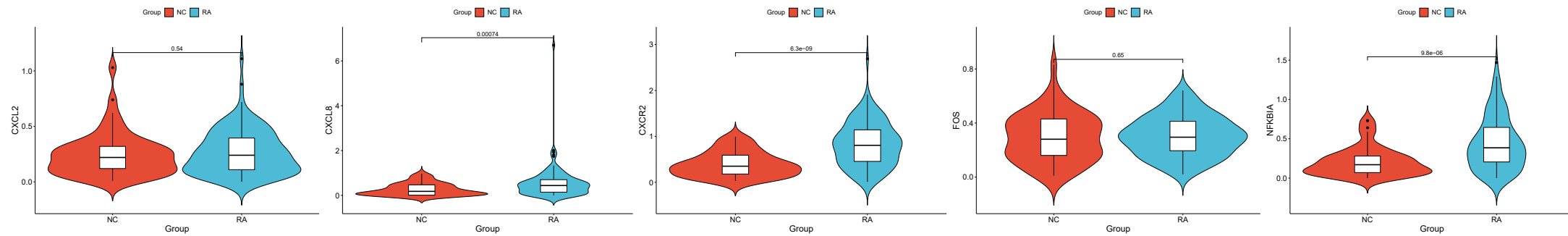**C****GSE84844 (pSS)**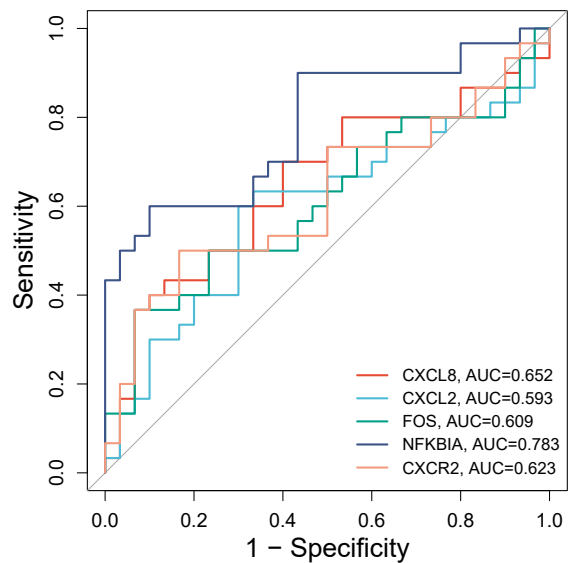**D****GSE17755 (RA)**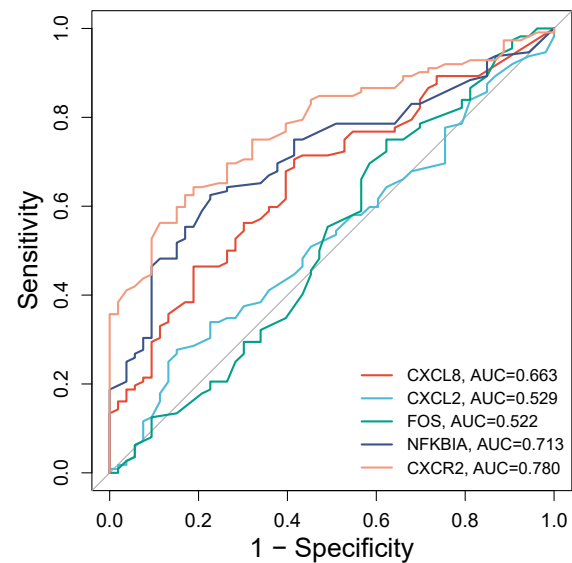

Supplement: Supplementary file 4 [file Data_Sheet_4.PDF]

### Before Normalization

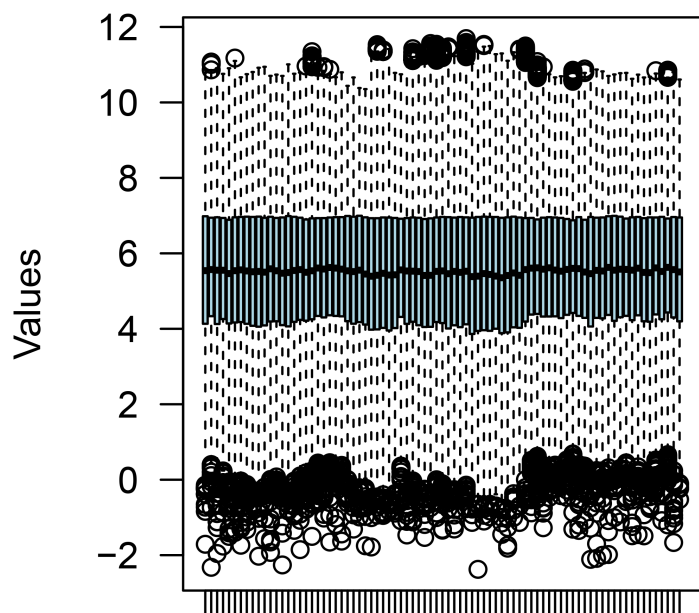

### After Normalization

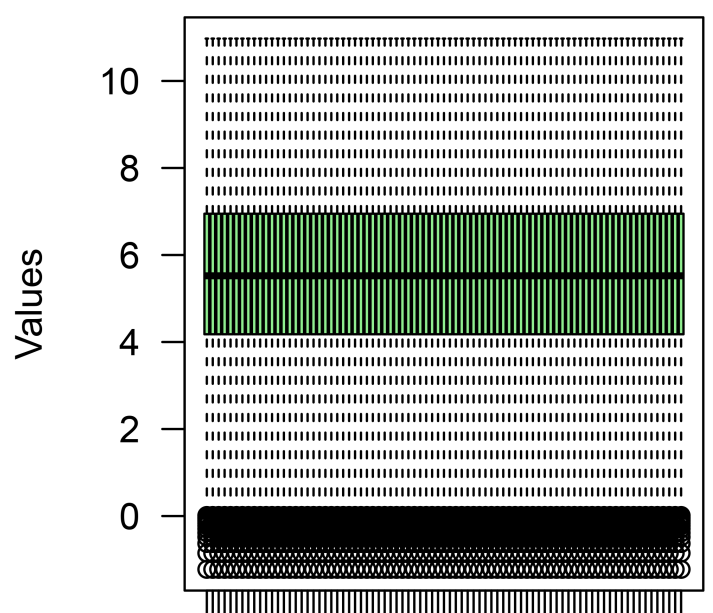

Supplement: Supplementary file 5 [file Data_Sheet_5.ZIP › Raw data/image files/Before and after normalization of the dataset GS50772.pdf]

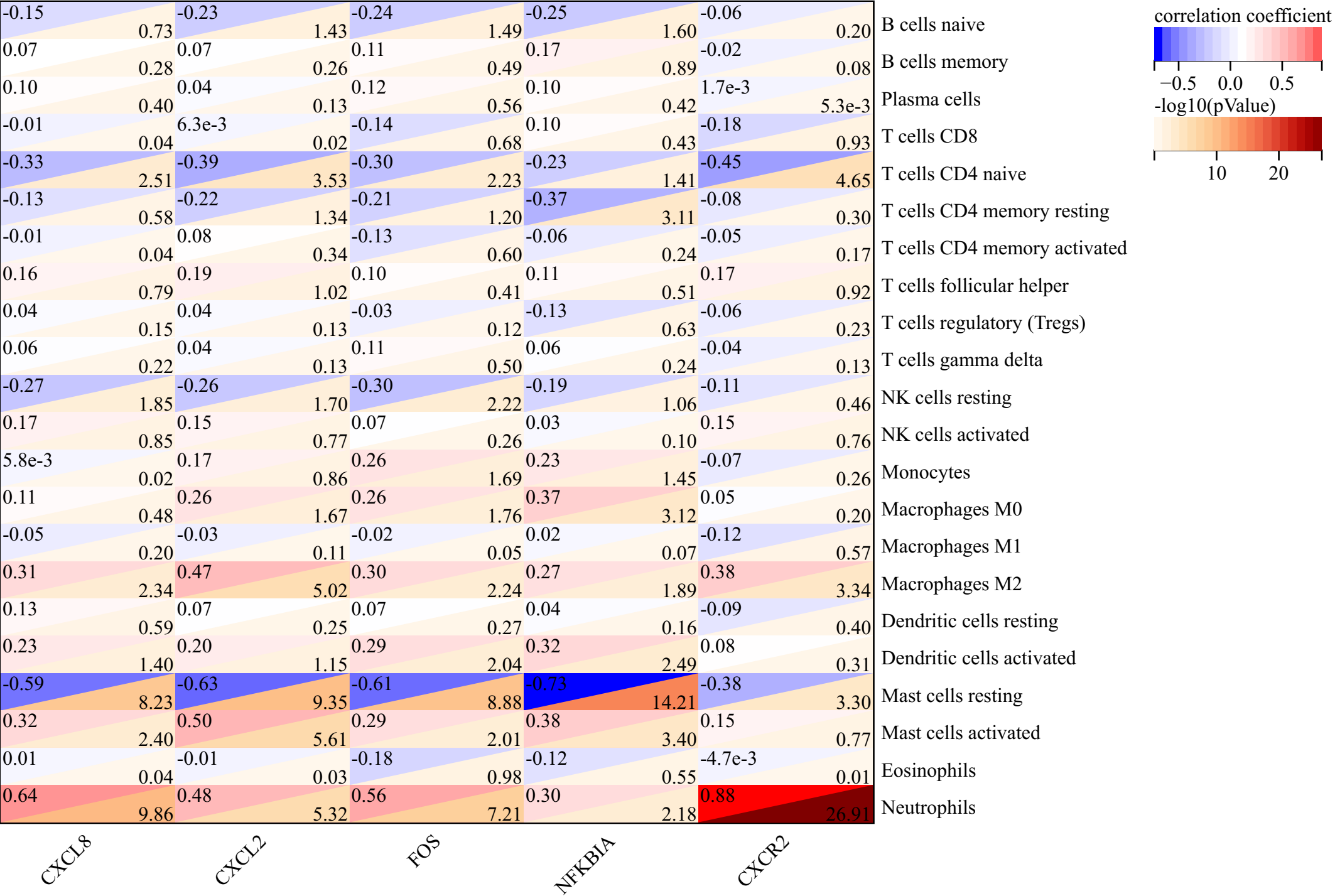

Supplement: Supplementary file 5 [file Data_Sheet_5.ZIP › Raw data/image files/corrlation analysis.pdf]

Group 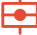 NC 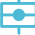 SLE

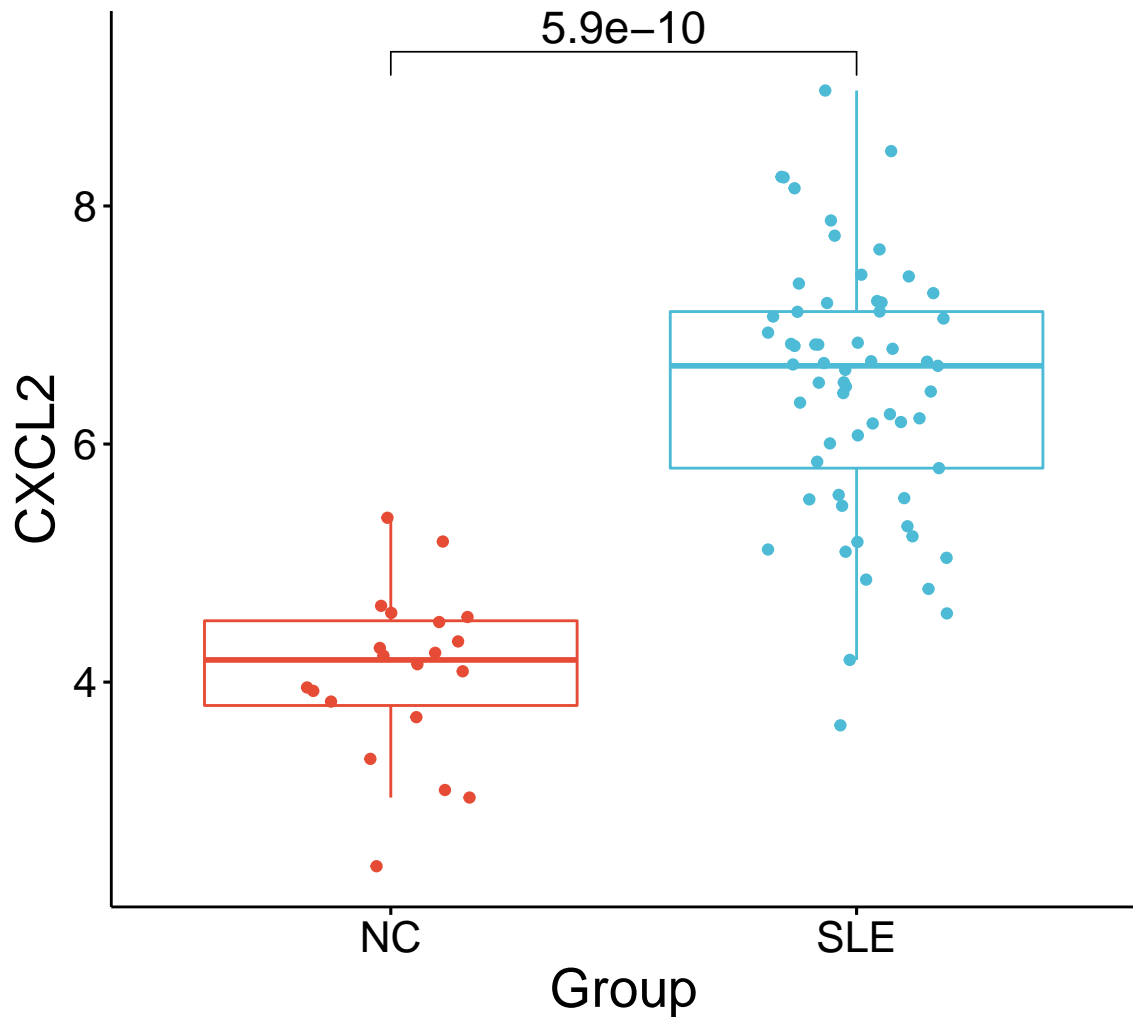

Supplement: Supplementary file 5 [file Data_Sheet_5.ZIP › Raw data/image files/CXCL2 expression of training dataset.pdf]

Group 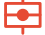 NC 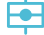 SLE

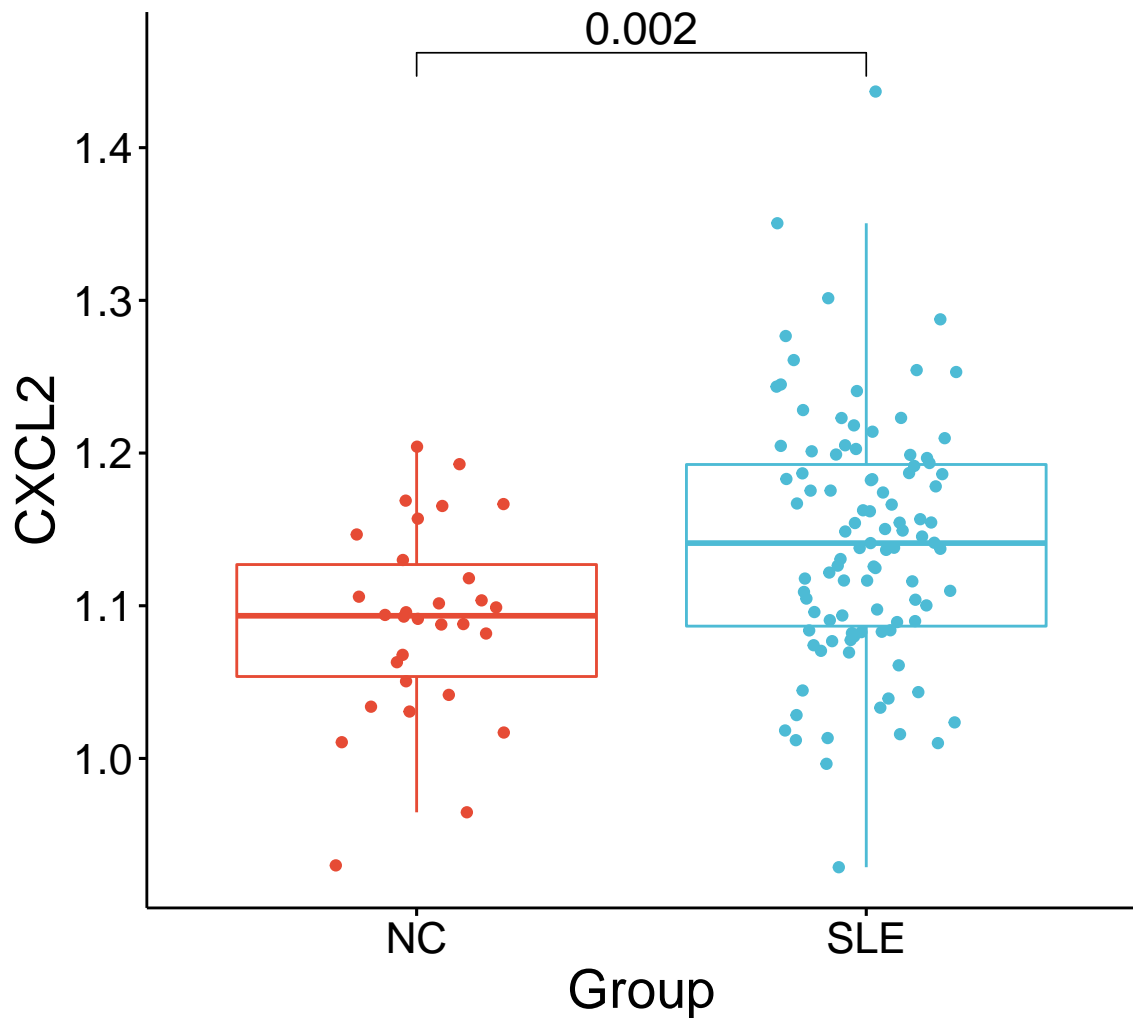

Supplement: Supplementary file 5 [file Data_Sheet_5.ZIP › Raw data/image files/CXCL2 expression of validating dataset.pdf]

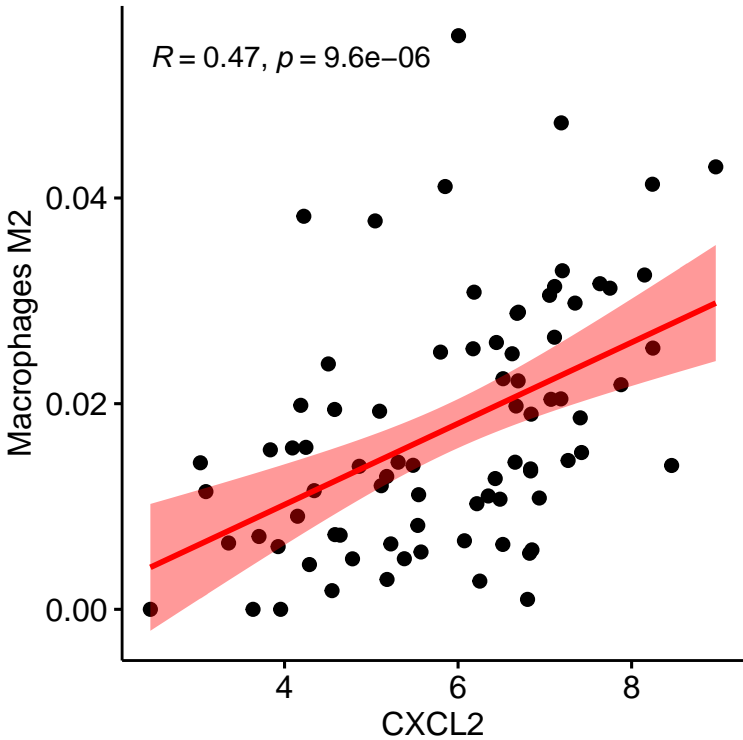

Supplement: Supplementary file 5 [file Data_Sheet_5.ZIP › Raw data/image files/CXCL2-M2 macrophage.pdf]

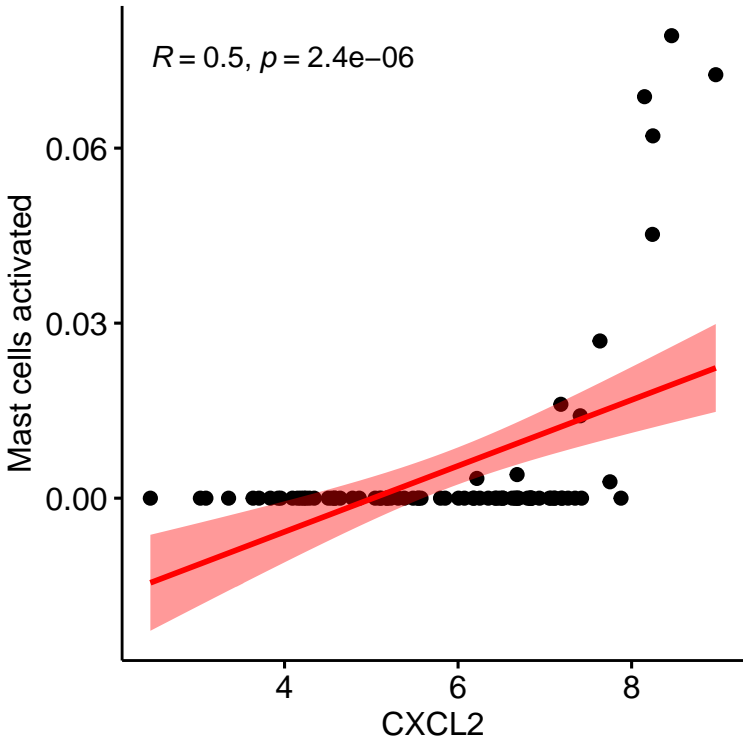

Supplement: Supplementary file 5 [file Data_Sheet_5.ZIP › Raw data/image files/CXCL2-mast cell actived.pdf]

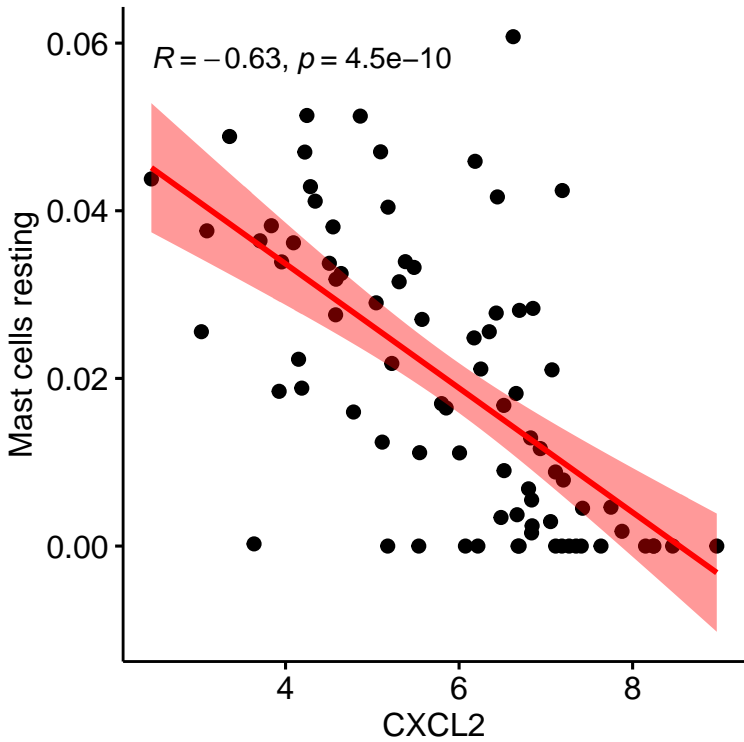

Supplement: Supplementary file 5 [file Data_Sheet_5.ZIP › Raw data/image files/CXCL2-mast cell resting.pdf]

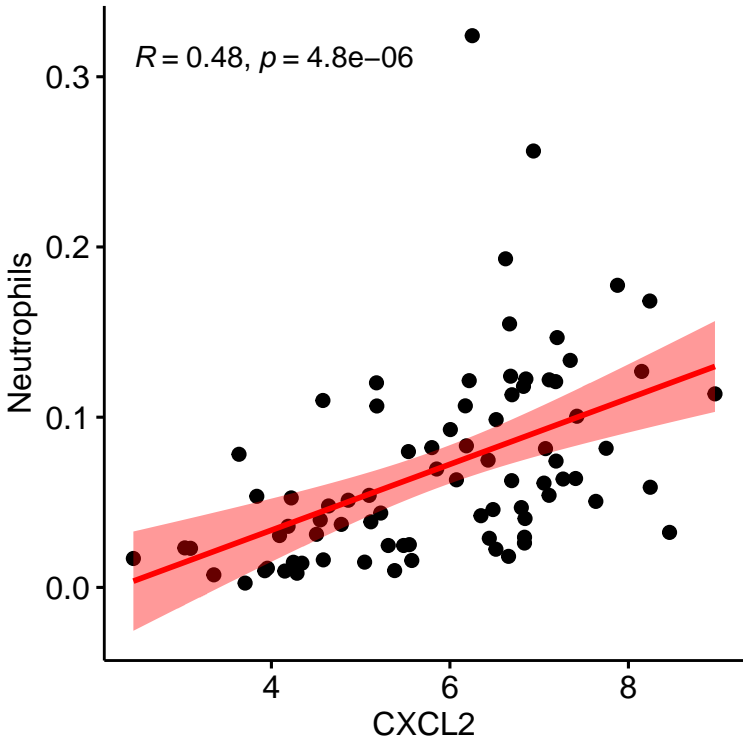

Supplement: Supplementary file 5 [file Data_Sheet_5.ZIP › Raw data/image files/CXCL2-neutrophil.pdf]

# CXCL2

\*\*\*\*

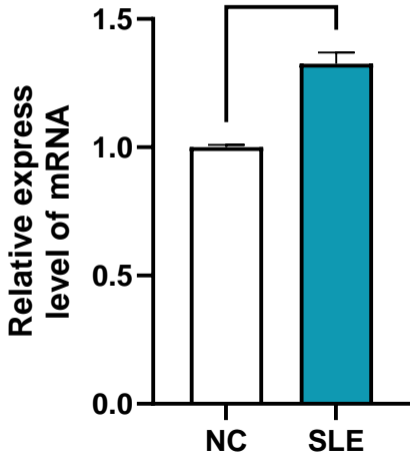

Supplement: Supplementary file 5 [file Data_Sheet_5.ZIP › Raw data/image files/CXCL2-pcr.pdf]

Group 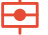 NC 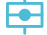 SLE

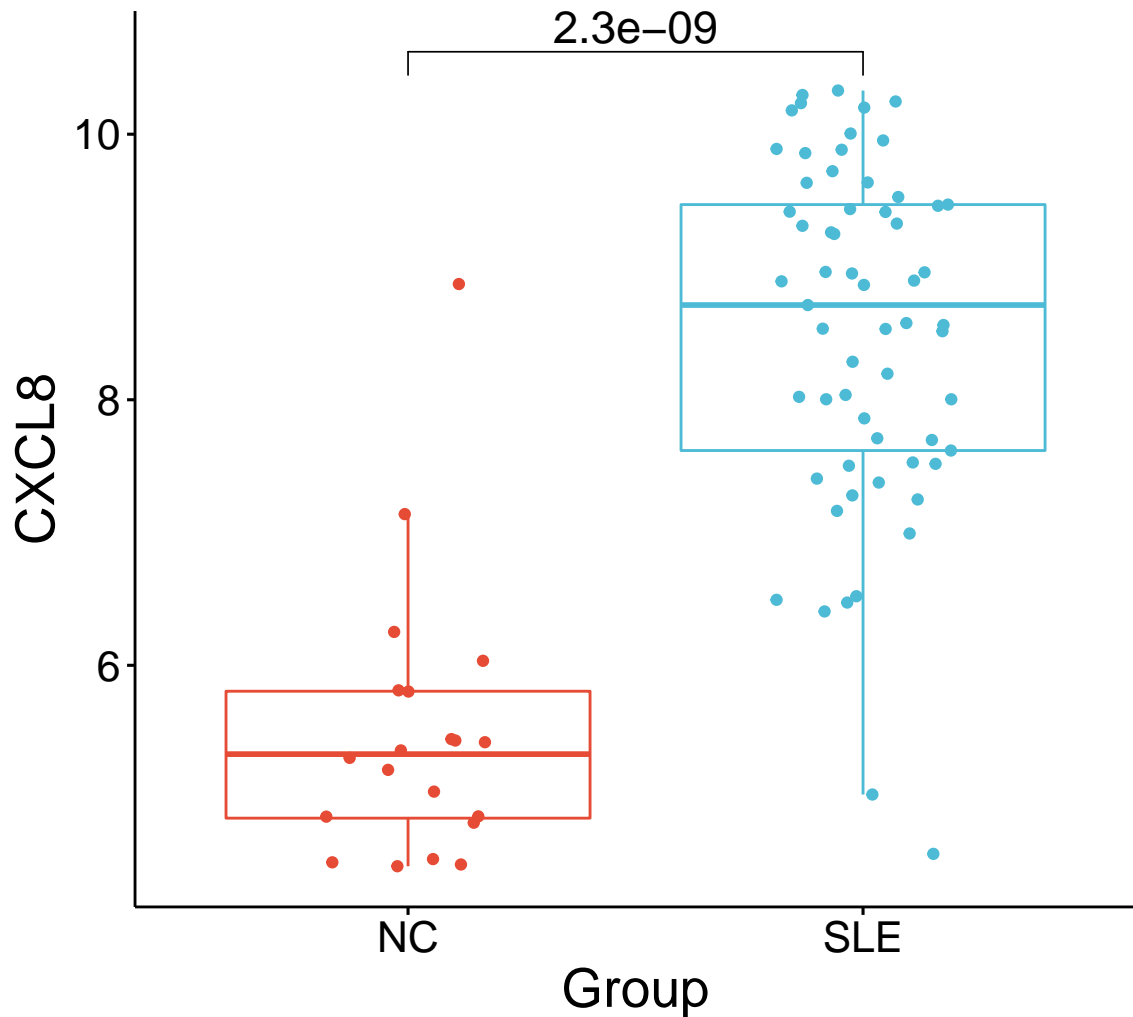

Supplement: Supplementary file 5 [file Data_Sheet_5.ZIP › Raw data/image files/CXCL8 expression of training dataset.pdf]

Group 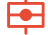 NC 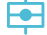 SLE

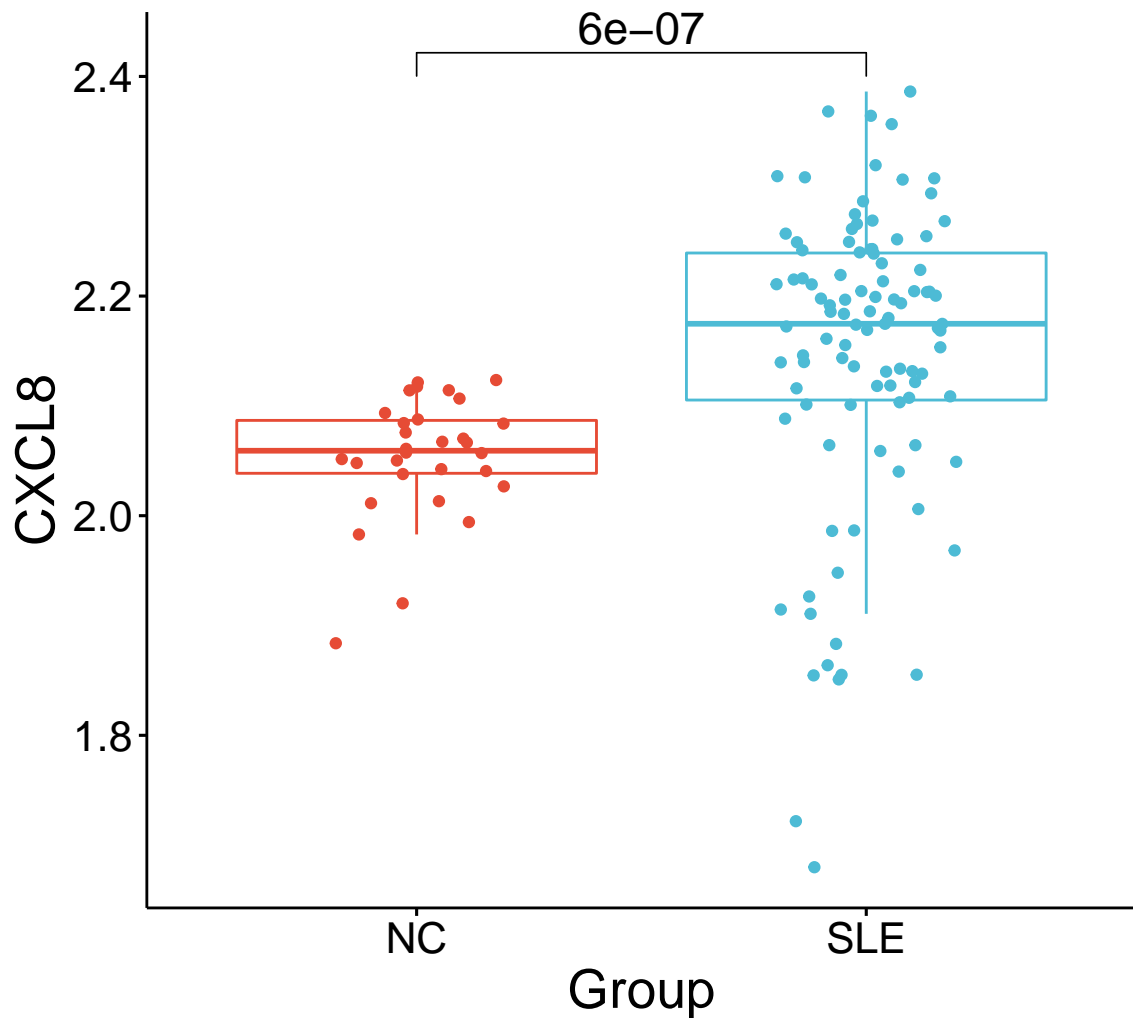

Supplement: Supplementary file 5 [file Data_Sheet_5.ZIP › Raw data/image files/CXCL8 expression of validating dataset.pdf]

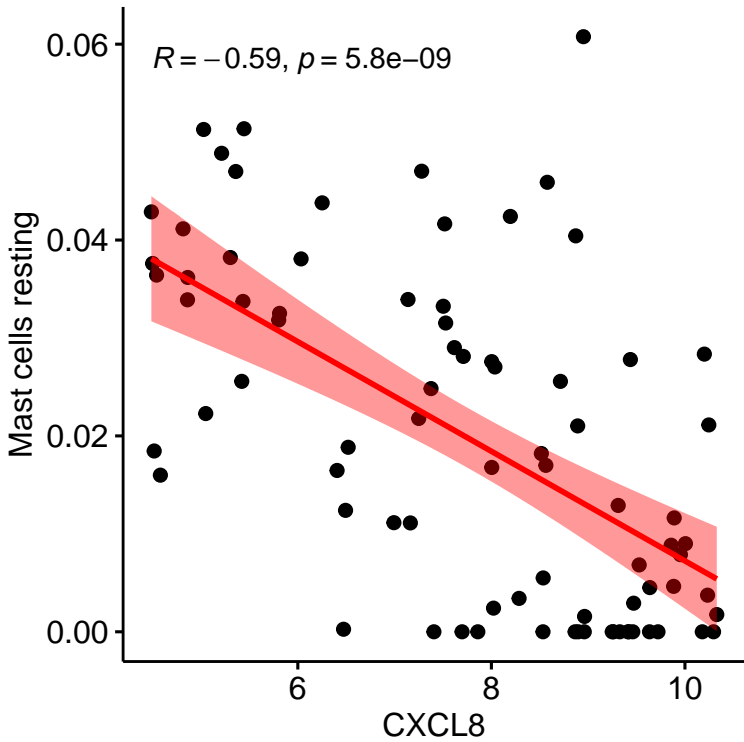

Supplement: Supplementary file 5 [file Data_Sheet_5.ZIP › Raw data/image files/CXCL8-mast cell resting.pdf]

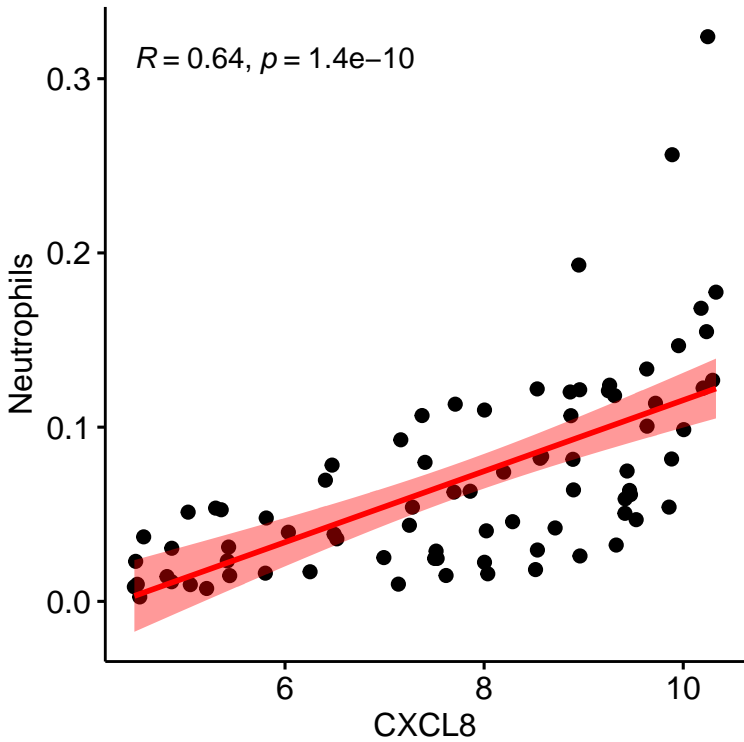

Supplement: Supplementary file 5 [file Data_Sheet_5.ZIP › Raw data/image files/CXCL8-neutrophil.pdf]

# CXCL8

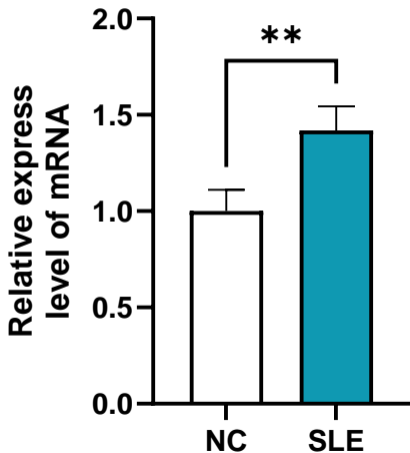

Supplement: Supplementary file 5 [file Data_Sheet_5.ZIP › Raw data/image files/CXCL8-pxr.pdf]

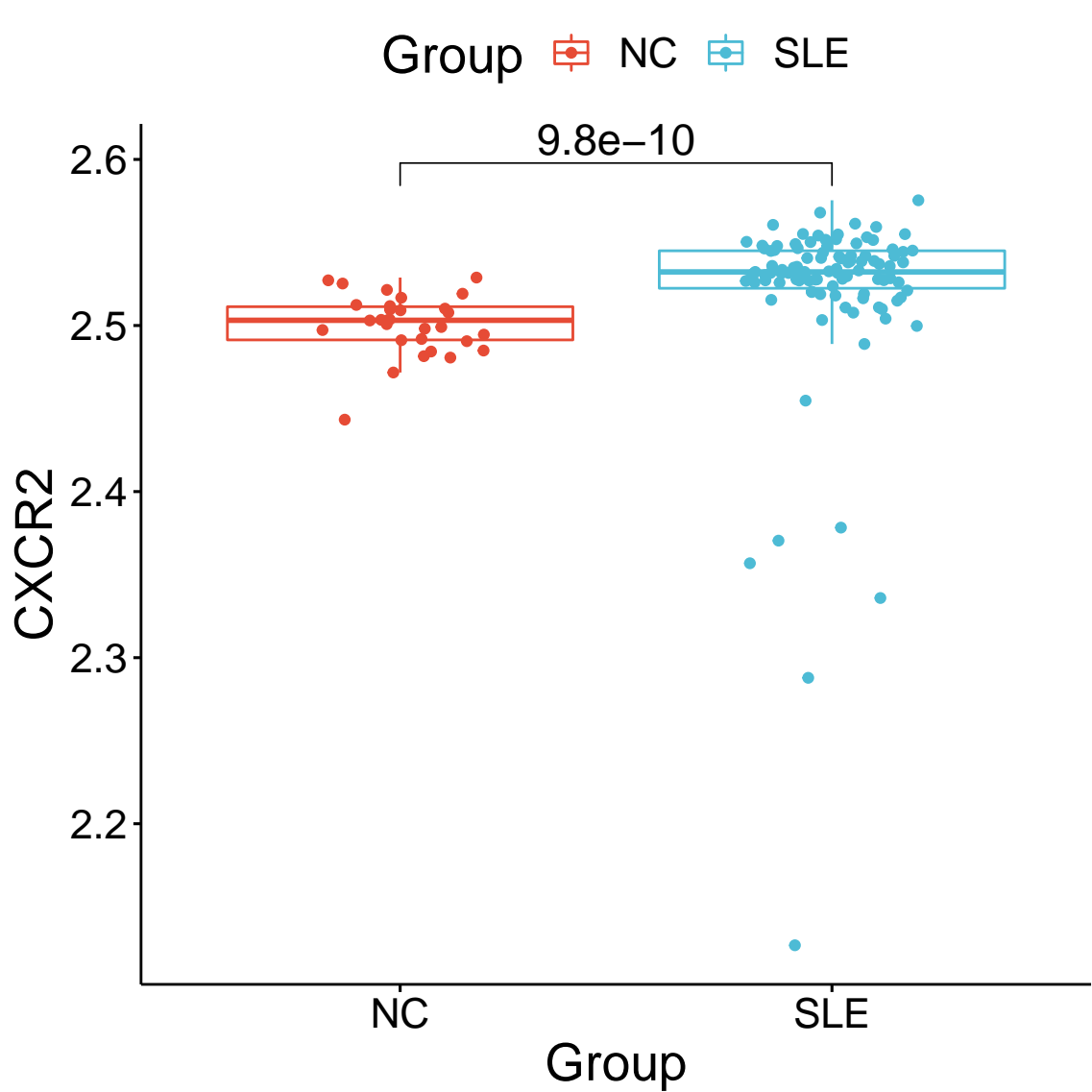

Supplement: Supplementary file 5 [file Data_Sheet_5.ZIP › Raw data/image files/CXCR2 expression of validating dataset.pdf]

Group 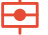 NC 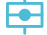 SLE

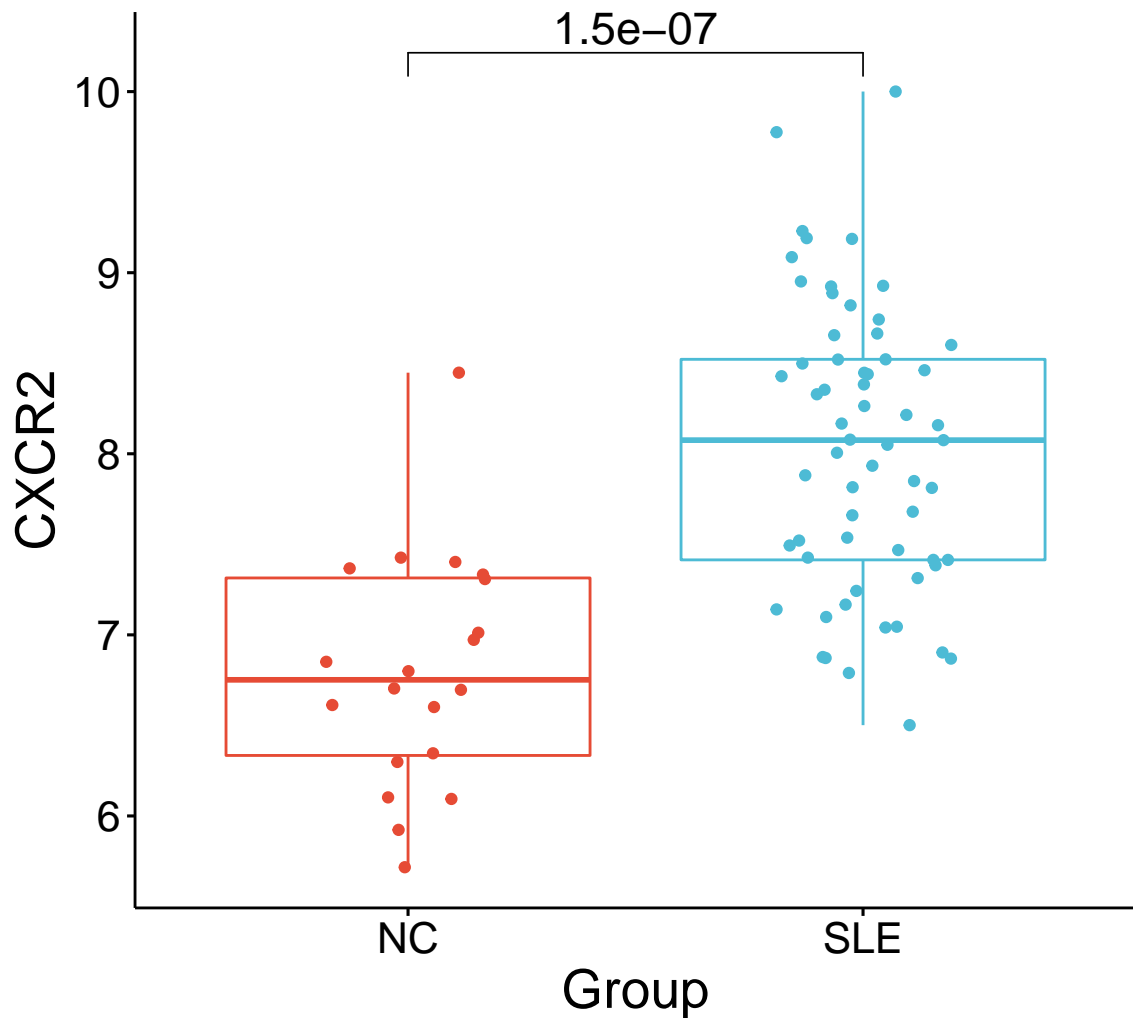

Supplement: Supplementary file 5 [file Data_Sheet_5.ZIP › Raw data/image files/CXCR2 expression of training dataset.pdf]

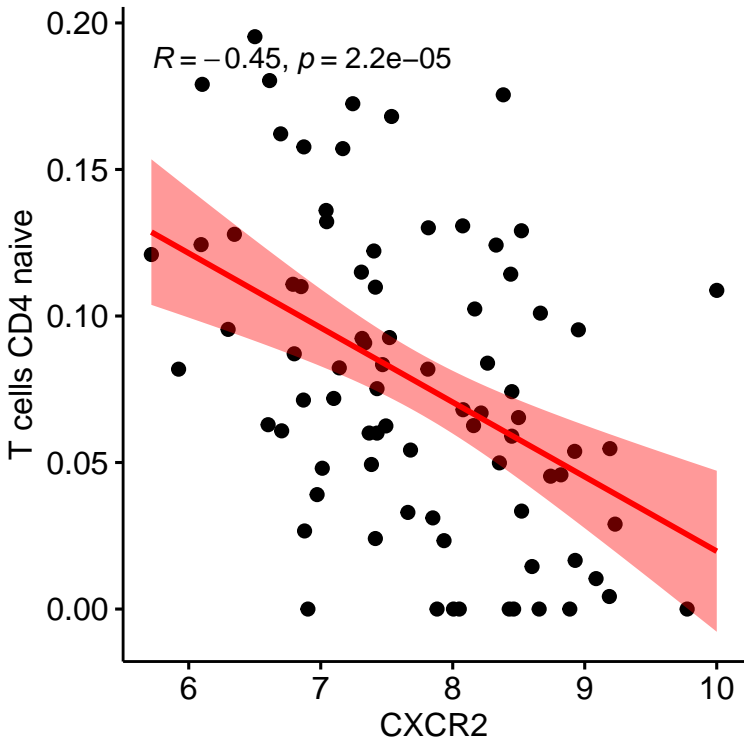

Supplement: Supplementary file 5 [file Data_Sheet_5.ZIP › Raw data/image files/CXCR2-CD4T naive.pdf]

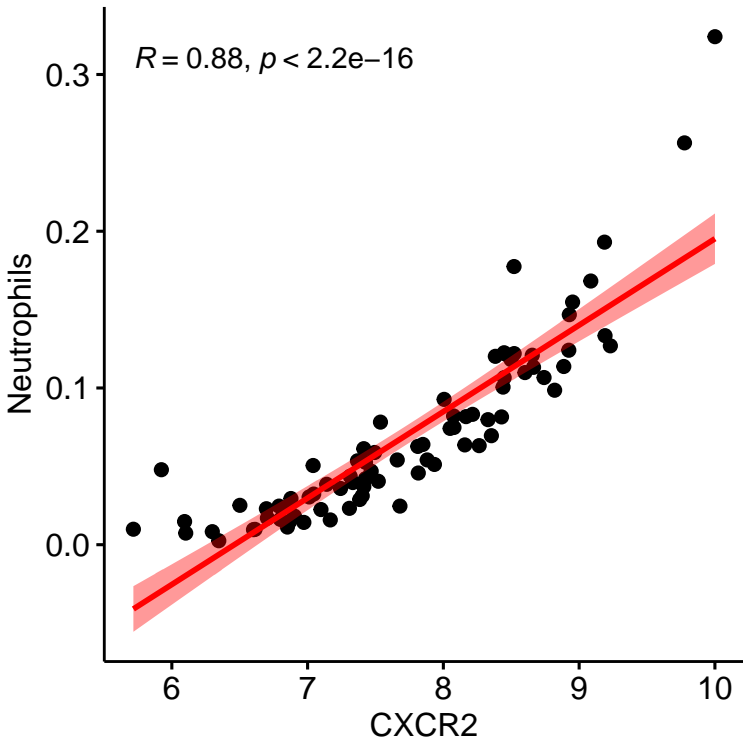

Supplement: Supplementary file 5 [file Data_Sheet_5.ZIP › Raw data/image files/CXCR2-neutrophil.pdf]

# CXCR2

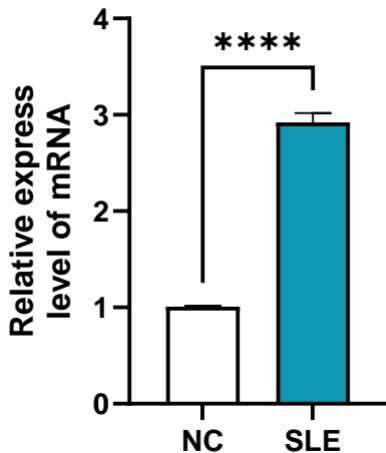

Supplement: Supplementary file 5 [file Data_Sheet_5.ZIP › Raw data/image files/CXCR2-pcr.pdf]

Group 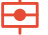 NC 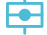 SLE

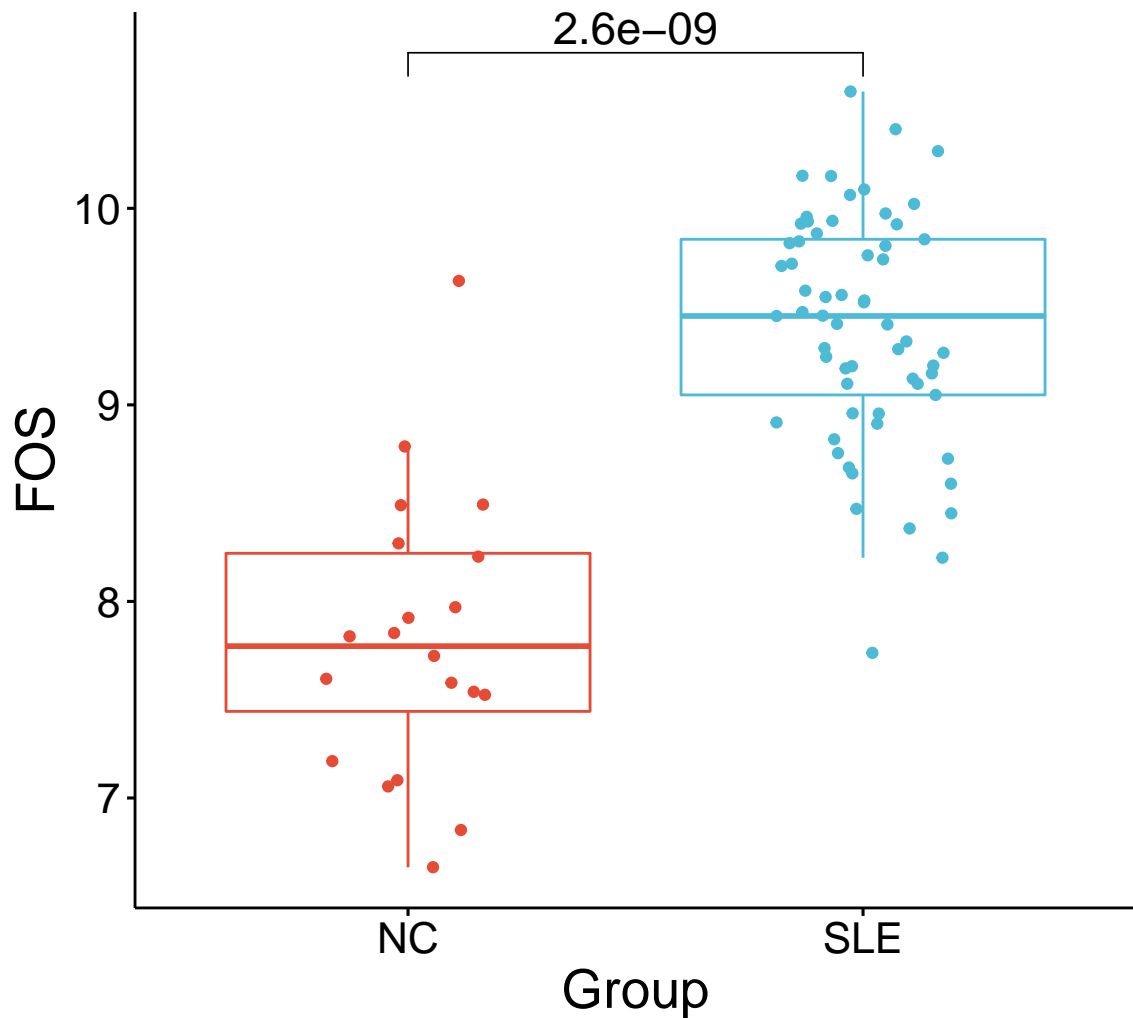

Supplement: Supplementary file 5 [file Data_Sheet_5.ZIP › Raw data/image files/FOS expression of training dataset.pdf]

Group 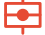 NC 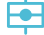 SLE

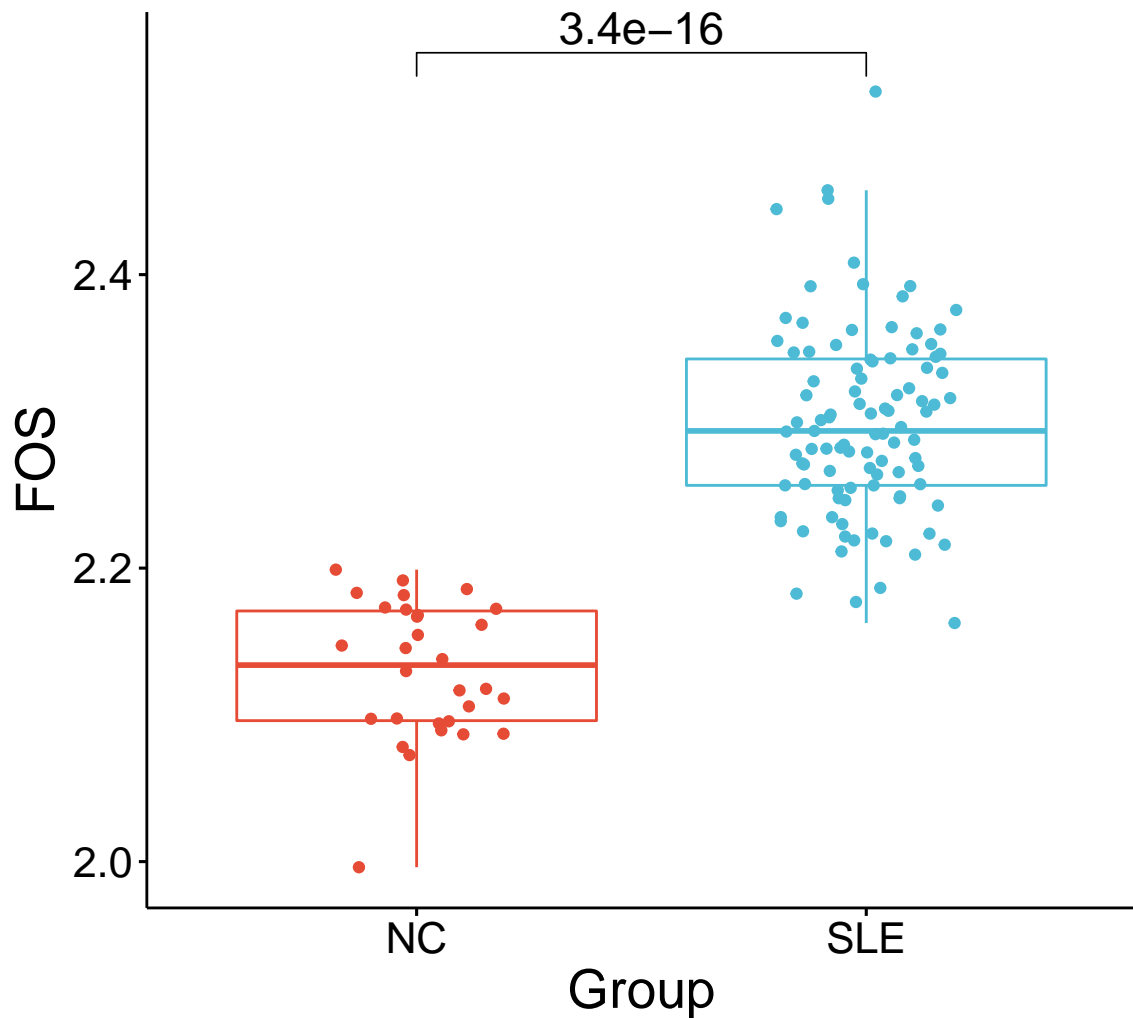

Supplement: Supplementary file 5 [file Data_Sheet_5.ZIP › Raw data/image files/FOS expression of validating dataset.pdf]

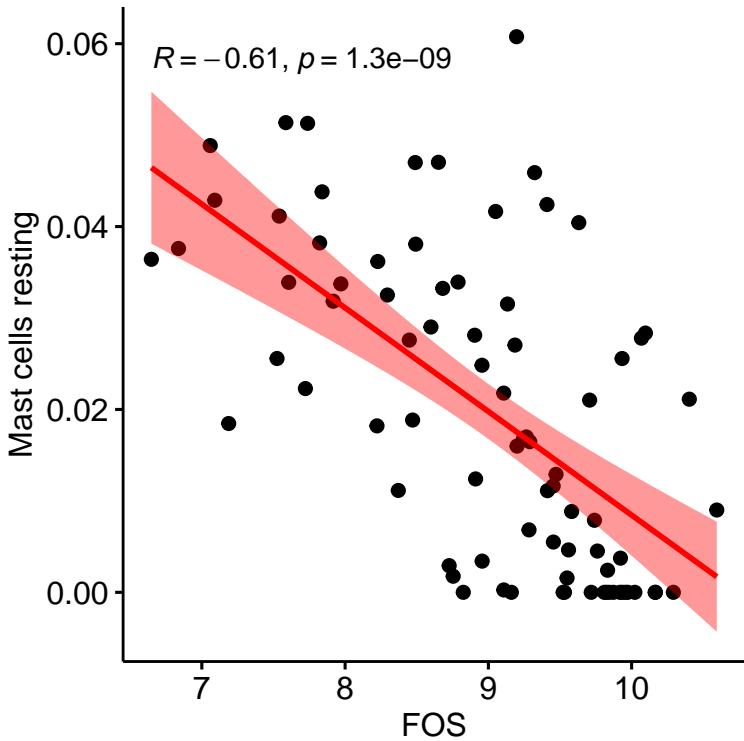

Supplement: Supplementary file 5 [file Data_Sheet_5.ZIP › Raw data/image files/FOS-mast cell resting.pdf]

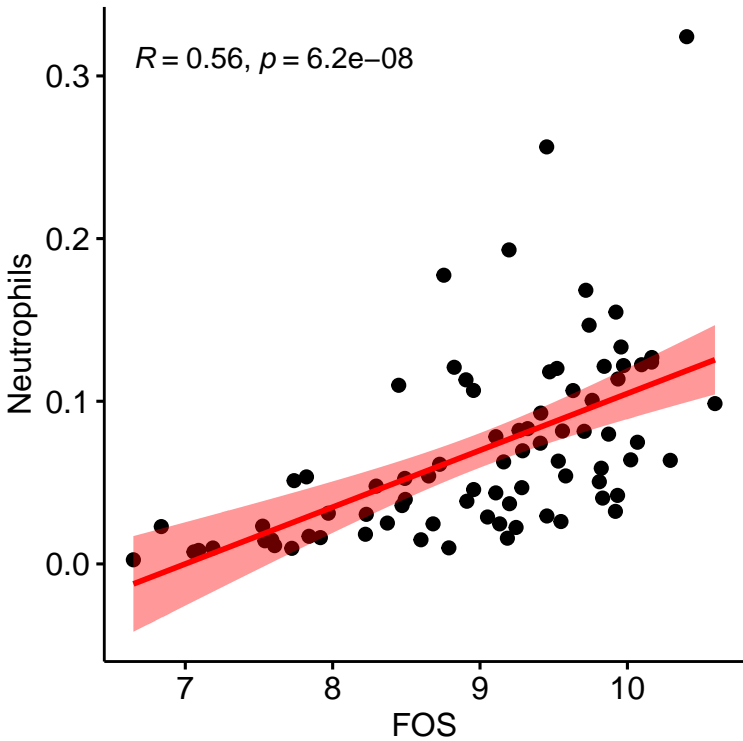

Supplement: Supplementary file 5 [file Data_Sheet_5.ZIP › Raw data/image files/FOS-neutrophil.pdf]

# FOS

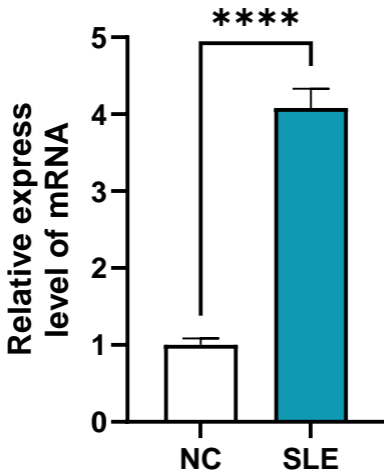

Supplement: Supplementary file 5 [file Data_Sheet_5.ZIP › Raw data/image files/FOS-pcr.pdf]

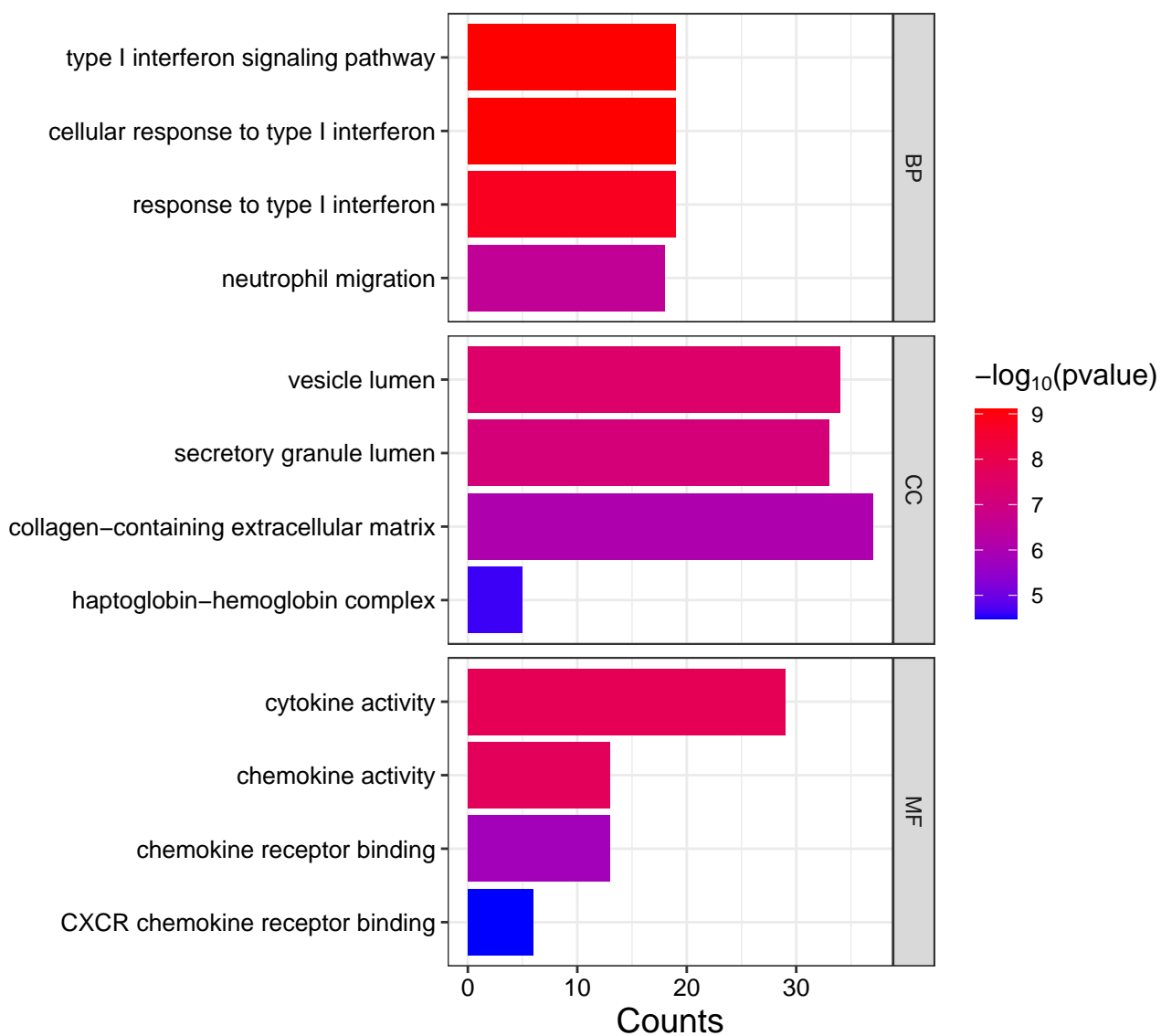

Supplement: Supplementary file 5 [file Data_Sheet_5.ZIP › Raw data/image files/GO_bar.pdf]

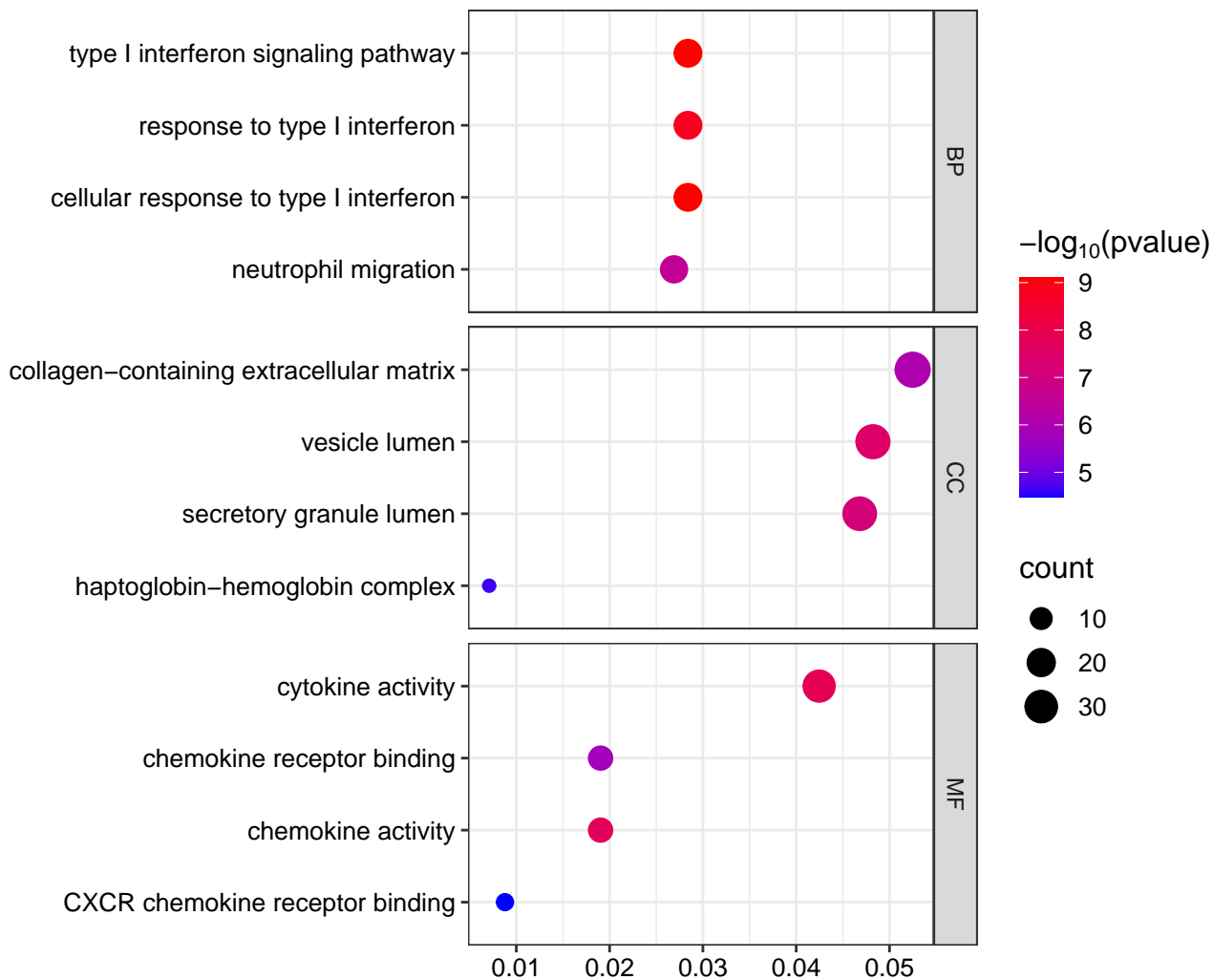

Supplement: Supplementary file 5 [file Data_Sheet_5.ZIP › Raw data/image files/GO_bubble.pdf]

Group:  
● NC  
● SLE

Expression

0.0 0.2 0.4

Group

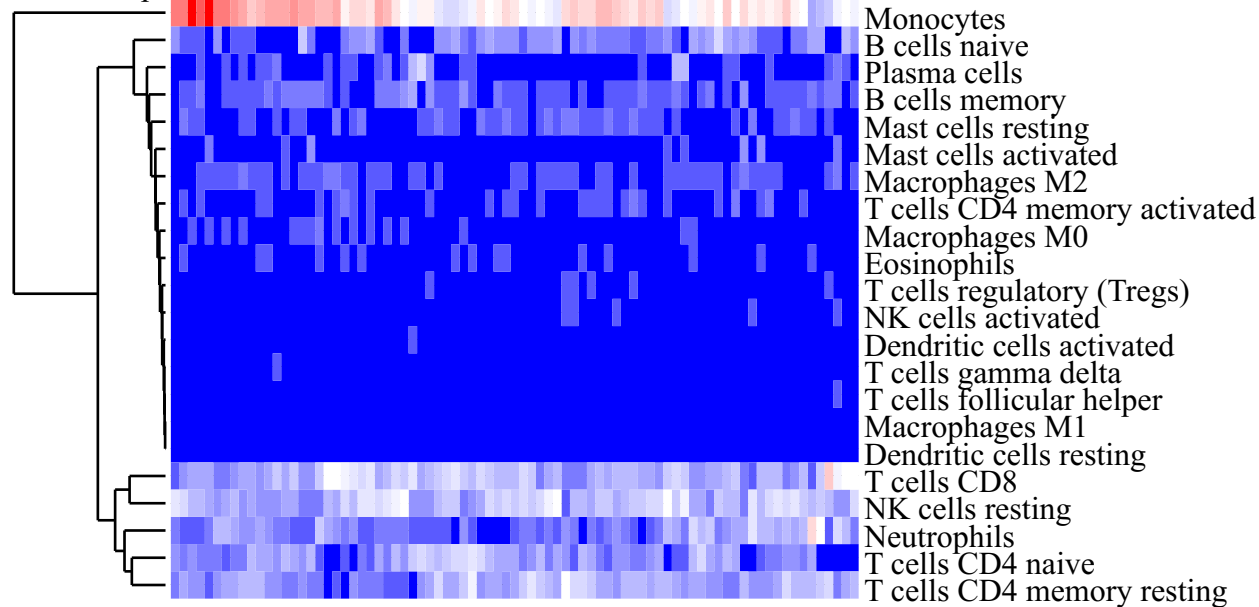

Supplement: Supplementary file 5 [file Data_Sheet_5.ZIP › Raw data/image files/heatmap of immune infiltration.pdf]

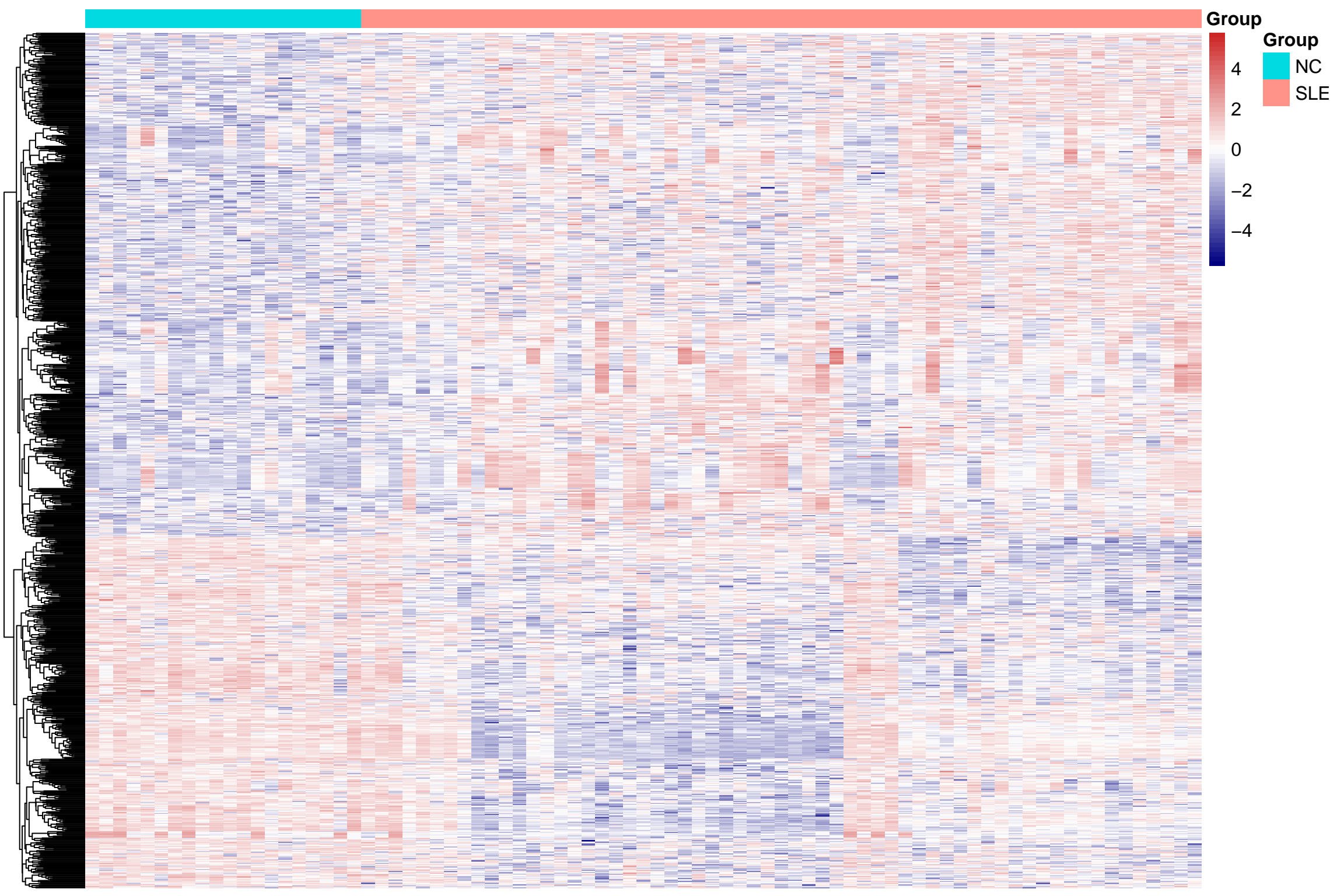

Supplement: Supplementary file 5 [file Data_Sheet_5.ZIP › Raw data/image files/heatmap.pdf]

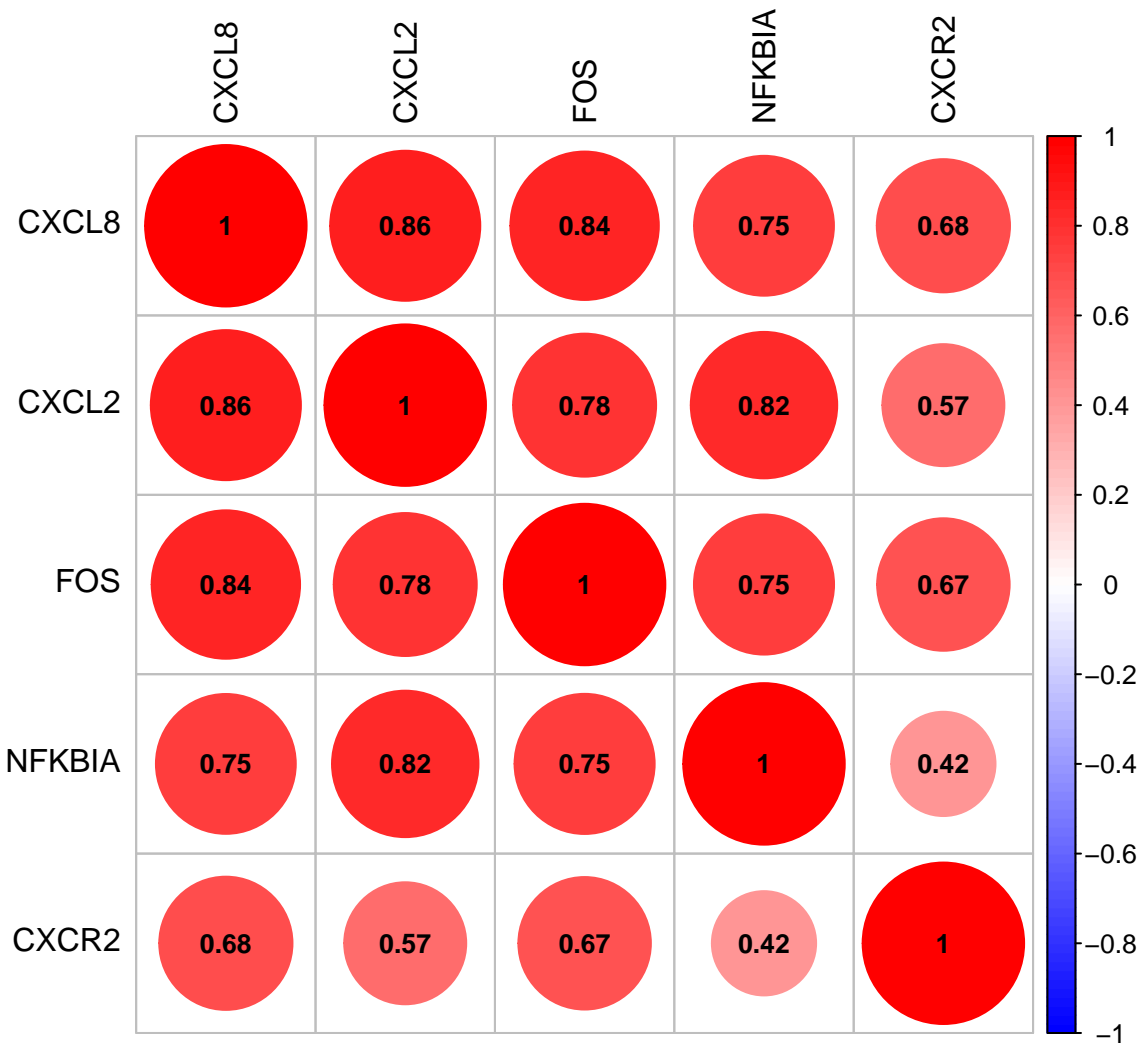

Supplement: Supplementary file 5 [file Data_Sheet_5.ZIP › Raw data/image files/hub corrletaion.pdf]

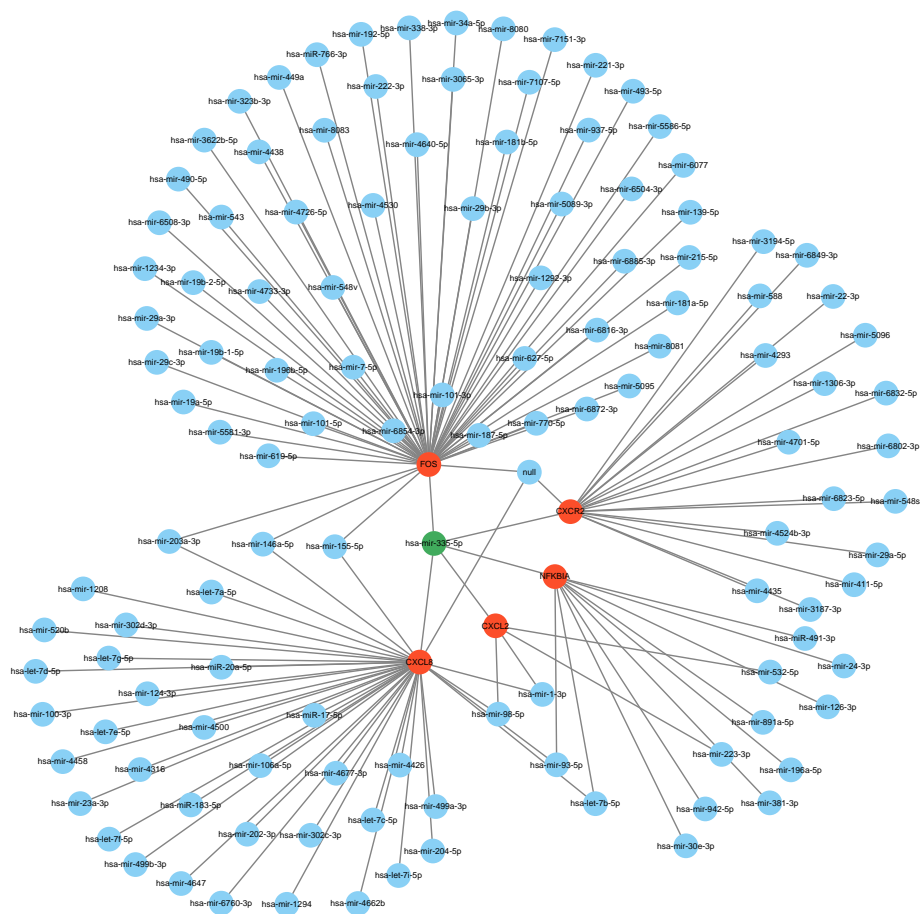

Supplement: Supplementary file 5 [file Data_Sheet_5.ZIP › Raw data/image files/Hub genes and miRNA.pdf]

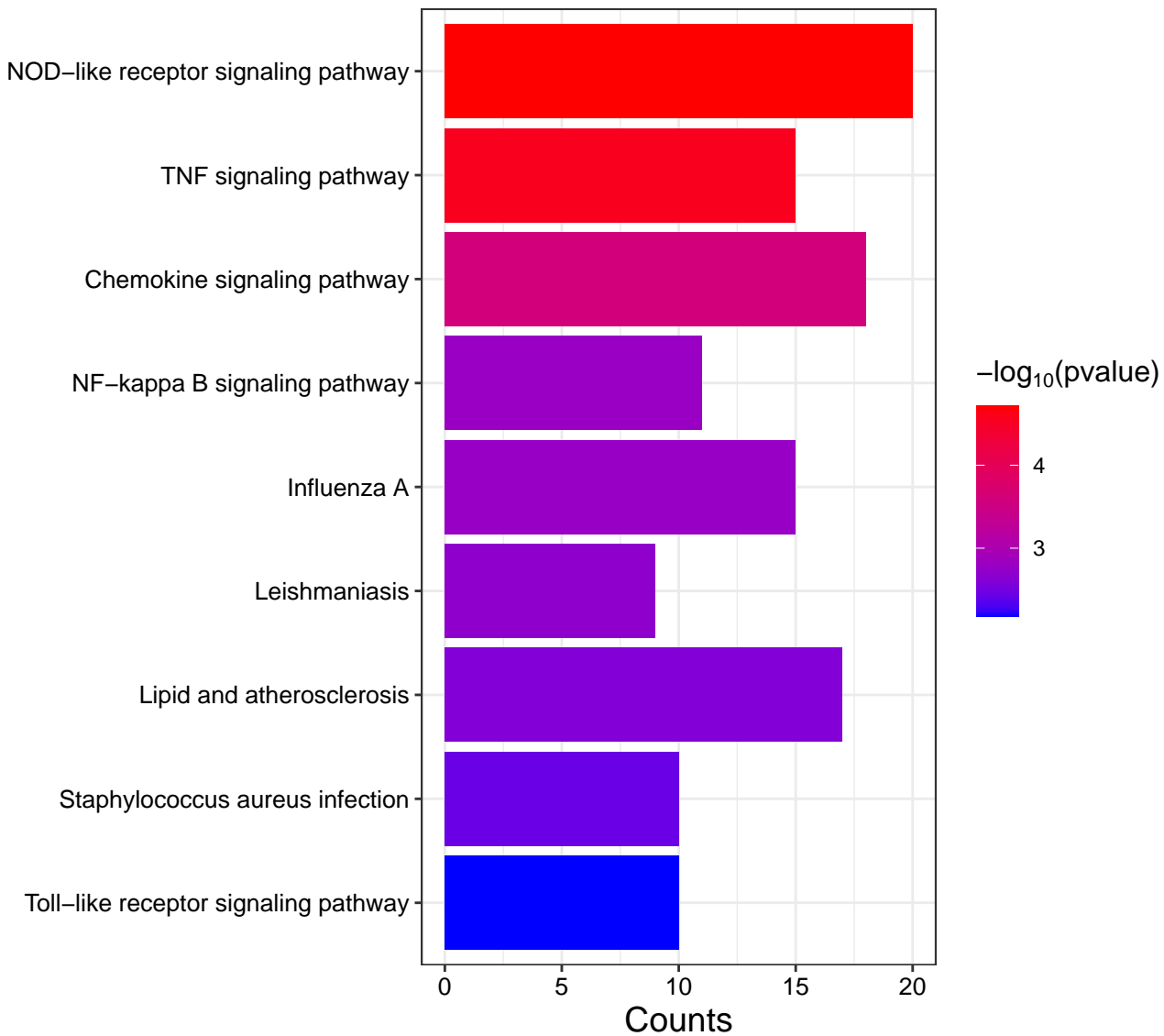

Supplement: Supplementary file 5 [file Data_Sheet_5.ZIP › Raw data/image files/KEGG_bar.pdf]

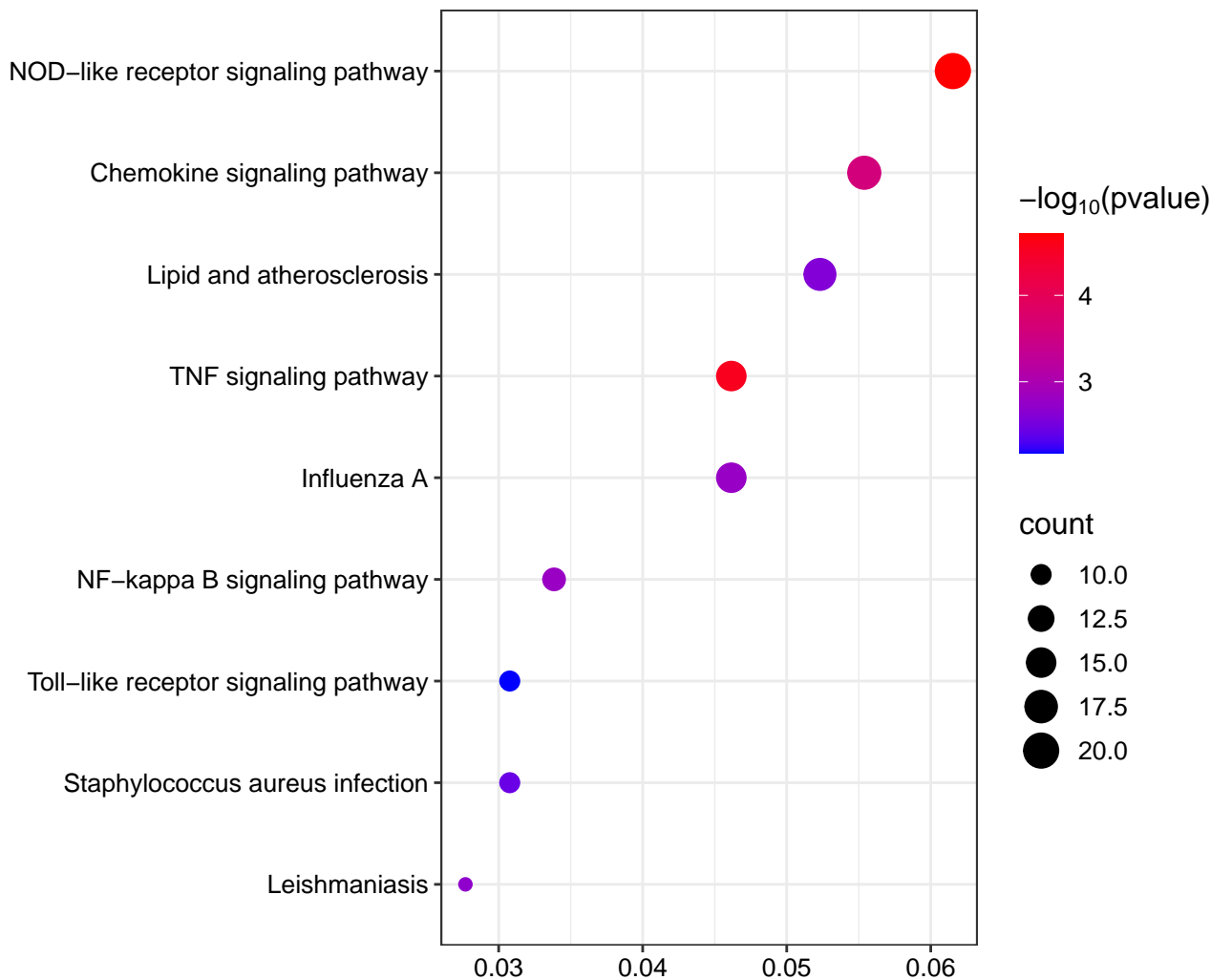

Supplement: Supplementary file 5 [file Data_Sheet_5.ZIP › Raw data/image files/KEGG_bubble.pdf]

Coefficients

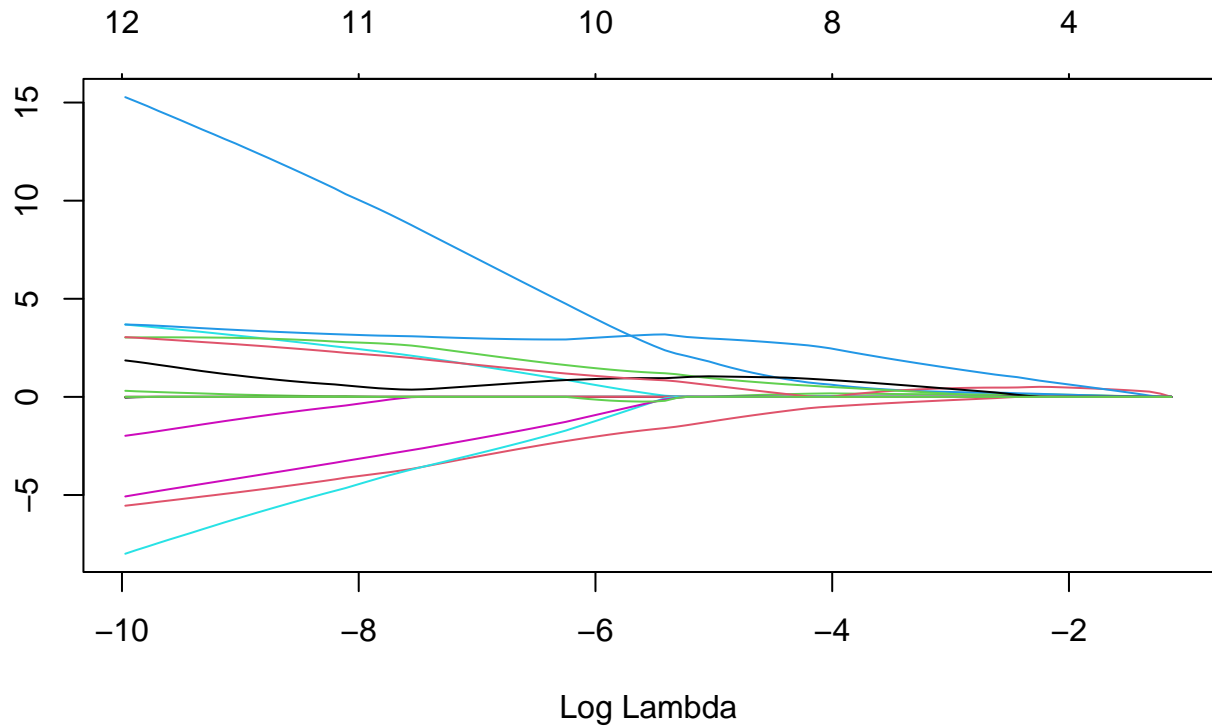

Supplement: Supplementary file 5 [file Data_Sheet_5.ZIP › Raw data/image files/LASSO-1.pdf]

Binomial Deviance

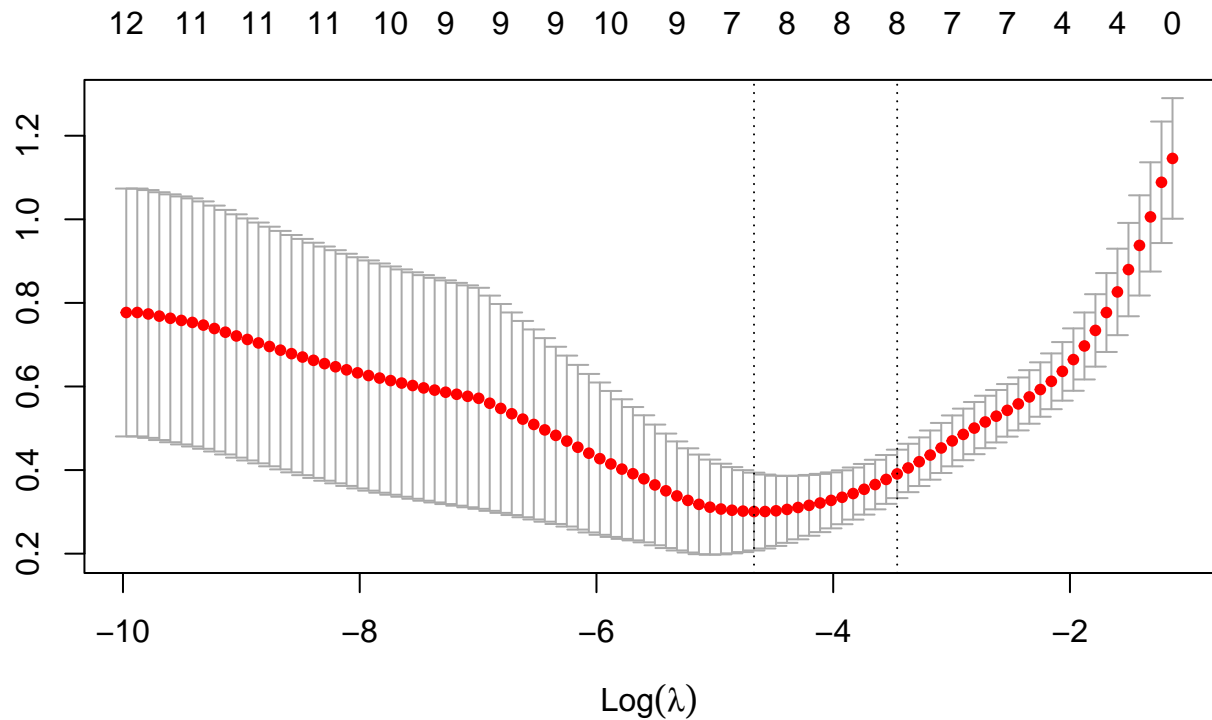

Supplement: Supplementary file 5 [file Data_Sheet_5.ZIP › Raw data/image files/LASSO-2.pdf]

Module–trait relationships

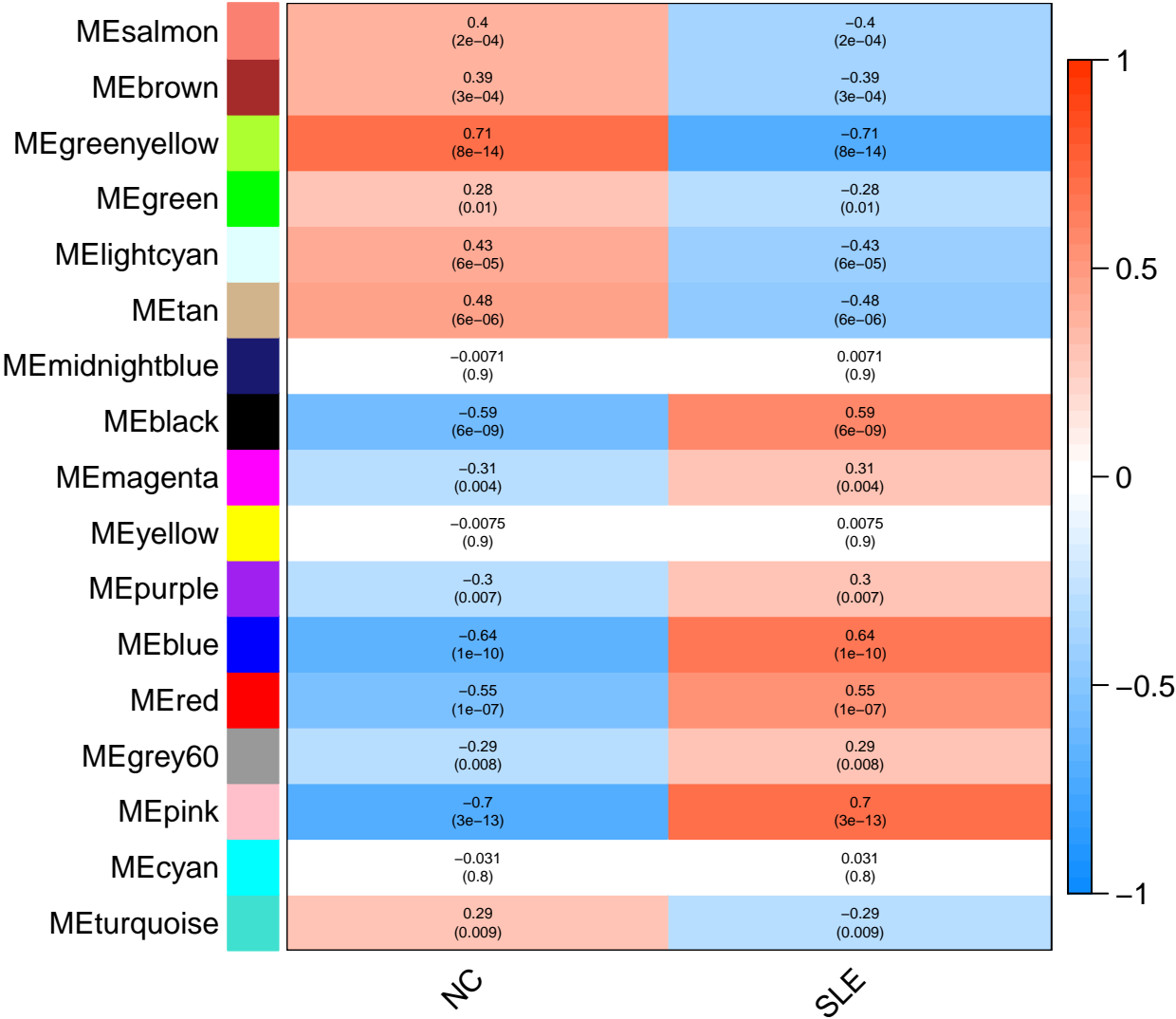

Supplement: Supplementary file 5 [file Data_Sheet_5.ZIP › Raw data/image files/ModuleTraitHeatmap-rawcolor.pdf]

Group 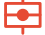 NC 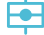 SLE

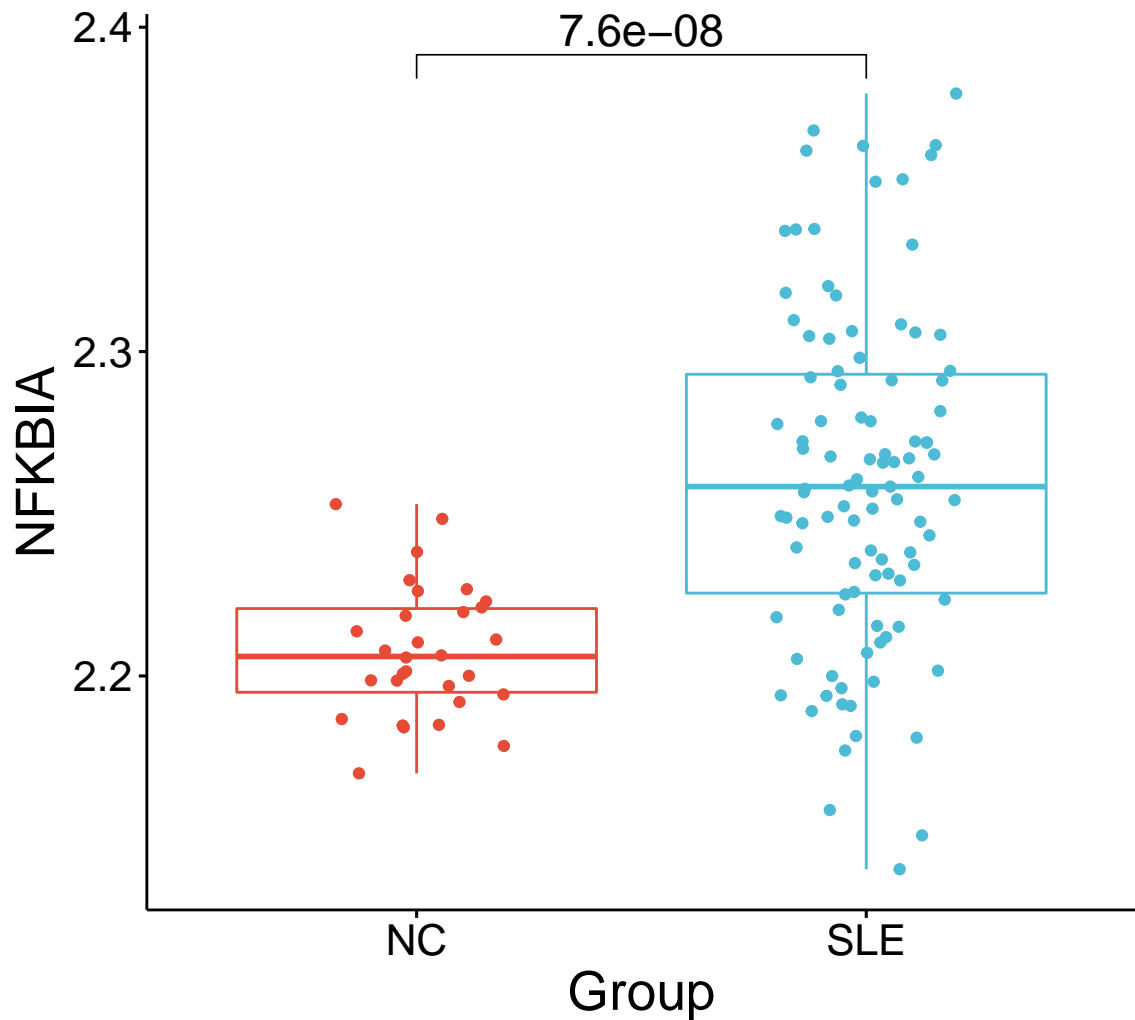

Supplement: Supplementary file 5 [file Data_Sheet_5.ZIP › Raw data/image files/NFKBIA expression of validating dataset.pdf]

Group 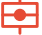 NC 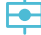 SLE

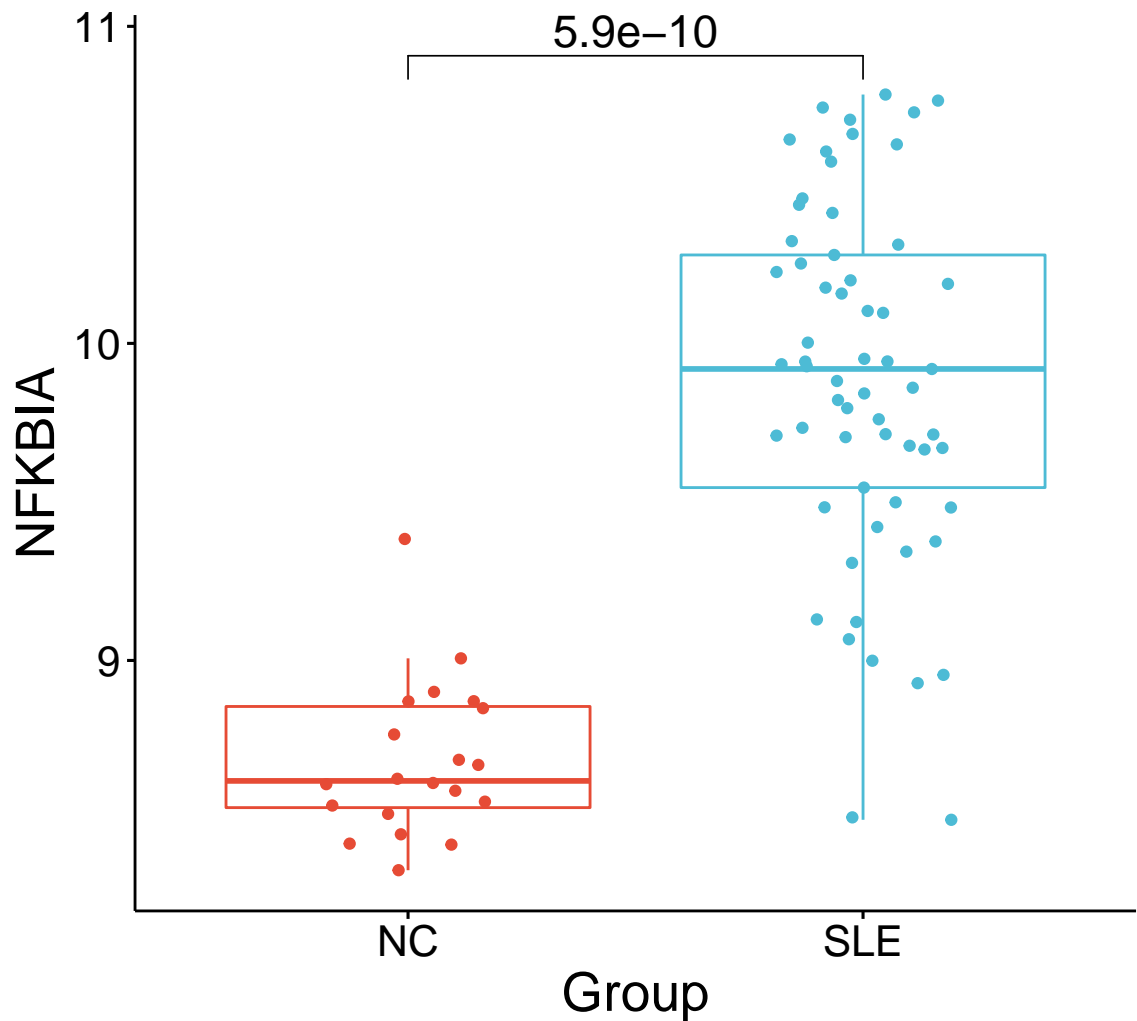

Supplement: Supplementary file 5 [file Data_Sheet_5.ZIP › Raw data/image files/NFKBIA expression of training dataset.pdf]

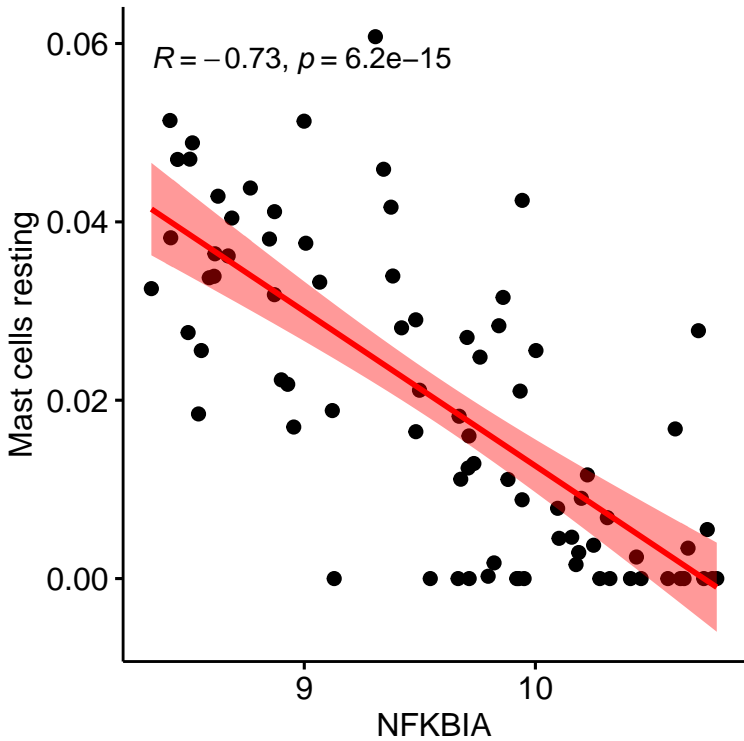

Supplement: Supplementary file 5 [file Data_Sheet_5.ZIP › Raw data/image files/NFKBIA-mast cell resting.pdf]

# NFKBIA

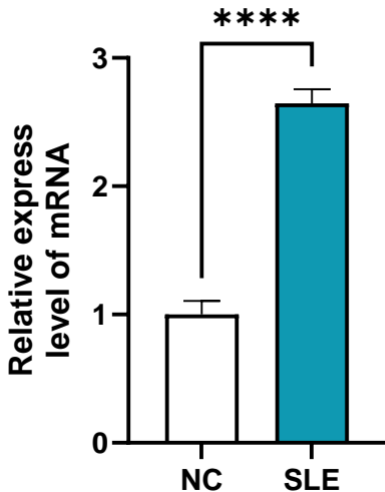

Supplement: Supplementary file 5 [file Data_Sheet_5.ZIP › Raw data/image files/NFKBIA-pcr.pdf]

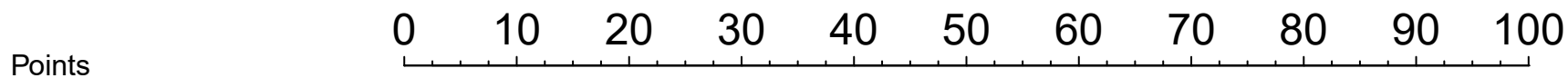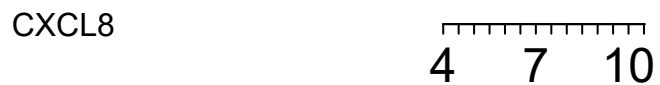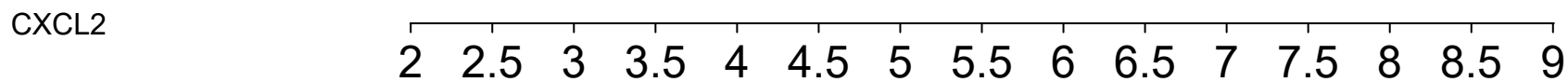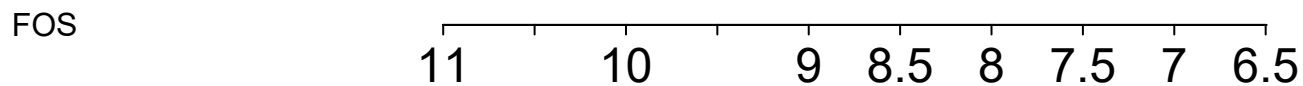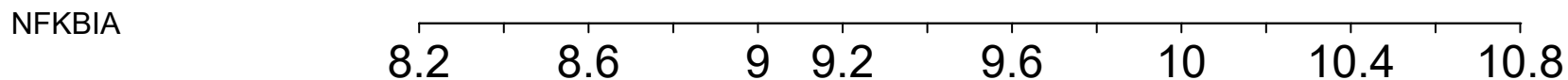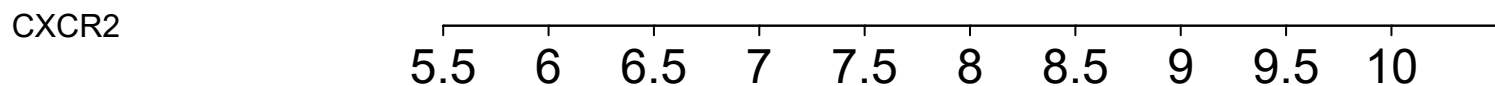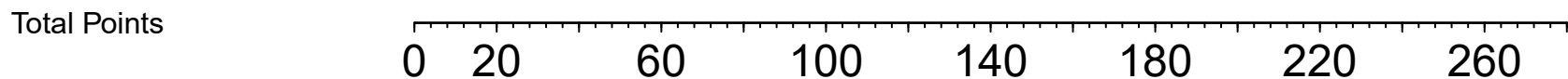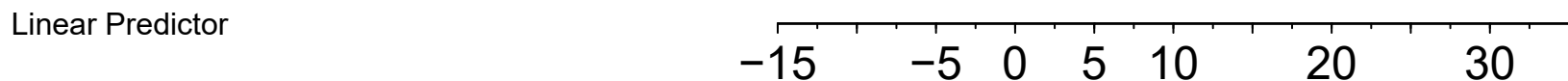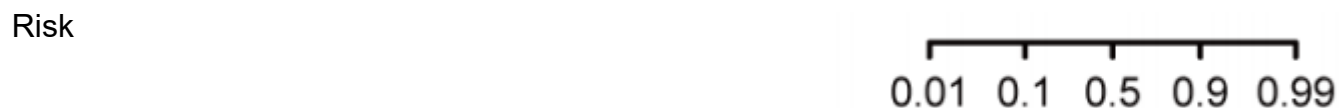

Supplement: Supplementary file 5 [file Data_Sheet_5.ZIP › Raw data/image files/nomogram.pdf]

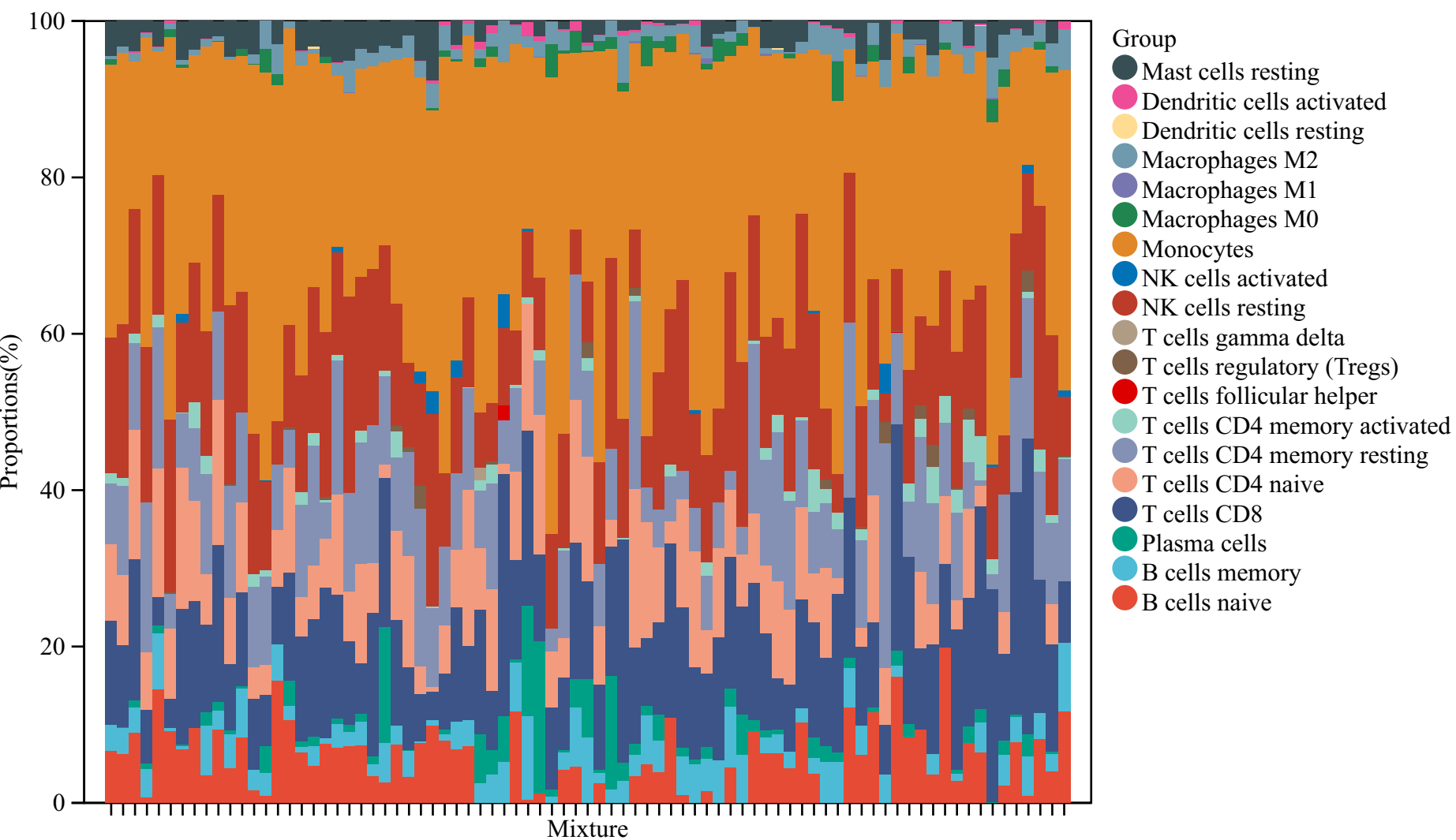

Supplement: Supplementary file 5 [file Data_Sheet_5.ZIP › Raw data/image files/Relative percentage of immune cell.pdf]

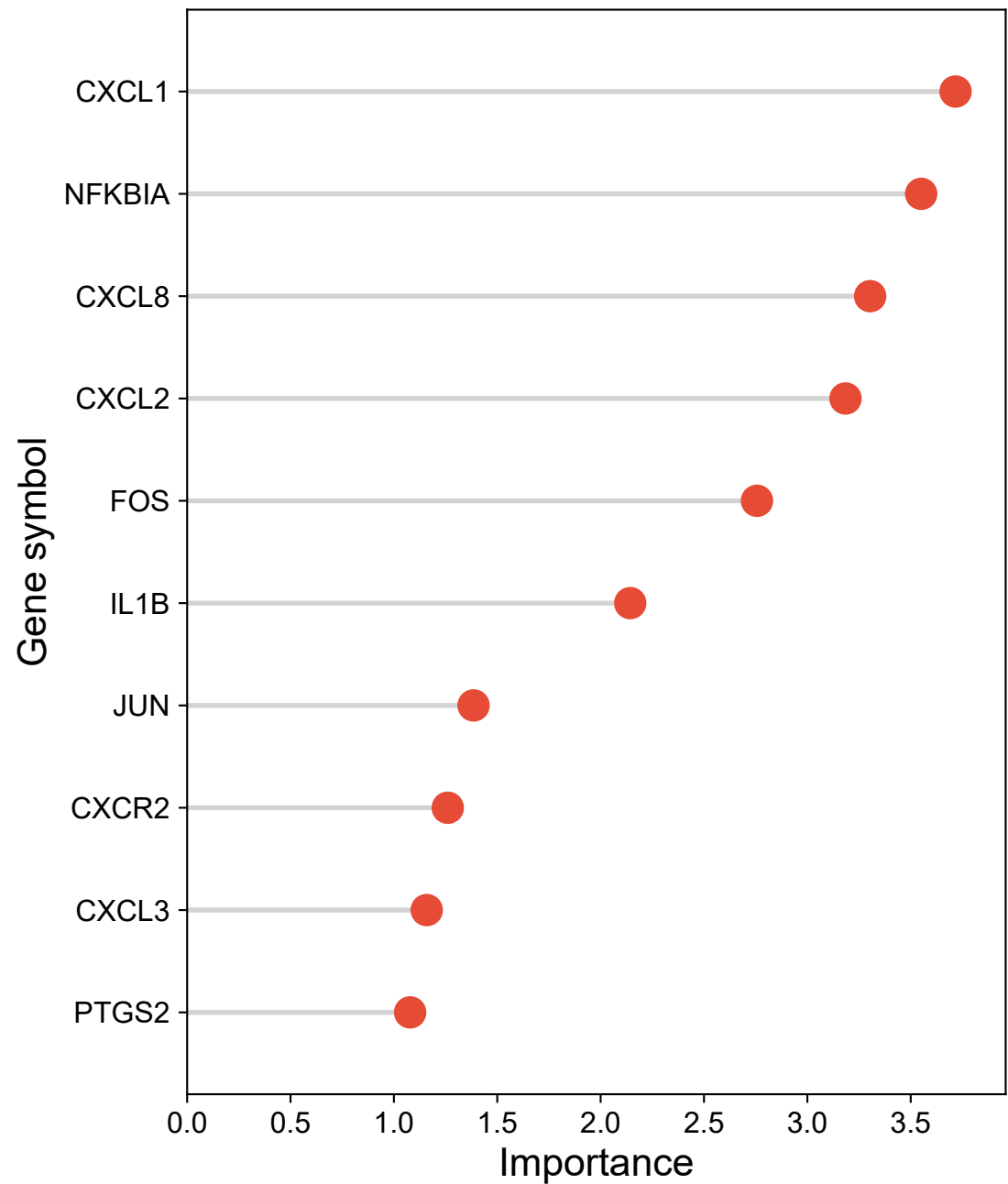

Supplement: Supplementary file 5 [file Data_Sheet_5.ZIP › Raw data/image files/RF.pdf]

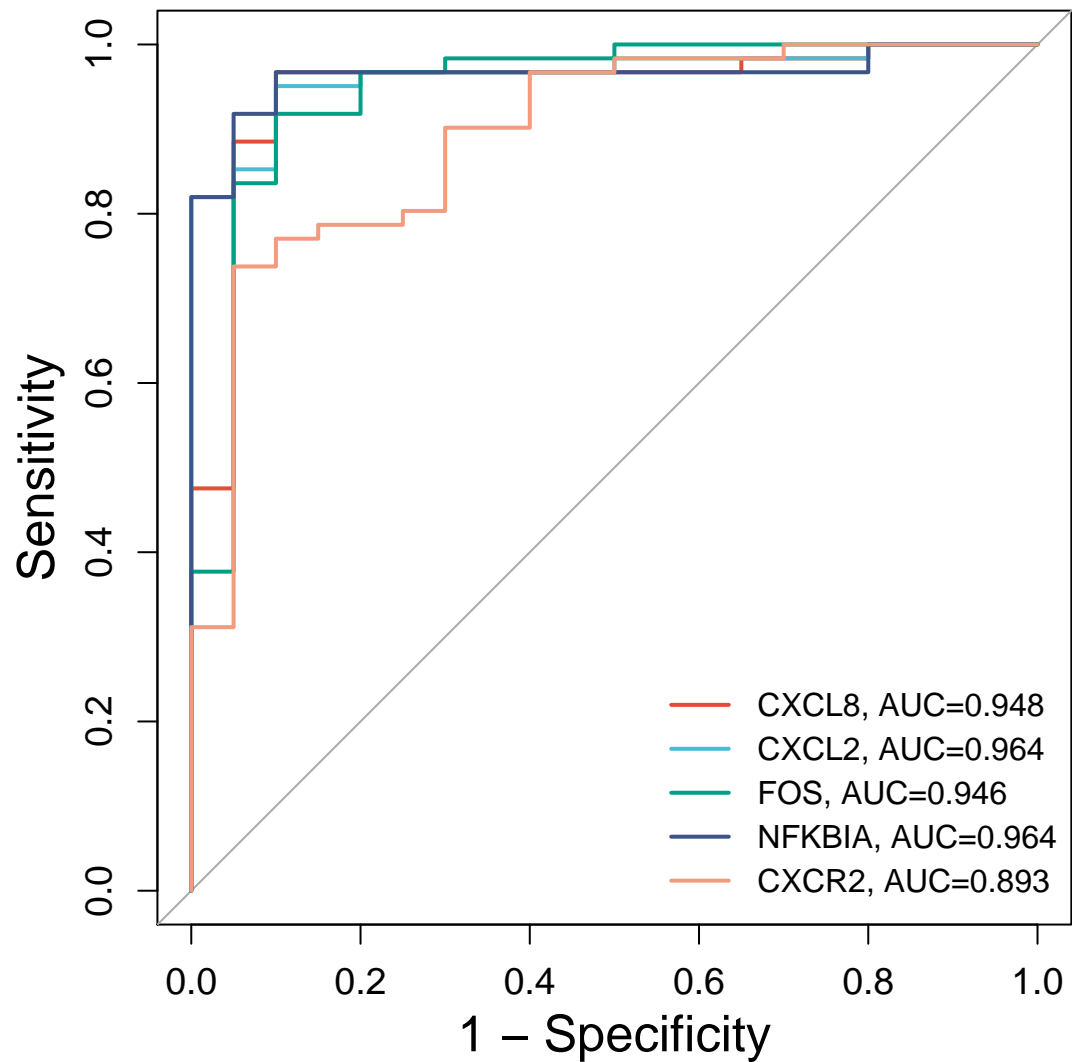

Supplement: Supplementary file 5 [file Data_Sheet_5.ZIP › Raw data/image files/ROC of training dataset.pdf]

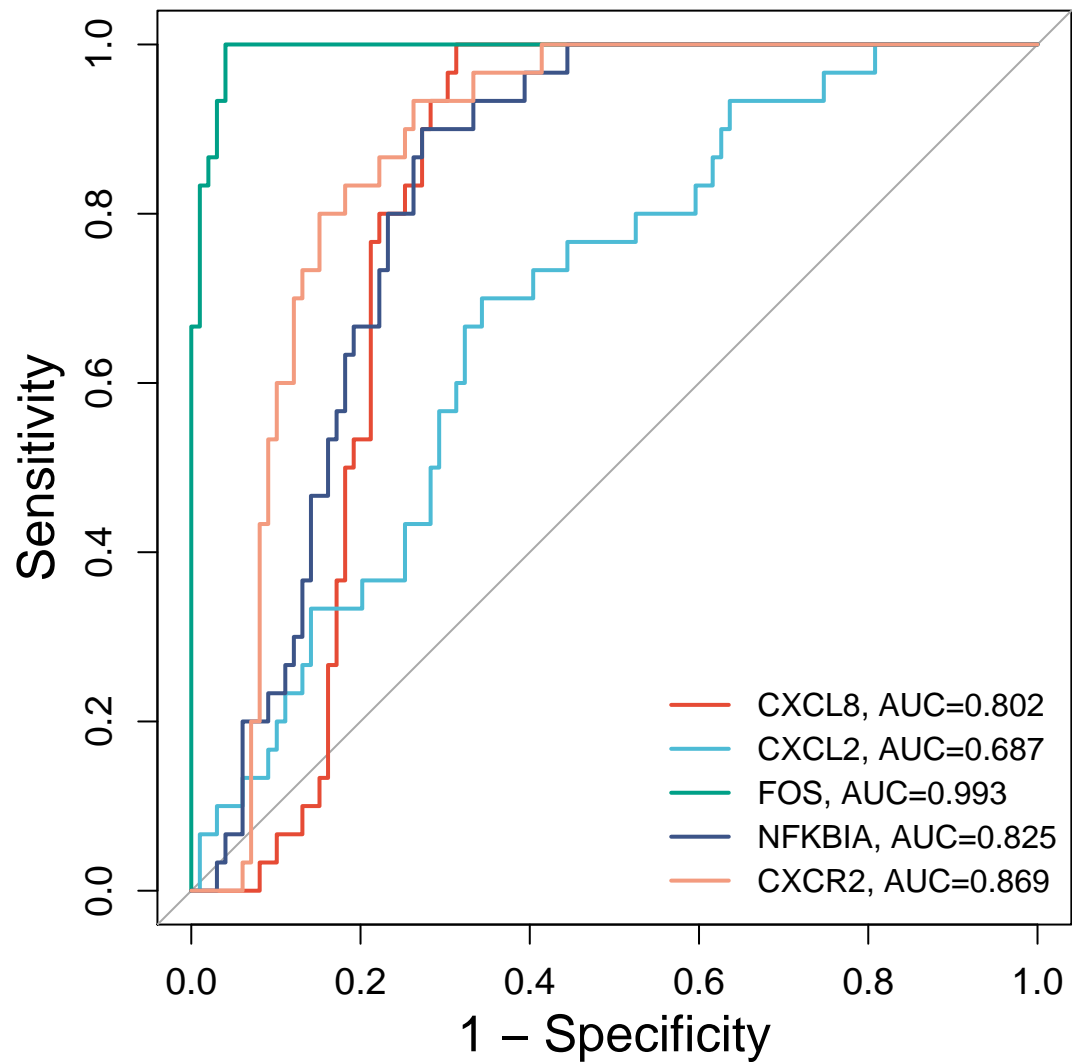

Supplement: Supplementary file 5 [file Data_Sheet_5.ZIP › Raw data/image files/ROC of validating dataset.pdf]

ModuleMembership vs GeneSignificance

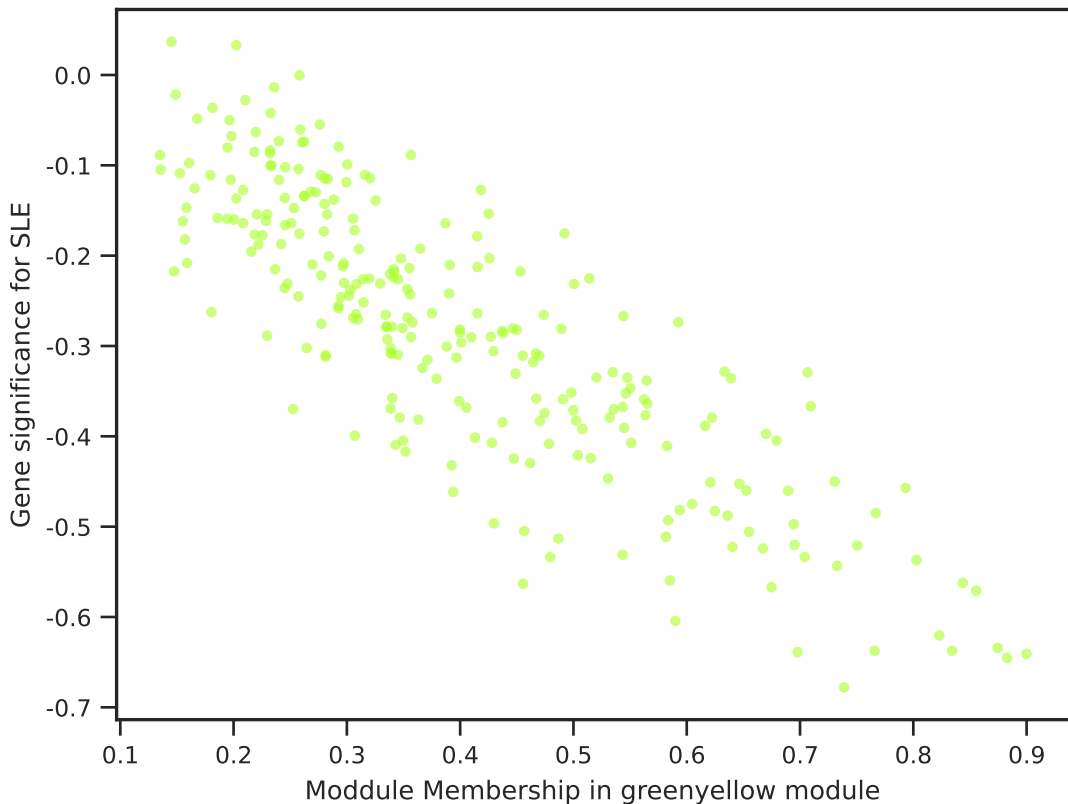

Supplement: Supplementary file 5 [file Data_Sheet_5.ZIP › Raw data/image files/SLE_greenyellow.pdf]

ModuleMembership vs GeneSignificance

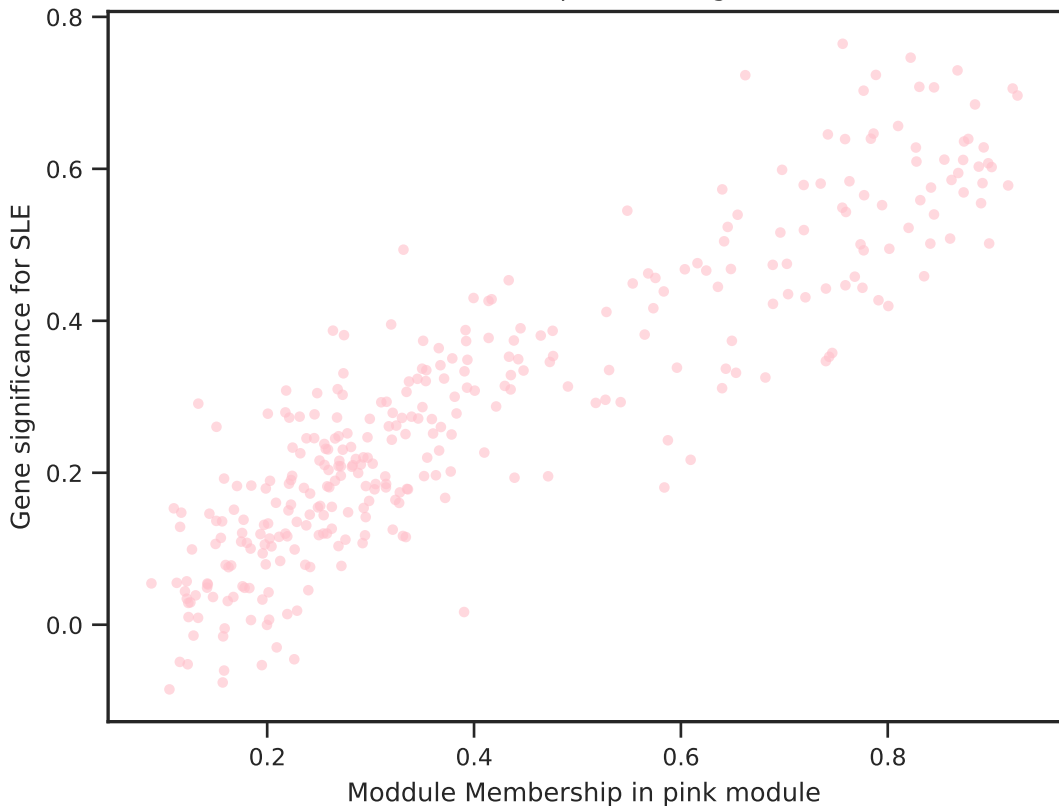

Supplement: Supplementary file 5 [file Data_Sheet_5.ZIP › Raw data/image files/SLE_pink.pdf]

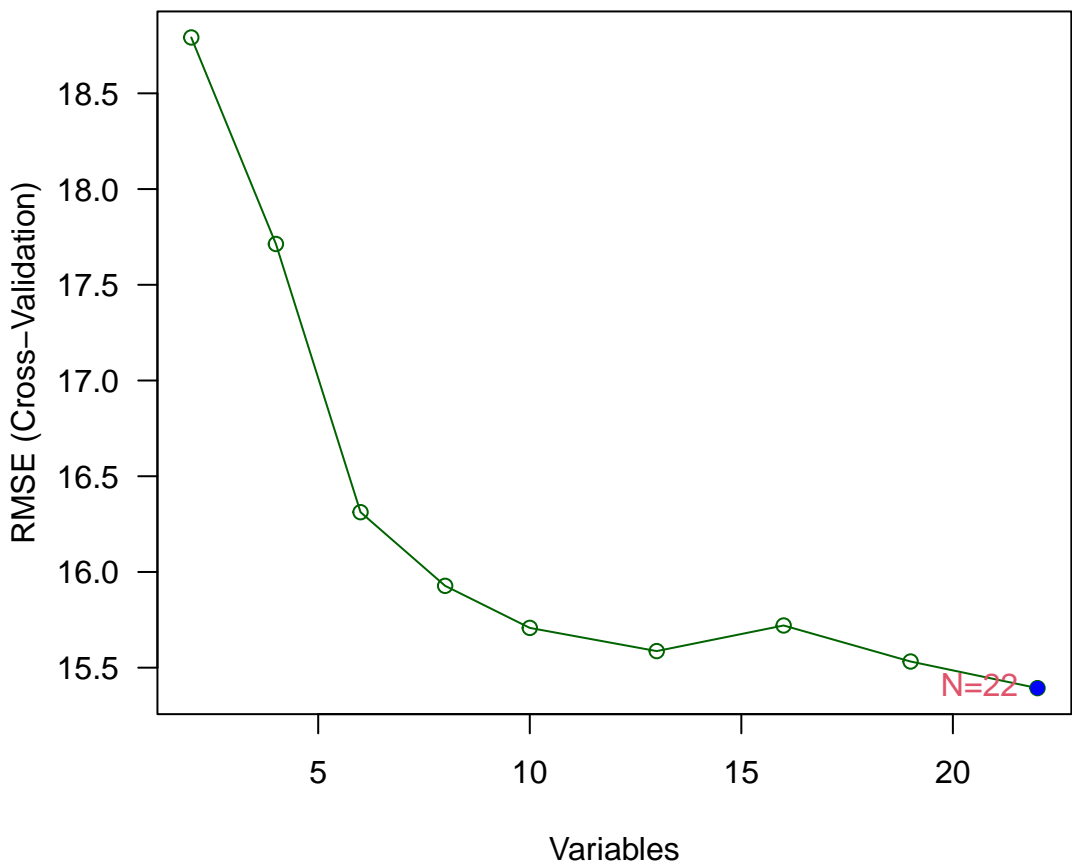

Supplement: Supplementary file 5 [file Data_Sheet_5.ZIP › Raw data/image files/SVM-RFE.pdf]

# Cluster Dendrogram

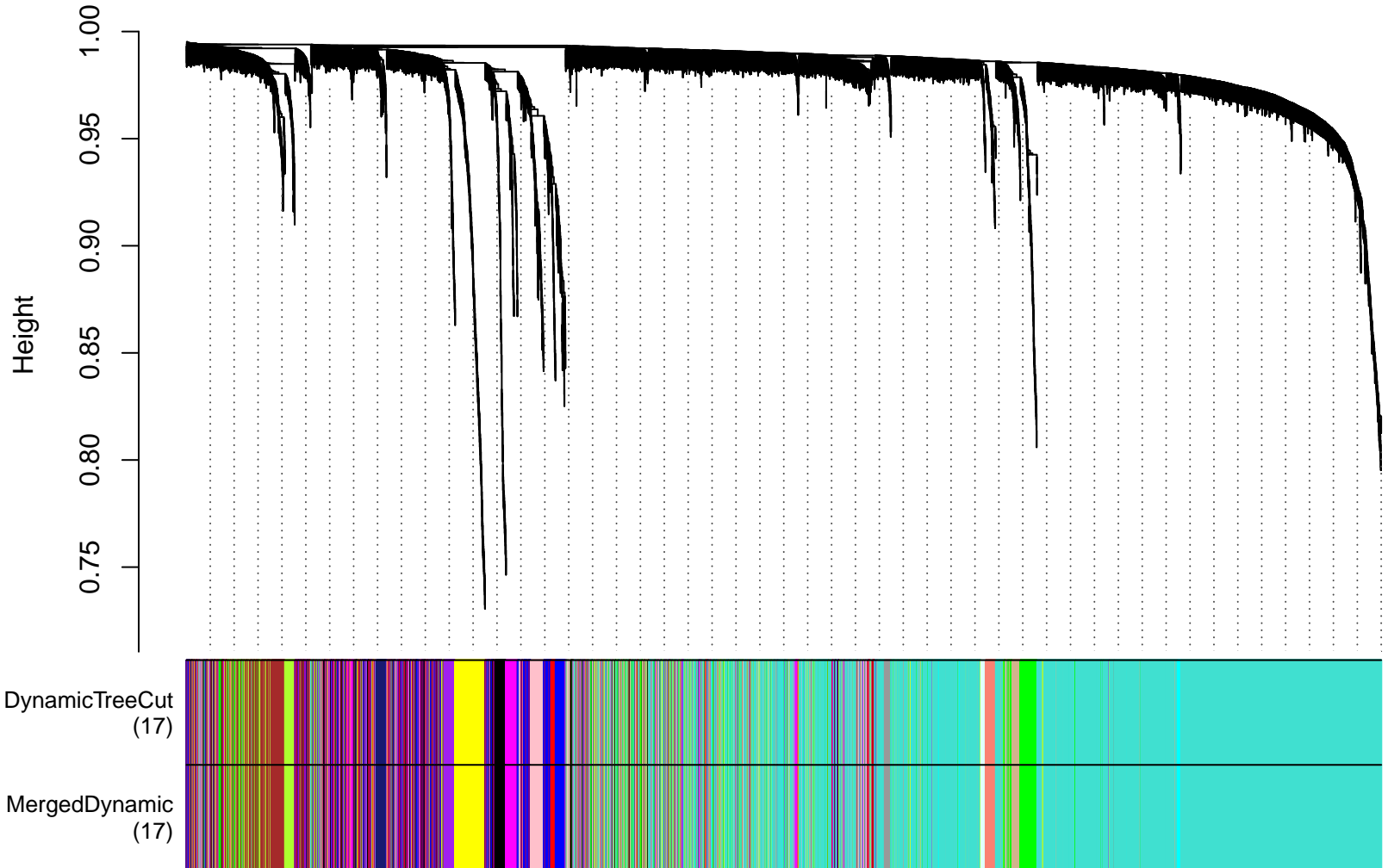

Supplement: Supplementary file 5 [file Data_Sheet_5.ZIP › Raw data/image files/The clustering dendrogram of the WGCNA network.pdf]

Expression

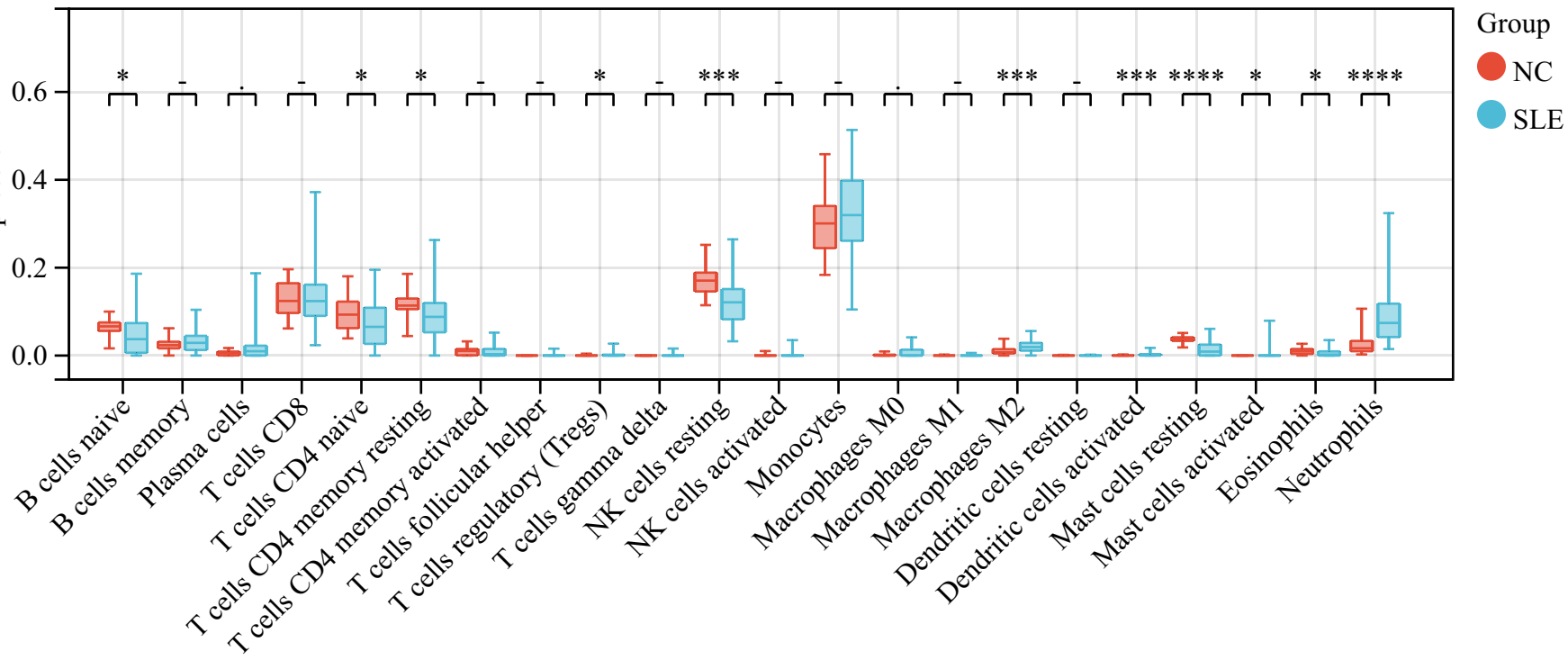

Supplement: Supplementary file 5 [file Data_Sheet_5.ZIP › Raw data/image files/the differences in the levels of immune cells in SLE and NC.pdf]

# Scale independence

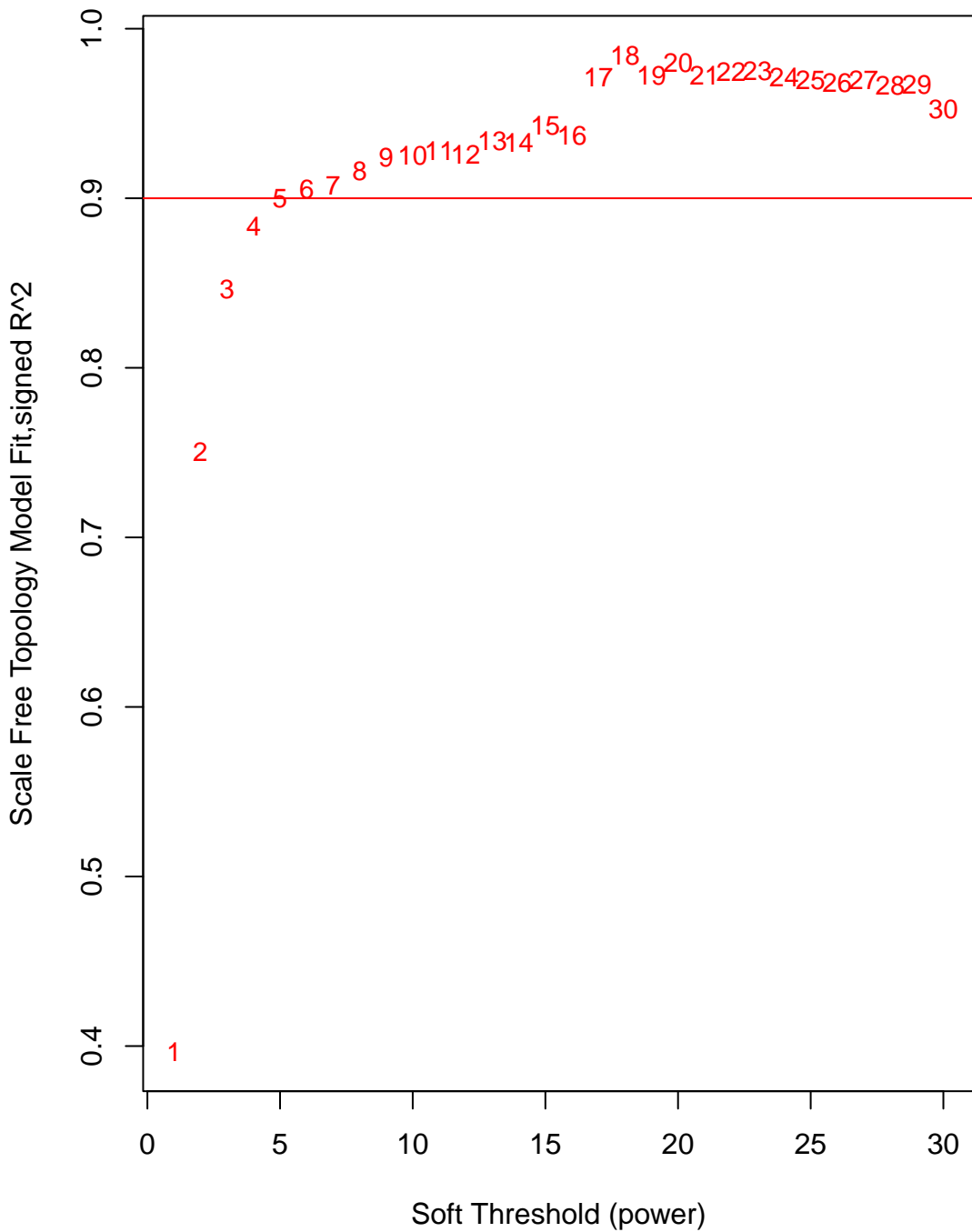

# Mean connectivity

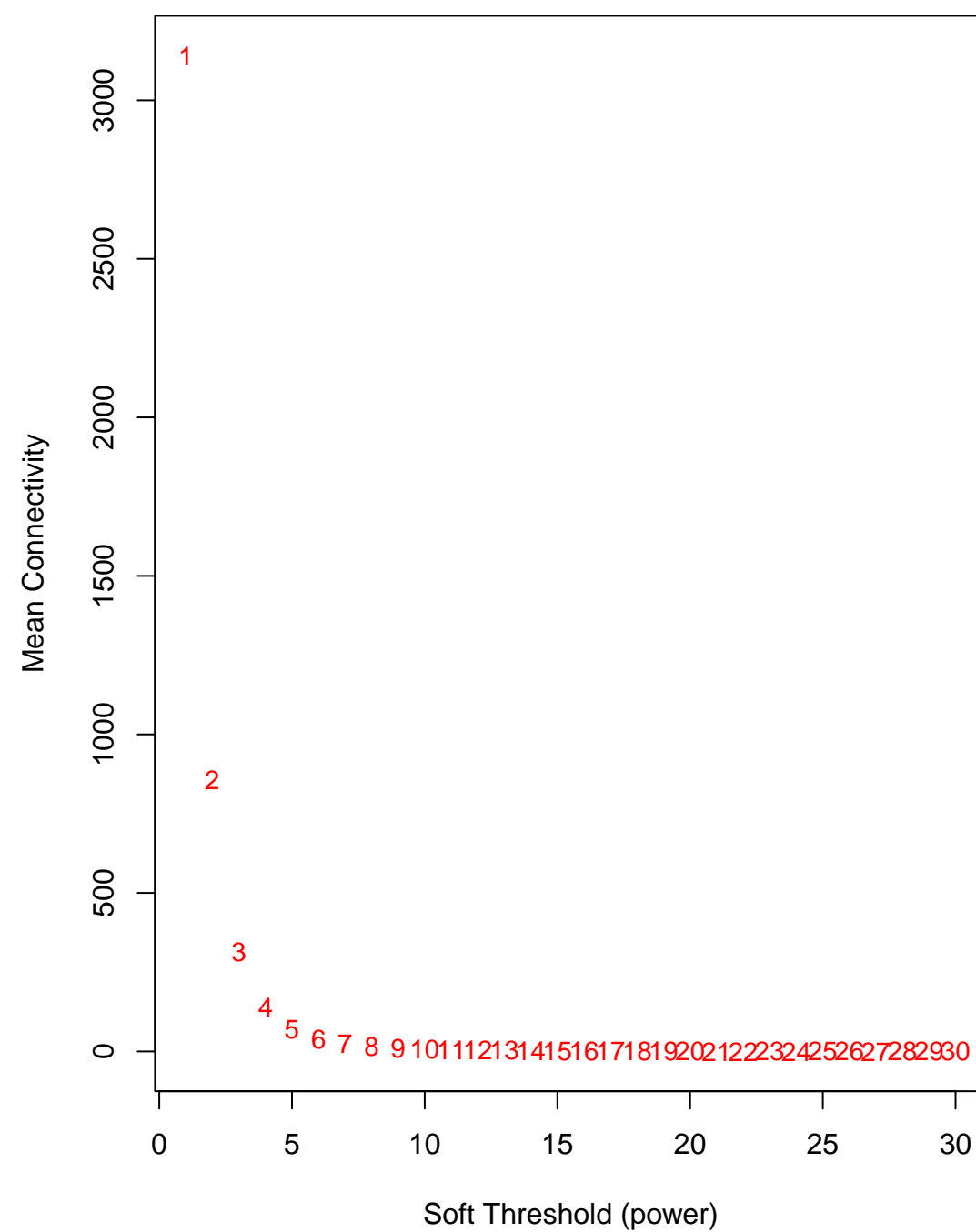

Supplement: Supplementary file 5 [file Data_Sheet_5.ZIP › Raw data/image files/The soft threshold and mean connectivity of the WGCNA network.pdf]

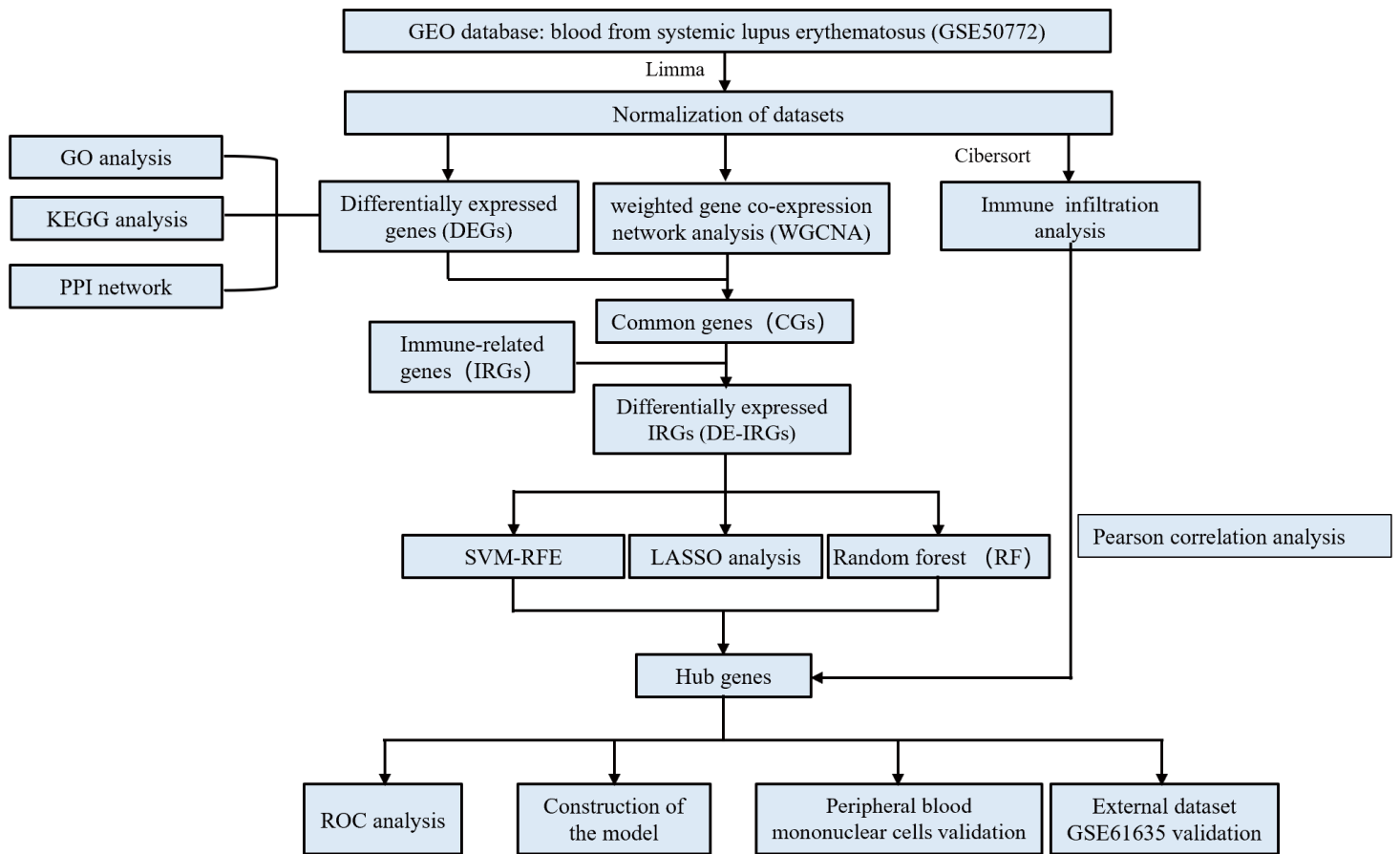

Supplement: Supplementary file 5 [file Data_Sheet_5.ZIP › Raw data/image files/The specific flowchart of this study .pdf]

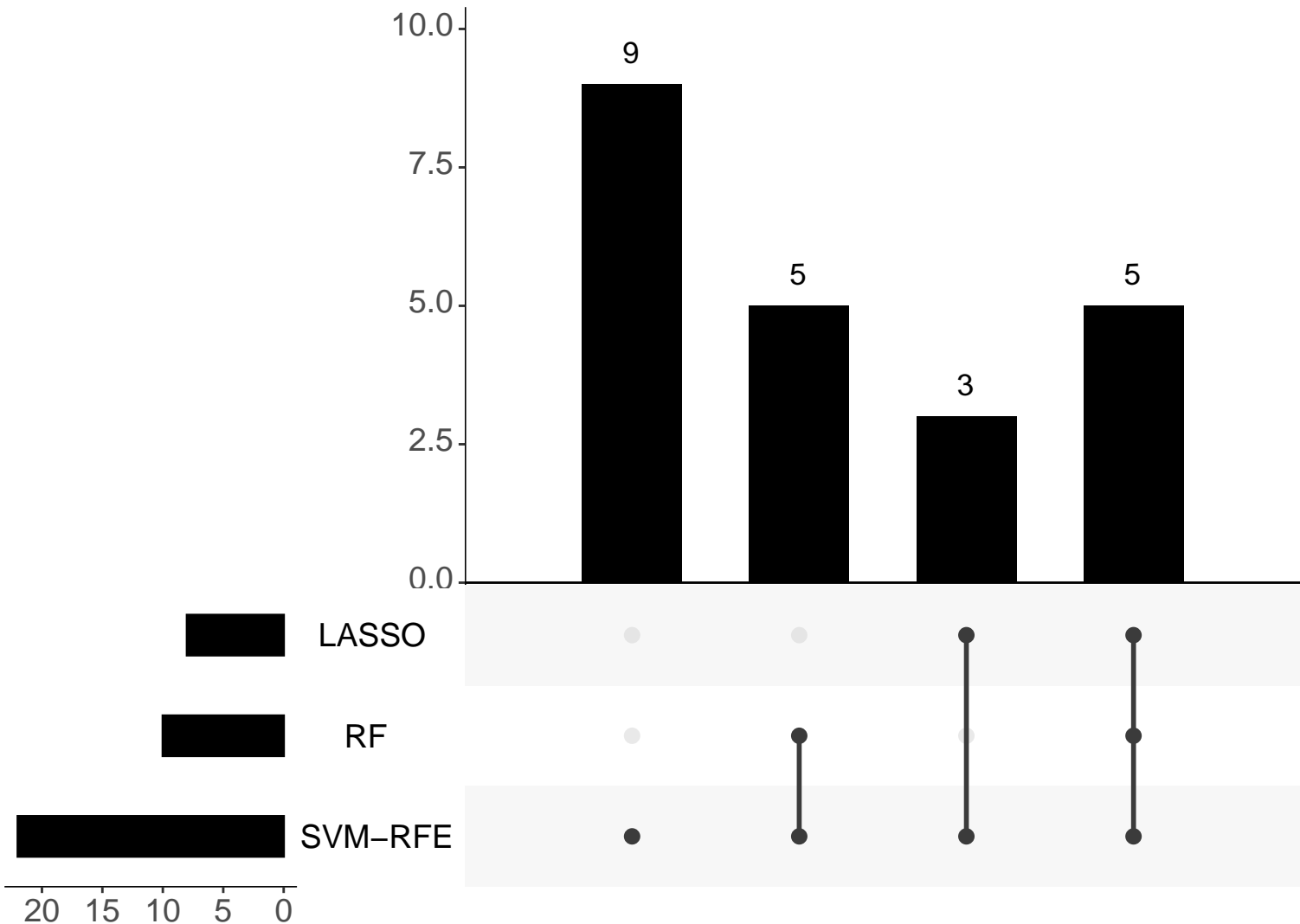

Supplement: Supplementary file 5 [file Data_Sheet_5.ZIP › Raw data/image files/UPset.pdf]

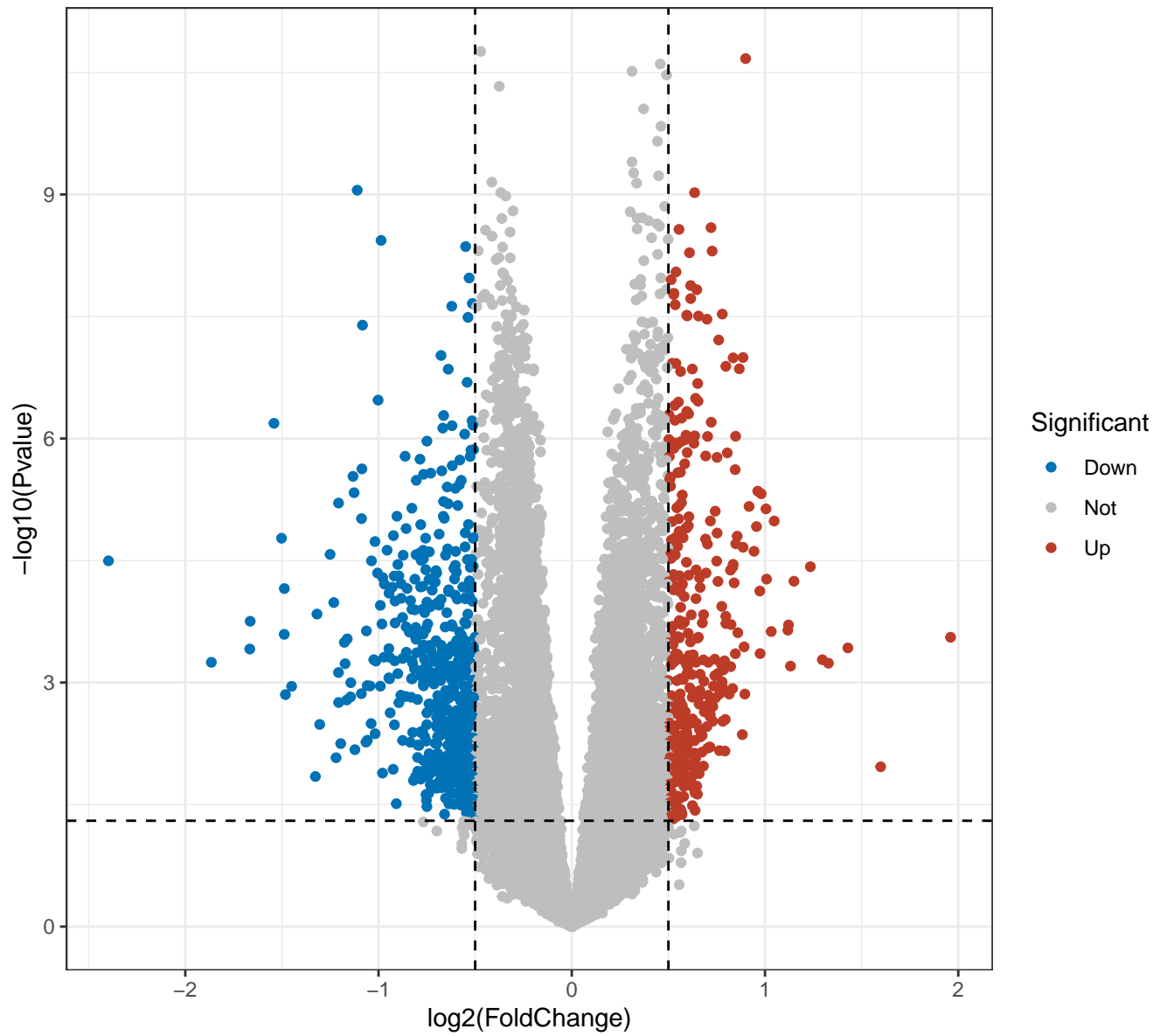

Supplement: Supplementary file 5 [file Data_Sheet_5.ZIP › Raw data/image files/volcano.pdf]
